# Worldwide Prevalence of Epstein–Barr Virus in Patients with Burkitt Lymphoma: A Systematic Review and Meta-Analysis

**DOI:** 10.3390/diagnostics13122068

**Published:** 2023-06-15

**Authors:** Mutaz Jamal Al-Khreisat, Nor Hayati Ismail, Abedelmalek Tabnjh, Faezahtul Arbaeyah Hussain, Abdul Aziz Mohamed Yusoff, Muhammad Farid Johan, Md Asiful Islam

**Affiliations:** 1 Department of Haematology, School of Medical Sciences, Universiti Sains Malaysia, Kubang Kerian 16150, Kelantan, Malaysia; 2 Department of Applied Dental Sciences, Faculty of Applied Medical Sciences, Jordan University of Science and Technology, Irbid 22110, Jordan; 3 Department of Pathology, School of Medical Sciences, Universiti Sains Malaysia, Kubang Kerian 16150, Kelantan, Malaysia; 4 Department of Neurosciences, School of Medical Sciences, Universiti Sains Malaysia, Kubang Kerian 16150, Kelantan, Malaysia; 5 WHO Collaborating Centre for Global Women’s Health, Institute of Metabolism and Systems Research, College of Medical and Dental Sciences, University of Birmingham, Birmingham B15 2TT, UK

**Keywords:** Burkitt lymphoma, Epstein–Barr, meta-analysis

## Abstract

Burkitt lymphoma (BL) is a form of B-cell malignancy that progresses aggressively and is most often seen in children. While Epstein–Barr virus (EBV) is a double-stranded DNA virus that has been linked to a variety of cancers, it can transform B lymphocytes into immortalized cells, as shown in BL. Therefore, the estimated prevalence of EBV in a population may assist in the prediction of whether this population has a high risk of increased BL cases. This systematic review and meta-analysis aimed to estimate the prevalence of Epstein–Barr virus in patients with Burkitt lymphoma. Using the appropriate keywords, four electronic databases were searched. The quality of the included studies was assessed using the Joanna Briggs Institute’s critical appraisal tool. The results were reported as percentages with a 95% confidence interval using a random-effects model (CI). PROSPERO was used to register the protocol (CRD42022372293), and 135 studies were included. The prevalence of Epstein–Barr virus in patients with Burkitt lymphoma was 57.5% (95% CI: 51.5 to 63.4, *n* = 4837). The sensitivity analyses demonstrated consistent results, and 65.2% of studies were of high quality. Egger’s test revealed that there was a significant publication bias. EBV was found in a significantly high proportion of BL patients (more than 50% of BL patients). This study recommends EBV testing as an alternative for predictions and the assessment of the clinical disease status of BL.

## 1. Introduction

Epstein–Barr virus (EBV) is a pathogenic double-stranded DNA human herpes virus 4 (HHV4). It was first discovered as a human-associated virus by Michael Anthony Epstein and Yvonne Barr in 1964 [1]. The virus consists of a 170–180 kb liner of double-stranded (ds) enveloped DNA with a toroid-shaped protein core, a nucleocapsid with 162 capsomers, and external virus-encoded glycoprotein spikes on the surface of the viral tegument [2]. The EBV genome encodes more than 85 genes, which are involved in the pathogenesis of infection and initiating EBV-associated human disease. There are two major types of EBV: type 1 EBV, which is found worldwide, and type 2 EBV, which is mainly detected in Africa [3]. EBV is the most frequent cause of infectious mononucleosis, with primary infections commonly occurring asymptomatically in teenagers and young adults, especially college students, while in adults, the symptoms are more severe. After primary infection, EBV establishes latent and lytic programs [4,5]. During the latent form of infection, the virus persists in the host cells, while during the lytic phase of infection, new infectious virions are produced [1]. Individuals infected with EBV control the virus’s infectious behavior through cytotoxic immune cell reactions mediated by natural killer (NK) cells and CD8+ T lymphocytes [6,7]. Only a few infected individuals develop chronic EBV-associated pathologies, often due to immune deficiencies, genetic predisposition, and environmental factors [8]. Chronic EBV infections are mainly in the epithelial and lymphocytic cells, which have been associated with malignant diseases [1,9]. EBV is very common in the general population; however, only a minority of infected people experience EBV-related pathologies, suggesting that additional risk factors, such as immune deficiencies, genetic predisposition, and environmental factors, are also crucial in the development of these pathologies [10,11,12]. EBV-associated malignancies express different EBV latent gene products, which are involved in the anti-apoptotic functions of B cells and interfere with innate and adaptive immunity, allowing infected cells to escape immune surveillance. Burkitt lymphoma (BL) is a highly aggressive B-cell non-Hodgkin’s lymphoma that is characterized by the translocation and dysregulation of the proto-oncogene MYC as well as hypermutated immunoglobulin gene sequences [13]. BL is derived from germinal center B cells [14]. Histologically, BL demonstrates sheets of monomorphic medium-sized B cells with basophilic cytoplasm, numerous mitoses, and frequent apoptotic bodies. Macrophages are scattered among tumor cells, giving BL a distinctive histologic appearance called the starry sky pattern. Tumor cells express membrane immunoglobulin (Ig) M, Ig light chain, B-cellular antigen, B-cell lymphoma (BCL) protein 6, and a cluster of differentiation (CD) 10, 19, 20, and 22, while showing negative findings for CD 5, 23, and BCL 2 [15,16,17]. The EBV status of tumors affects the expression of the Epstein–Barr virus (EBV)/C3d receptor and CD21. In essence, all cases of endemic BL are EBV-positive and express CD21, whereas the majority of non-endemic BL among patients who are non-immunosuppressed are EBV-negative and do not express CD21 [18].

The initial BL case was reported in the early 20th century. Denis Burkitt observed widespread childhood tumors in Uganda, which were characterized by malignant growths in the jaw and within the abdominal cavity [19,20]. The World Health Organization (WHO) classified three clinical variants of BL based on cancer epidemiology: endemic, sporadic, and immunodeficiency-associated. These variants are histologically identical and have similar clinical behavior [21]. Endemic BL (eBL) presents in the jaw in younger children and abdominally in older children in malaria-endemic regions, predominantly in sub-Saharan Africa and Papua New Guinea. eBL has a 2:1 male-to-female ratio and a median age of 6 years [22,23].

eBL is mainly localized to geographical areas where Plasmodium falciparum malaria is holoendemic. Chronic B-cell activation or promotion of EBV’s oncogenic potential in the presence of malarial co-infection has been postulated to increase oncogenesis [24,25,26]. Sporadic BL (sBL) is distributed worldwide, with the majority of cases occurring in the United States and Western Europe. sBL is more frequent in children, accounting for 20% to 30% of lymphomas in this age group. Adults with sporadic BL are uncommon, accounting for less than 1% of NHL cases in the United States [27]. BL presents within the abdominal region, lymph nodes, and can also be extranodal. The third variant is HIV-associated BL (ID-BL), which is diagnosed at the early stage of HIV infection and prior to CD4+ T-cell decreases [28].

EBV varies in detection among the three clinical variants of BL. Most endemic BLs are associated with EBV, which suggests that the virus has a direct role in lymphoma pathogenesis. About 95% of eBL detect EBV [28], whereas only about 10–30% of EBV is detected in sBL [21], and 20–40% of EBV positives are detected in ID-BL [28].

EBV plays a critical role in the onset of multiple sclerosis, according to growing data from several study fields. It has been proposed that multiple sclerosis (MS) depends on the early immune response to EBV infection because the severity of the EBV primary infection is strongly associated with the onset of MS many years later. The inability to control this infection might result in the colonization of resident memory B-cell and T-cell follicles in CNS-accessible regions, such as tertiary lymphoid structures, which are particularly prone to triggering immunological disease in the CNS. The period of infection is probably a factor in the immune system’s elimination of the viruses, autoreactive T cells, and antibodies that are directed against CNS components [29,30].

EBV-related malignancies are linked to a latent form of infection, in which EBV expresses a limited set of proteins called EBV transcription programs (ETPs) in every tumor cell, including six nuclear antigens (EBNAs), three latent membrane proteins (LMPs), and untranslated RNA called EBV-encoded small RNA (EBERs), which can mediate cellular transformation [31]. EBV infects primary B cells and induces them to proliferate, by expressing viral genes that were identified as EBNA1, EBNA2, ENBA3A, EBNA3C, and LMP1, which are involved in the latency phase of EBV infection [1]. Additional genes that are included in the transforming B cells are LMP2, viral miRNAs, the small non-coding *RNA EBER*, *BZLF1*, and *BRLF1* [32].

The three latency programs that EBV can display are either Latency I, Latency II, or Latency III. A specific, limited set of viral proteins and RNAs are produced by each latency program (Table 1) [33,34].

Studies showed that BL expresses high levels of MYC, and more than 90% show the translocation of the *MYC* oncogene (8q24) onto the immunoglobulin heavy chain (IgH) (14q34). The chromosomal breakpoints of both MYC and IgH vary between sBL and eBL, giving rise to different aetiologic drivers [35]. A translocation of the MYC gene on chromosome 8, including genetic material from chromosomes 2, 14, or 22, is the classic etiology of BL. The majority of translocations (around 80%) involve the Ig heavy chain on chromosome 14, t(8;14), whereas 15% involve the kappa light chain on chromosome 2, t(2;8), and 5% involve the lambda light chain on chromosome 22 [36,37].

EBV-associated malignancies are diagnosed primarily by a biopsy of the primary tumor, with an EBER in situ hybridization test to confirm the presence of EBV [38]. However, due to the difficulty in obtaining a sample of the tumor or poor patient condition, performikng a biopsy might be challenging [39].

Many studies of EBV-associated lymphoma reveal that EBV-DNA may be found in the plasma of most patients with EBV-related malignancies [40]. DNA from EBV-associated lymphoma is derived as naked DNA fragments from apoptotic or necrotic tumor cells [35,38], whereas it is undetectable in non-EBV-associated tumors or healthy people [24]. Although plasma EBV DNA has recently become more important in the diagnosis and management of EBV-associated cancers [41], particularly Hodgkin’s lymphoma (HL) [41,42] and nasopharyngeal carcinoma [43,44], there are limited data on the diagnostic and prognostic significance of plasma EBV DNA for BL. In order to identify EBV in various types of samples, methods such as the heterophile antibody test, immunofluorescence assays, enzyme immunoassays, Western blot, and polymerase chain reaction (PCR) are used. The use of PCR to determine the EBV viral load is becoming more popular in the diagnosis of EBV-related diseases [45].

Artificial intelligence (AI) is now advancing quickly, and its application in medicine is becoming more relevant. To predict or classify based on input data, AI integrates computer science and databases. Machine learning and deep learning are two types of AI used in the medical field to evaluate medical data and acquire an understanding of the pathogenesis of diseases. Recently, an AI application used for EBV has been developed, such as a deep-learning-based EBV prediction method from H&E-stained whole-slide images (WSI) in gastric cancer [46], and deep-learning-based classifiers to detect microsatellite instability and EBV status directly from hematoxylin-and-eosin-stained histological slides [47]. In BL, artificial neural networks and various types of machine learning were used to analyze the gene expression and protein levels by immunohistochemistry of several hematological neoplasia and pan-cancer series in order to predict patients’ survival and the disease subtype classification with a high accuracy [48]. There is no systematic review and meta-analysis of the prevalence of EBV in patients with BL that we are aware of. As a result, the goal of this systematic review and meta-analysis was to determine the prevalence of EBV in patients with BL, which helps in predicting whether populations are at high risk of increasing the number of BL cases corresponding to EBV infection.

## 2. Materials and Methods

### 2.1. Reporting Guidelines and Protocol Registration

This systematic review and meta-analysis were carried out according to the Preferred Reporting Items for Systematic Reviews and Meta-Analyses (PRISMA) [49] and Meta-analysis of Observational Studies in Epidemiology (MOOSE) [50] guidelines. This study protocol (PROSPERO: CRD42022372293) was submitted to the International Prospective Registry of Systematic Reviews database at the University of York, York, UK.

### 2.2. Eligibility Criteria

The study looked for published studies on the prevalence of Epstein–Barr virus among Burkitt lymphoma patients. The screening was carried out to find possible studies that looked at the presence of EBV in Burkitt lymphoma patients without any restrictions.

### 2.3. Literature Search

In total, 3981 studies were retrieved from four electronic databases: PubMed, Scopus, Web of Science, and Google Scholar. The most recent search was in January 2021, for studies on the prevalence of Epstein–Barr viruses among Burkitt lymphoma patients. Burkitt, Burkitt’s, African Lymphoma, Epstein–Barr, EBV, Human Herpesvirus 4, HHV4, HHV-4, and EB virus were used in the search utilizing a combination of Boolean logical operators (‘AND’ & ‘OR’) and the ‘Advanced’ and ‘Expert’ search options (Table S1). To ensure a thorough method, the references of the included papers were also examined. To organize and filter out duplicate studies, EndNote X9 software was used.

### 2.4. Study Selection

Two authors (M.J.A.-K. and N.H.I.) independently screened the research title and abstract, followed by the entire text, of all studies retrieved from the literature search to determine the matched studies to be included. Excluded studies include review articles, case studies, non-human studies, views, and viewpoints. Data from news accounts and press releases and information acquired from blogs and databases were not considered. With M.F.J., F.A.H, A.A.M.Y., A.T., and M.A.I, disagreements regarding inclusion were discussed and a consensus was reached.

### 2.5. Data Extraction

The data from the included studies were accessed independently by two authors (M.J.A.-K. and N.H.I.). Before the data extraction procedure, all non-English language studies were translated into English using Google Translate. The data extracted from each of the eligible studies was imported into a predetermined Excel spreadsheet. The following are the extracted data from the selected studies: author name, study type, country, number of BL patients, participants’ age, number of EBV positives in BL, sample type, and EBV detection method. Any discrepancies, or confusing or unfounded data were discussed among the authors in order to reach an agreement. If the problem remains, the corresponding or first author of each study was emailed for clarification.

### 2.6. Quality Assessment and Publication Bias

The quality of the included studies was assessed using Joanna Briggs Institute’s critical appraisal tools. The studies were defined as poor-quality (high risk of bias), moderate-quality (moderate risk of bias), or high-quality (low risk of bias) if the overall score was ≤49%, 50–69%, or ≥70%, respectively [51,52]. Egger’s test was used to verify the funnel plot’s asymmetry. To evaluate publication bias, a funnel plot was constructed to compare the prevalence estimate against the standard error.

### 2.7. Data Analyses

To address the inconsistency among the included studies, a tau-squared test was used to assess heterogeneity (I^2^), where *p* < 0.05 was regarded as statistically significant. A greater homogeneity was regarded as an I^2^ value close to zero, where I^2^ values between 25–50% indicated low heterogeneity, 51–75% indicated moderate heterogeneity, and >75% indicated significant heterogeneity. Based on the critical assessment tools, two authors M.J.A.-K. and N.H.I.) evaluated the quality of each of the included studies by using the critical assessment tools.

Sensitivity analyses and Galbraith plots were also used to assess the quality of the results and identify potential causes of heterogeneity, respectively. The following strategies were used to conduct sensitivity analyses: excluding small studies (*n* < 100); excluding low-quality studies (high risk of bias); excluding studies that did not disclose the prevalence of EBV in patients with BL; only considering cross-sectional studies; and excluding outlier studies. All analyses and plots were generated by using RevMan software (version 5.3.5), RStudio (version 1.1.463), and the metafor package (version 2.0-0) of R software (version 3.5.1) [53].

### 2.8. Subgroup and Sensitivity Analyses

For subgroup analysis, the prevalence of EBV in patients with BL was analyzed through four-time interval trends (1969–1982, 1983–1995, 1996–2008, and 2009–2021); methods of EBV detection (nucleic acid hybridization, polymerase chain reaction (PCR), immunofluorescence, in situ hybridization (ISH), ISH+PCR, and southern blot); and geographical locations (Sub-Saharan Africa, Northern Africa, Southern America, Southern Asia, Northern America, Europe, Eastern Asia, and South-eastern Asia). The studies were categorized based on the sociodemographic index (SDI). To measure social and economic development, the SDI, which ranges from zero to one, employs data on the world’s economies, educational systems, and fertility rates. The SDI is divided into five categories: high SDI (lower bound to upper bound: 0.805129 to 1), high–middle SDI (lower bound to upper bound: 0.689504 to 0.805129), middle SDI (lower bound to upper bound: 0.607679 to 0.689504), low–middle SDI (lower bound to upper bound: 0.454743 to 0.607679), and low SDI (lower bound to upper bound: 0 to 0.454743) [54]. To identify the source of heterogeneity and check the robustness of the results, sensitivity analyses were performed using the following strategies: (1) excluding small studies (<100); (2) excluding low-quality studies (high risk of bias); (3) considering only cross-sectional studies; (4) considering only case-control studies; (5) considering only cohort studies; (6) considering only studies where the age was less than 18 years old; and (7) excluding the outlier studies.

## 3. Results

### 3.1. Selection and Inclusion of Studies

**Figure 1:**
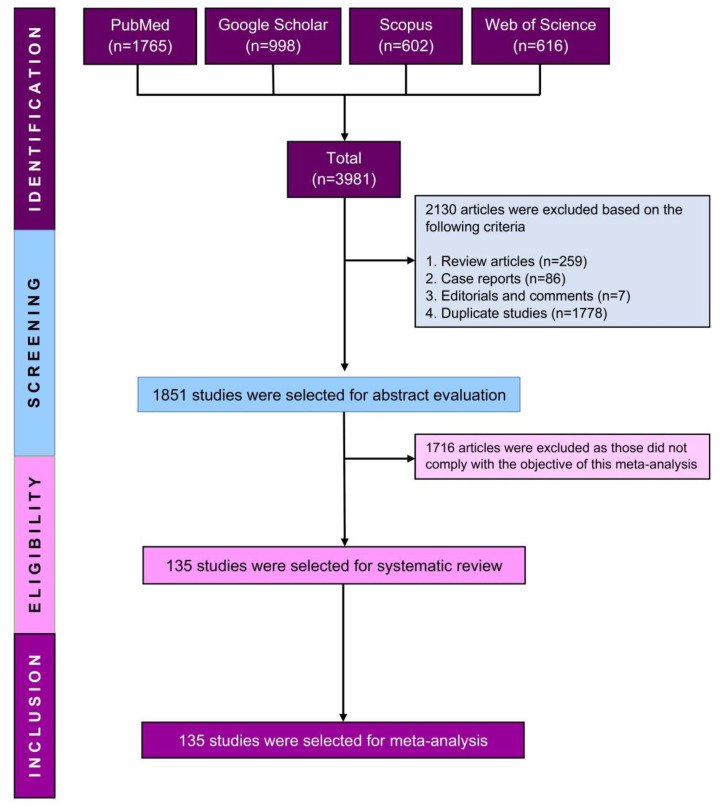
PRISMA flow diagram of study selection.

From the database search, 3981 studies qualified for initial screening, and then 2130 studies were excluded due to being duplicate studies (*n* = 1778), review articles (*n* = 259), case reports (*n* = 86), and non-human studies (*n* = 7). Therefore, 1851 studies were further assessed for eligibility by a detailed screening of the titles, abstracts, and full text. Finally, after excluding 1716 studies because they did not comply with the objective of this study, 135 studies were eligible to be included in this systematic review and meta-analysis, as illustrated in the PRISMA flow diagram (Figure 1).

### 3.2. Study Characteristics

Our literature search yielded 135 studies [37,38,39,40,41,42,43,44,45,46,47,48,49,50,51,52,53,54,55,56,57,58,59,60,61,62,63,64,65,66,67,68,69,70,71,72,73,74,75,76,77,78,79,80,81,82,83,84,85,86,87,88,89,90,91,92,93,94,95,96,97,98,99,100,101,102,103,104,105,106,107,108,109,110,111,112,113,114,115,116,117,118,119,120,121,122,123,124,125,126,127,128,129,130,131,132,133,134,135,136,137,138,139,140,141,142,143,144,145,146,147,148,149,150,151,152,153,154,155,156,157,158,159,160,161,162,163,164,165,166,167,168,169,170,171] published between 1969 and 2021, which examined the prevalence of EBV in patients with BL. Detailed characteristics and references of the included studies are presented in Table 2. Overall, this meta-analysis reports data from 4837 patients with BL lymphoma (34.7% female). The ages of these patients ranged from 2.1 ± 2.5 to 47.7 ± 31.8 years (mean ± SD; range, 0.7–98.0). The studies came from eight different regions, and these region groupings were based on the geographic regions defined under the Standard Country or Area Codes for Statistical Use (known as M49) of the United Nations Statistics Division [55]: region unspecified (*n* = 414), Sub-Saharan Africa (*n* = 2104) [56,57,58,59,60,61,62,63,64,65,66,67,68,69,70,71,72,73,74,75,76,77,78,79,80,81,82,83,84,85,86,87,88,89,90], Northern Africa (*n* = 507) [91,92,93,94,95,96,97,98,99,100,101,102,103,104], Southern America (*n* = 801) [105,106,107,108,109,110,111,112,113,114,115,116,117,118,119,120,121,122], Southern Asia (*n* = 37) [123,124,125], Northern America (*n* = 201) [126,127,128,129,130,131,132,133,134,135,136,137], Europe (*n* = 296) [138,139,140,141,142,143,144,145,146,147,148,149,150,151,152,153,154,155], Eastern Asia (*n* = 437) [156,157,158,159,160,161,162,163,164,165,166,167,168,169,170,171], and South-eastern Asia (*n* = 40) [172,173,174,175]. Multiple techniques were used to investigate the presence of EBV in patients with BL, including the use of single and combined methods of nucleic acid hybridization [61,63,73,79,80,81,133,134,160], polymerase chain reaction (PCR) [57,69,85,87,92,98,101,102,109,115,125,126,127,131,139,150,176], immunofluorescence [62,65,66,67,70,71,75,76,86,95,96,130,135,153,177,178,179,180], immunoassay [58,64,74,77,138,148,170], in situ hybridization (ISH) [60,68,72,78,82,83,88,89,90,91,93,97,99,104,105,106,108,112,116,117,118,119,120,121,124,128,129,132,140,141,142,143,144,146,147,149,151,152,154,155,156,157,158,159,161,162,163,164,166,168,169,171,173,174,181,182,183,184], Southern blot [111,136,145,165,167,185,186], and ISH+PCR [103,107,113,114,123,137,172,187,188]. The included studies were conducted between 1969 and 2021, and these studies were divided into four time groups with a fixed interval of 13 years for each: the groups of studies were from 1969 to 1982 [61,62,65,66,67,70,71,73,76,79,80,81,84,86,96,130,133,134,153,177,178], from 1983 to 1995 [57,63,78,83,93,94,95,100,101,109,111,112,122,127,128,129,136,139,141,143,144,145,146,148,151,154,155,156,165,167,170,176,179,180,183,185,186], from 1996 to 2008 [58,60,72,75,85,87,90,91,99,102,103,105,106,107,108,110,113,114,115,120,121,124,126,131,135,137,140,147,150,152,157,159,160,161,163,164,166,172,173,174,182,187,188], and from 2009 to 2021 [56,59,64,68,69,74,77,82,88,89,92,97,98,104,116,117,118,119,123,125,132,138,142,149,158,162,168,169,171,175,181,184,189,190].

Studies were categorized based on the socio-demographic index (SDI) into five categories: high SDI [126,127,128,129,130,131,132,133,134,135,136,137,139,142,143,144,145,146,147,148,151,153,154,155,157,158,159,161,163,165,166,167,170,171,176], high–middle SDI [91,94,95,96,99,100,102,103,107,109,112,116,138,140,141,149,150,152,173,174,175], middle SDI [92,93,97,104,105,106,108,110,113,114,115,117,118,119,120,121,122,123,156,160,162,164,168,169,172], low–middle SDI [59,62,68,74,82,83,87,125], and low SDI [56,57,58,60,61,64,66,67,69,70,71,72,75,76,77,78,79,80,81,84,85,86,88,89,90,98,101,124].

### 3.3. Outcomes

**Figure 2:**
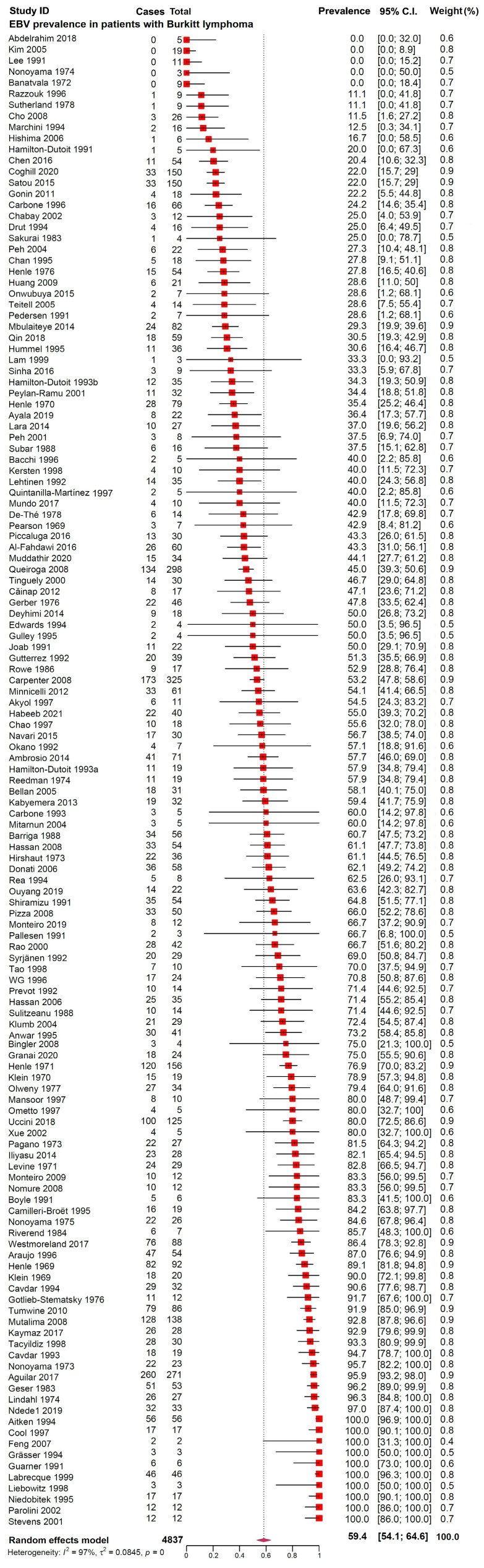
Forest plots presenting the prevalence of Epstein–Barr virus in patients with Burkitt lymphoma [55,56,57,58,59,60,61,62,63,64,65,66,67,68,69,70,71,72,73,74,75,76,77,78,79,80,81,82,83,84,85,86,87,88,89,90,91,92,93,94,95,96,97,98,99,100,101,102,103,104,105,106,107,108,109,110,111,112,113,114,115,116,117,118,119,120,121,122,123,124,125,126,127,128,129,130,131,132,133,134,135,136,137,138,139,140,141,142,143,144,145,146,147,148,149,150,151,152,153,154,155,156,157,158,159,160,161,162,163,164,165,166,167,168,169,170,171,172,173,174,175,176,177,178,179,180,181,182,183,184,185,186,187,188,189].

The pooled prevalence of EBV in patients with BL was 59.4% (95% CI, 54.1–64.6%, *n* = 4837), as illustrated in Figure 2.

### 3.4. Subgroup Analyses

**Figure 3:**
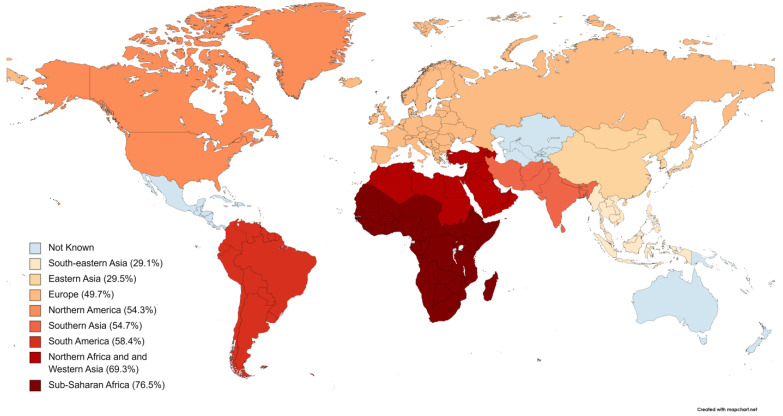
Global prevalence of Epstein–Barr virus in patients with Burkitt lymphoma.

Based on the subgroup analyses of the prevalence of EBV in patients with BL over four time intervals, we found a gradually decreasing prevalence of EBV in patients with BL, which was 64.2% (95% CI: 52.0 to 75.6; *p* < 0.01) from 1969 to 1982, then 60.9% (95% CI: 50.3 to 71.1; *p* < 0.01) from 1983 to 1995, then 60.7% from 1996 to 2008, and finally 54.0% (95% CI: 42.2 to 65.5; *p* < 0.01) that had a lower prevalence than the pooled prevalence within the period from 2009 to 2021 (Table 3 and Figure S1A–D). Furthermore, subgroup analyses based on the methods of EBV detection revealed a significantly increased prevalence when compared to the pooled prevalence in the nucleic acid hybridization at 81.7% (95% CI: 67.8 to 92.5; *p* < 0.01), 74.7% (95% CI: 60.0 to 87.1; *p* < 0.01) in the PCR method, and 60.0% (95% CI: 45.8 to 73.5; *p* < 0.01) in the immunofluorescence method. On the other hand, the prevalence in immunoassay, in situ hybridization (ISH), combined ISH with PCR, and Southern blot revealed a significantly lower prevalence: 54.7% (95% CI: 34.2 to 74.5; *p* < 0.01), 54.3% (95% CI: 46.3 to 62.1; *p* < 0.01), 53.2% (95% CI: 52.9 to 63.3; *p* = 0.01), and 47.1% (95% CI: 31.7 to 62.8; *p* < 0.01), respectively (Table 3 and Figure S1E–K). The subgroup analysis based on different geographical locations revealed a significantly increased prevalence when compared to the pooled prevalence only in Sub-Saharan Africa and Northern Africa, at 76.5% (95% CI: 67.0 to 84.9; *p* < 0.01) and 69.3% (95% CI: 58.1 to 79.4; *p* < 0.01), respectively (Figure 3). Southern America, Southern Asia, Northern America, Europe, Eastern Asia, and South-eastern Asia showed a decrease in prevalence when compared to the pooled prevalence at 58.4% (95% CI: 50.0 to 66.6; *p* < 0.01), 54.7% (95% CI: 30.5 to 77.9; *p* = 0.12), 54.3% (95% CI: 34.5 to 73.5; *p* < 0.01), 49.5% (95% CI: 36.9 to 62.5; *p* < 0.01), 29.5% (95% CI: 19.9 to 40.1; *p* < 0.01), and 29.1% (95% CI: 11.0 to 51.2; *p* = 0.15), respectively (Table 3 and Figure S1L–S). The subgroup analysis based on the socio-demographic index (SDI) revealed a significantly increased prevalence when compared to the pooled prevalence in both the middle and low SDI, at 60.1% (95% CI: 52.4 to 67.5; *p* < 0.01) and 82.7% (95% CI: 74.4 to 89.8; *p* = 0), respectively. On the other hand, countries with high SDI, high–middle SDI, and low–middle SDI showed a significant decrease in prevalence, at 43.0% (95% CI: 33.3 to 52.9; *p* = 0), 54.5% (95% CI: 40.0 to 68.6; *p* = 0), and 49.9% (95% CI: 31.4 to 68.5; *p* = 0), respectively (Table 3 and Figure S1T–X).

### 3.5. Quality Assessment

**Figure 4:**
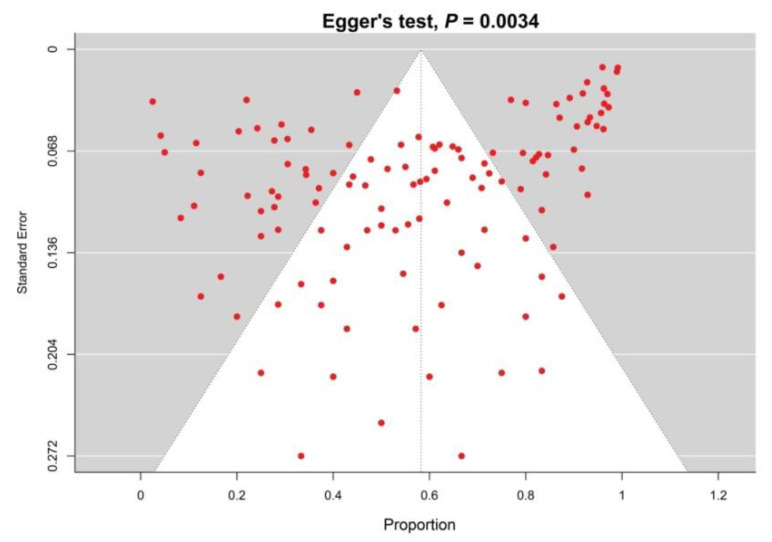
Funnel plots estimating the prevalence of EBV in patients with BL revealed significant publication bias [55,56,57,58,59,60,61,62,63,64,65,66,67,68,69,70,71,72,73,74,75,76,77,78,79,80,81,82,83,84,85,86,87,88,89,90,91,92,93,94,95,96,97,98,99,100,101,102,103,104,105,106,107,108,109,110,111,112,113,114,115,116,117,118,119,120,121,122,123,124,125,126,127,128,129,130,131,132,133,134,135,136,137,138,139,140,141,142,143,144,145,146,147,148,149,150,151,152,153,154,155,156,157,158,159,160,161,162,163,164,165,166,167,168,169,170,171,172,173,174,175,176,177,178,179,180,181,182,183,184,185,186,187,188,189].

In Tables S2–S4, the quality assessment of the included studies was presented in detail. Generally, of the included studies, 65.2%, 29.6%, and 5.2% were high-, moderate-, and low-quality studies, respectively. The funnel plot and Egger’s test results revealed evidence of a publication bias for the prevalence of EBV in BL (*p* = 0.0034) (Figure 4).

### 3.6. Heterogeneity and Sensitivity Analysis

In sensitivity analyses, the highest EBV prevalence in patients with BL was observed when considering only case-control studies (67.6%; 95% CI: 58.0 to 76.5) [56,58,59,61,62,65,66,67,70,71,74,75,76,78,85,86,90,92,93,94,95,96,97,98,100,103,105,108,114,115,117,122,130,152,153,171,178,179,182], followed by considering only studies where the age was less than 18 years old (64.9%; 95% CI: 55.4 to 74.0) [56,58,59,61,62,63,69,70,72,73,74,75,77,78,81,89,90,94,95,98,99,102,103,104,105,107,108,109,110,113,115,116,117,120,122,126,135,137,138,163,165,166,169,174], excluding small studies with less than 100 subjects (64.0%; 95% CI: 40.3 to 84.9) [56,58,59,67,75,104,121,171], and excluding outlier studies (61.0%; 95% CI: 55.8 to 66.1) [56,57,58,59,60,61,62,63,64,65,66,67,68,69,70,71,72,73,74,75,76,77,78,79,80,81,82,83,84,85,86,87,88,89,90,91,92,93,94,95,96,97,98,99,100,101,102,103,104,105,106,107,108,109,110,111,112,113,114,115,116,117,118,119,120,121,122,123,124,125,126,127,128,129,130,131,132,133,134,135,136,137,138,139,140,141,142,143,144,145,146,147,148,149,150,151,152,153,154,155,156,157,158,159,160,161,162,164,166,167,168,169,170,171,172,173,174,175,176,178,179,180,181,182,183,184,185,186,187,188,189,190]. In contrast, the lowest EBV prevalence in patients with BL was found when considering only cohort studies (48.4%; 95% CI: 35.9 to 61.1) [64,89,99,110,120,125,126,138,142,149,154,161,167,169,176,177,181], followed by considering only cross-sectional studies (54.4%; 95% CI: 50.1 to 64.6) [57,60,63,68,69,72,73,77,79,80,81,82,83,84,87,88,91,101,102,104,106,107,109,111,112,113,116,118,119,121,123,124,127,128,129,131,132,133,134,135,136,137,139,140,141,143,144,145,146,147,148,150,151,155,156,157,158,159,160,162,163,164,165,166,168,170,172,173,174,180,183,184,185,186,187,188,189,190], and excluding low- and moderate-quality studies (58.7%; 95% CI: 51.8 to 65.3) [56,57,58,59,61,62,63,68,69,72,73,75,77,80,81,82,83,84,88,89,91,92,94,95,96,97,98,102,103,104,106,107,109,112,113,116,117,118,119,120,121,122,123,124,128,129,131,132,134,135,136,138,139,140,141,146,147,148,150,151,154,155,156,157,158,159,160,162,163,164,165,166,168,170,172,173,174,175,178,180,183,184,185,186,187,188,189,190] (Table 4 and Figure S2A–G).

**Figure 5:**
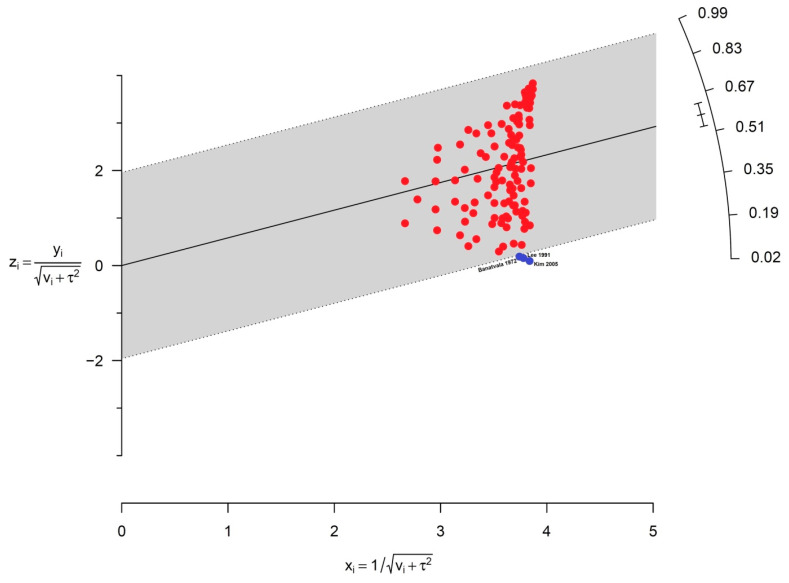
Galbraith plots show two outlier studies in estimating the prevalence of EBV in patients with BL [55,56,57,58,59,60,61,62,63,64,65,66,67,68,69,70,71,72,73,74,75,76,77,78,79,80,81,82,83,84,85,86,87,88,89,90,91,92,93,94,95,96,97,98,99,100,101,102,103,104,105,106,107,108,109,110,111,112,113,114,115,116,117,118,119,120,121,122,123,124,125,126,127,128,129,130,131,132,133,134,135,136,137,138,139,140,141,142,143,144,145,146,147,148,149,150,151,152,153,154,155,156,157,158,159,160,161,162,163,164,165,166,167,168,169,170,171,172,173,174,175,176,177,178,179,180,181,182,183,184,185,186,187,188,189].

As depicted in the Galbraith plot (Figure 5), three outlier studies in estimating the prevalence of EBV in patients with BL were determined. The results showed significant heterogeneity at 97%, *p* < 0.001.

## 4. Discussion

EBV was found to be associated with human cancer when it was discovered in BL. This was a result of BL cell isolation. EBV has been extensively characterized due to purported links to a variety of human diseases, including BL, HL, post-transplant and AIDS-related lymphomas, and nasopharyngeal carcinoma [7,191,192]. Our results revealed a high prevalence of EBV in patients with BL, at 59.4% in all BL patients worldwide. However, as shown in our study, the prevalence of EBV in patients with BL varies by region; we found the highest prevalence in Sub-Saharan Africa (76.5%) and Northern Africa (69.3%), while the prevalence in Southern America (58.4%), Southern Asia (54.7%), Northern America (54.3%), Europe (49.7%), Eastern Asia (29.5%), and South-eastern Asia (29.1%) were lower than the pooled prevalence. We can explain the variations in EBV prevalence among patients with BL worldwide, as more than 95% of people in the world acquire the Epstein–Barr virus, a herpes virus belonging to the gamma subfamily, within the first ten years of life. Primary exposure to infections occurs in childhood in Africa and other developing countries, probably as a result of different cultural norms compared to developed countries [115,193].

The Epstein–Barr virus infection persists asymptomatically for the entirety of the host’s life, maintaining the immune system and this deceptive virus constantly in balance. In our study, the incidence of BL was higher in children (≤18) at 64.9% compared to adults; this corresponds to many studies that report that BL is more common in children [194,195]. Our results revealed that the incidence of BL among males is much higher than in females (34.7%), which is commensurate with several studies that report that BL is more prevalent in males compared to females [104,120,123,195]. This result is in agreement with Yakimchuk et al., which reported that estrogen has an anti-proliferative effect on BL cells through estrogen receptor β (ERβ) signalling [196]. Our study revealed a significant publication bias for EBV prevalence in patients with BL, and that is in agreement with some studies exploring the prevalence of EBV in different diseases, such as multiple sclerosis (*p* < 0.05) [197] and breast cancer (*p* = 0.006) [198], while that is in disagreement with some studies such as for gastric carcinoma (*p =* 0.912) [199], Hodgkin’s lymphoma (*p* = 0.162) [200], and EBV-associated epithelial tumors (*p* = 0.23617) [201].

Interestingly, our study shows a significant decline in EBV prevalence over four time periods (13 years), with the prevalence decreasing from 64.2% in the period from 1969 to 1982, to 54% in the period from 2009 to 2021. This decrease in incidence could be attributed to the development and widespread use of EBV vaccines, as well as improved sanitation, living habits, and personal hygiene [202,203]. There are many methods used to detect EBV, but these methods are different depending on whether they are faster, are more sensitive, or provide more informative than previous assays [204]. Our study revealed that the most used method in EBV detection was the microscopic examination (in situ hybridization (ISH) in 59 studies and immunofluorescence in 18 studies) method followed by molecular methods (PCR in 17 studies, nucleic acid hybridization in nine studies, ISH+PCR in nine studies, and Southern blot in seven studies), and, finally, immunoassay methods in seven studies. This result confirms that ISH is the methodology of choice for the detection of EBV in tissue sections [205,206,207]. Our results revealed a higher prevalence of EBV in patients with BL in both low and middle SDI countries, at 82.7% and 60.1%, respectively. A study showed that the highest incidence and mortality burden occurred in EBV-attributed BL in low and low–middle SDI areas [208]. The reasons for the increases in the burden of malignancies related to EBV infection appear to be growing populations, an increase in life expectancy, and changing age structure [209].

## 5. Conclusions

In conclusion, based on the comprehensive systematic and meta-analysis of the available data on the prevalence of EBV in patients with BL until January 2021, the prevalence was 59.4% in all patients with BL. Due to factors such as cultural habits, personality hygiene, limited use of developed EBV vaccines, and malaria endemic areas, Sub-Saharan Africa (76.5%) and Northern Africa (69.3%) revealed the highest prevalence (hot spots) in comparison to the rest of the world. Countries with middle and low SDI have a higher prevalence of EBV in patients with BL. Despite the fact that the EBV prevalence in patients with BL has decreased significantly from 64.2% in 1969 to 1982 to 54% from 2009 to 2021, as well as there being a higher incidence in younger (≤18) patients than adults, EBV detection should be used as a routine test in hot spots as well as in all young people because it will help in predicting whether populations are at a high risk of increasing the number of BL cases corresponding to EBV infection.

## Figures and Tables

**Table 1:** Epidemiology features of EBV-associated neoplasia.

Gene Expressed	Product	EBV Latency Programs				
		Latency O	Latency I	Latency IIa	Latency IIb	Latency III
EBNA1	Protein	–	+	+	+	+
EBNA2	Protein	–	+	+	+	+
EBNA3	Protein	–	–	+	+	+
EBNA-LP	Protein	–	–	+	+	+
LMP1	Protein	–	–	+	+	+
LMP2	Protein	–	–	+	+	+
BARTs	Protein	–	–	+	+	+
EBERs	ncRNAs	+	+	+	+	+
**Associated Malignancies**		Memory B cells	- BL - EBVaGC	- NPC - T/NK LPD - EBV + DLBCL, NOS - cHL - NLPHL		- AIDS-associated B-cell lymphoma - DLBCL - PTLD

EBNA: EB viral nuclear antigen; EBNA-LP: EB viral nuclear antigen leader protein; LMP: latent membrane protein; BARTs: BamHI A rightward transcripts; EBERs: Epstein–Barr virus-encoded small RNAs; ncRNAs: non-coding RNAs; BL: Burkitt Lymphoma; EBVaGC: Epstein–Barr virus-associated gastric cancer; NPC: nasopharyngeal cancer; LPD: lymphoproliferative disorder; DLBCL, NOS: diffuse large B-cell lymphoma, not otherwise specified; cHL: classic Hodgkin’s Lymphoma; NLPHL: nodular lymphocyte-predominant Hodgkin’s lymphoma;PTLD: post-transplant lymphoproliferative disorder. “+” indicates the protein is expressed, while “−” indicates that the protein is not expressed.

**Table 2:** Major characteristics of the included studies.

No	Study ID	Type of Study	Country	Age (Mean ± SD/Range) (Years)	Type of Participants		Number of BL Patients (% Female)	EBV Positivity in BL # (%)	**Sample Type**	**Method of Detection of EBV**
					Age Group	# (%)				
1	Abdelrahim, 2018 [175]	Cross-sectional	Malaysia	22.2 ± 14.5 (32)	≤18	3 (60)	5 (60)	0 (0)	FFPE	ISH
					45 < Age > 18	2 (40)				
2	Aguilar, 2017 [56]	Case-control	Malawi	7.8 ± 2.9 (6.23)	≤18	271 (100)	271 (41)	260 (95.9)	Sera	qSAT
3	Aitken, 1994 [57]	Cross-sectional	New Guinea	NR	NR	NR	56 (NR)	56 (100)	FFPE	PCR
4	Akyol, 1997 [91]	Cross-sectional	Turkey	17 ± 19.1 (59)	≤18	7 (63.6)	11 (18.2)	6 (54.5)	FFPE	ISH
					45 < Age > 18	1 (9.1)				
					≥45	1 (9.1)				
					NR	2 (18.5)				
5	Al-Fahdawi, 2016 [92]	Case-control	Iraq	21.8 ± 11.7 (34)	≤18	19 (31.7)	60 (31.7)	26 (43.3)	FFPE	PCR
					45 < Age > 18	14 (68.3)				
6	Ambrosio, 2014 [181]	Cohort	Kenya, Spain, and Italy	NR	NR	NR	71 (NR)	41 (57.7)	FFPE	ISH
7	Anwar, 1995 [93]	Case-control	Egypt	9.1 ± 4.9 (20)	≤18	38 (92.7)	41 (41.5)	30 (73.2)	FFPE and fresh tumor biopsies	ISH
					45 < Age > 18	3 (7.3)				
8	Araujo, 1996 [105]	Case-control	Brazil	5.9 ± 3 (13)	≤18	54 (100)	54 (34.6)	47 (87)	FFPE	ISH
9	Ayala, 2019 [184]	Cross-sectional	Kenya and Italy	16.6 ± 13.2 (42)	≤18	15 (68.2)	22 (50)	8 (36.4)	FFPE	ISH
					45 < Age > 18	6 (27.3)				
					≥45	1 (4.5)				
10	Bacchi, 1996 [106]	Cross-sectional	Brazil	36 ± 8.5 (21)	45 < Age > 18	5 (100)	5 (20)	2 (40)	FFPE	ISH
11	Banatvala, 1972 [177]	Cohort	East Africa	NR	NR	NR	9 (NR)	0 (0)	Sera	Immunofluorescence
12	Barriga, 1988 [185]	Cross-sectional	Ghana and USA	NR	NR	NR	56 (NR)	34 (60.7)	Fresh tumor biopsies	Southern blotting
13	Bellan, 2005 [182]	Case-control	Kenya, France, and Italy	Range, 66	NR	NR	31 (NR)	18 (58.1)	FFPE	ISH
14	Bingler, 2008 [126]	Cohort	USA	2.1 ± 2.5 (5.1)	≤18	4 (100)	4 (50)	3 (75)	FFPE	PCR
15	Boyle, 1991 [176]	Cohort	Australia	NR	NR	NR	7 (NR)	5/6 (83.3)	FFPE and fresh tumor biopsies	PCR
16	Căinap, 2012 [138]	Cohort	Romania	NR	≤18	17 (100)	17 (NR)	8 (47.1)	Sera	Serological IgG VCA antibody
17	Camilleri-Broët, 1995 [139]	Cross-sectional	France	NR	NR	NR	19 (NR)	16 (84.2)	FFPE	PCR
18	Carbone, 1993 [141]	Cross-sectional	Italy	NR	NR	NR	5 (NR)	3 (60)	FFPE	ISH
19	Carbone, 1996 [140]	Cross-sectional	Italy	NR	NR	NR	66 (NR)	16 (24.2)	Fresh tumor biopsies	ISH
20	Carpenter, 2008 [58]	Case-control	Uganda	7 ± 3 (13.5)	≤18	325 (100)	325 (39.1)	173 (53.2)	Sera	Chemiluminescent immunoassay
21	Cavdar, 1993 [94]	Case-control	Turkey	5.5 (12)	≤18	72 (100)	72 (31.9)	18/19 (94.7)	Fresh tumor biopsies	Southern blotting + PCR
22	Cavdar, 1994 [95]	Case-control	Turkey	Median 5 (4.5)	≤18	81 (100)	81 (30)	29/32 (90.6)	Fresh tumor biopsies	Immunofluorescence
23	Chabay, 2002 [107]	Cross-sectional	Argentina	Range, 13.75	≤18	12 (100)	12 (NR)	3 (25)	FFPE	ISH + PCR
24	Chan, 1995 [156]	Cross-sectional	China	40.8 ± 24.9 (81)	≤18	10 (55.6)	18 (44.4)	5 (27.8)	FFPE	ISH
					45 < Age > 18	8 (44.4)				
25	Chao, 1997 [157]	Cross-sectional	Taiwan	33 ± 24.3 (72)	≤18	6 (33.3)	18 (33.3)	10 (55.6)	FFPE	ISH
					45 < Age > 18	7 (38.9)				
					≥45	5 (27.8)				
26	Chen, 2016 [158]	Cross-sectional	Taiwan	Median 27 (82)	≤18	21 (38.9)	54 (33)	11 (20.4)	FFPE	ISH
					NR	33 (66.1)				
27	Cho, 2008 [159]	Cross-sectional	South Korea	36 (NR)	NR	NR	26 (38.5)	3 (11.5)	FFPE	ISH
28	Coghill, 2020 [59]	Case-control	Ghana	8.3 (17)	≤18	150 (100)	150 (36.7)	33 (22.0)	Sera	Microarray
29	Cool, 1997 [60]	Cross-sectional	Kenya	Range, 56	NR	NR	21 (NR)	17/17 (100)	FFPE	ISH
30	De-Thé, 1978 [61]	Case-control	Uganda	6.6 ± 2.6 (9)	≤18	14 (100)	14 (35.7)	6 (42.9)	Fresh tumor biopsies	Nucleic acid hybridization
31	Deyhimi, 2014 [123]	Cross-sectional	Iran	21 (79)	NR	NR	18 (27.8)	9 (50)	FFPE	ISH + PCR
32	Donati, 2006 [110]	Cohort	Brazil	6.2 (NR)	≤18	58 (100)	58 (34.5)	36 (62.1)	Fresh tumor biopsies	HIS-FISH
33	Drut, 1994 [109]	Cross-sectional	Argentina	NR	≤18	16(100)	16 (50)	4 (25.0)	FFPE	PCR
34	Edwards, 1994 [127]	Cross-sectional	USA	NR	NR	NR	4 (NR)	2 (50)	Fresh tumor biopsies	PCR
35	Feng, 2007 [160]	Cross-sectional	China	Median 18.5 (31)	≤18	1 (50)	2 (50)	2 (100)	FFPE	Nucleic acid hybridization
					45 < Age > 18	1 (50)				
36	Gerber, 1976 [62]	Case-control	Ghana	Range, 12	≤18	46 (100)	46 (NR)	22 (47.8)	Sera	Immunofluorescence
37	Geser, 1983 [63]	Cross-sectional	Uganda and Sudan	7.5(15)	≤18	74 (100)	74 (80)	51/53 (96.2)	Fresh tumor biopsies	Nucleic acid hybridization
38	Gonin, 2011 [142]	Cohort	France	NR	NR	NR	18 (NR)	4 (22.2)	FFPE	ISH
39	Gotlieb- Stematsky, 1976 [96]	Case-control	Israel	7.2 ± 4.9 (16)	≤18	15 (93.8)	16 (37.5)	11/12 (91.7)	Sera	Immunofluorescence
					45 < Age > 18	1 (6.2)				
40	Granai, 2020 [64]	Cohort	Uganda	NR	NR	NR	24 (NR)	18 (75)	FFPE	IHC
41	Grässer, 1994 [143]	Cross-sectional	UK	NR	NR	NR	3 (NR)	3 (100)	FFPE and fresh tumor biopsies	ISH
42	Guarner, 1991 [128]	Cross-sectional	USA	36.7 ± 3.9 (10)	45 < Age > 18	6 (100)	6 (NR)	6 (100)	FFPE	ISH
43	Gulley, 1995 [129]	Cross-sectional	USA	35 (46)	NR	NR	4 (50)	2 (50)	FFPE	ISH
44	Gutterrez, 1992 [111]	Cross-sectional	South America (Brazil, Chile, and Argentina)	7.4 ± 5.1 (27)	≤18	37 (94.9)	39 (18)	20 (51.3)	Fresh tumor biopsies	Southern blotting
					45 < Age > 18	2 (5.1)				
45	Habeeb, 2021 [97]	Case-control	Syria	11.5 (56)	4 to 12	37 (92.5)	40 (27.5)	22 (55)	FFPE	ISH
					48-60	3 (7.5)				
46	Hamilton-Dutoit, 1991 [112]	Cross-sectional	Argentina	45 ± 18.5 (59)	45 < Age > 18	4 (57.1)	7 (14.3)	1/5 (20)	FFPE	ISH
					≥45	3 (42.9)				
47	Hamilton-Dutoit, 1993a [144]	Cross-sectional	Denmark	37.2 ± 13.1 (64)	≤18	1 (5.3)	19 (0)	11 (58)	Fresh tumor biopsies	Southern blotting
					45 < Age > 18	5 (26.3)				
					≥45	13 (68.4)				
48	Hamilton-Dutoit, 1993b [145]	Cross-sectional	Denmark	NR	NR	NR	35 (NR)	12 (34.3)	FFPE	ISH
49	Hassan, 2006 [114]	Case-control	Brazil	Range, 14	NR	NR	35 (NR)	25 (71.4)	FFPE	ISH+PCR
50	Hassan, 2008 [113]	Cross-sectional	Brazil	Median 5 (12)	≤18	54 (100)	54 (33.3)	33 (61.1)	FFPE and fresh tumor biopsies	ISH + PCR
51	Henle, 1969 [66]	Case-control	Kenya	NR	NR	NR	92 (NR)	82 (89.1)	Sera	Immunofluorescence
52	Henle, 1970 [76]	Case-control	Kenya	NR	NR	NR	79 (NR)	28 (35.4)	Fresh tumor biopsies	Immunofluorescence
53	Henle, 1971 [67]	Case-control	Kenya	NR	NR	NR	156 (NR)	120 (76.9)	Sera	Immunofluorescence
54	Henle, 1976 [65]	Case-control	Uganda and Ghana	NR	NR	NR	54 (NR)	15 (27.8)	Sera	Immunofluorescence
55	Hirshaut, 1973 [178]	Case-control	Uganda and USA	NR	NR	NR	36 (NR)	22 (61.1)	Sera	Immunofluorescence
56	Hishima, 2006 [161]	Cohort	Japan	34.7 (16)	45 < Age > 18	6 (100)	6 (0)	1 (16.7)	Fresh tumor biopsies	ISH
57	Huang, 2009 [162]	Cross-sectional	China	27.8 ± 20.6 (69)	≤18	7 (33.3)	21 (19.0)	6 (28.6)	FFPE	ISH
					45 < Age > 18	10 (47.6)				
					≥45	4 (19.1)				
58	Hummel, 1995 [146]	Cross-sectional	Germany	NR	NR	NR	36 (NR)	11 (30.6)	FFPE	ISH
59	Iliyasu, 2014 [68]	Cross-sectional	Nigeria	NR	NR	NR	28 (NR)	23 (82.1)	FFPE	ISH
60	Joab, 1991 [179]	Case-control	France & China	NR	NR	NR	22 (NR)	11 (50)	Sera	Immunofluorescence
61	Kabyemera, 2013 [98]	Case-control	Tanzania	Range, 14	≤18	32 (100)	32 (56.2)	19 (59.4)	Blood	PCR
62	Kaymaz, 2017 [69]	Cross-sectional	Kenya	Median 8.2 (12)	≤18	28 (100)	28 (29.0)	26 (92.9)	Fresh tumor biopsies	PCR
63	Kersten, 1998 [147]	Cross-sectional	Netherlands	NR	NR	NR	10 (NR)	4 (40)	FFPE	ISH
64	Kim, 2005 [163]	Cross-sectional	South Korea	Range, 17.2	≤18	19 (100)	19 (NR)	0 (0)	FFPE	ISH
65	Klein, 1969 [71]	Case-control	Kenya	NR	NR	NR	20 (NR)	18 (90)	Sera	Immunofluorescence
66	Klein, 1970 [70]	Case-control	Kenya	7.2 ± 3.0 (12)	≤18	19 (100)	19 (42.1)	15 (78.9)	Sera	Immunofluorescence
67	Klumb, 2004 [115]	Case-control	Brazil	Range, 8	≤18	37 (100)	37 (32.4)	21/29 (72.4)	FFPE and fresh tumor biopsies	PCR
68	Labrecque, 1999 [72]	Cross-sectional	Malawi	7.1 (10)	≤18	46 (100)	46 (39.1)	46 (100)	FFPE and fresh tumor biopsies	ISH
69	Lam, 1999 [164]	Cross-sectional	China	47.7 ± 31.8 (61)	≤18	2 (66.7)	3 (100)	1 (33.3)	FFPE	ISH
					≥45	1 (33.3)				
70	Lara, 2014 [116]	Cross-sectional	Argentina	Range, 15	≤18	27 (100)	27 (37.0)	10 (37.0)	FFPE	ISH
71	Lee, 1991 [165]	Cross-sectional	Taiwan	Range, 14	≤18	11 (100)	11 (NR)	0 (0)	FFPE	Southern blotting
72	Lehtinen, 1992 [183]	Cross-sectional	Finland and Tanzania	28.3 (65)	NR	NR	35 (42.9)	14 (40)	FFPE	ISH
73	Levine, 1971 [130]	Case-control	USA	13.7 ± 9.2 (40)	≤18	23 (79.3)	29 (41.4)	24 (82.8)	Sera	Immunofluorescence
					45 < Age > 18	6 (20.7)				
74	Liebowitz, 1998 [131]	Cross-sectional	USA	NR	NR	NR	3 (NR)	3 (100)	Fresh tumor biopsies	PCR
75	Lindahl, 1974 [73]	Cross-sectional	Africa	7.6 ± 2.9 (10)	≤18	27 (100)	27 (44.4)	26 (96.3)	FFPE and fresh tumor biopsies	Nucleic acid hybridization
76	Mansoor, 1997 [124]	Cross-sectional	Pakistan	10.7 ± 5.7 (18)	≤18	9 (90)	10 (30)	8 (80)	FFPE	ISH
					45 < Age > 18	1 (10)				
77	Marchini, 1994 [148]	Cross-sectional	Sweden	NR	NR	NR	16 (NR)	2 (12.5)	Sera	ELISA
78	Mbulaiteye, 2014 [132]	Cross-sectional	USA	NR	0–19	24 (26)	91 (13)	24/82 (29.3)	FFPE	ISH
					20–34	14 (15)				
					35–59	26 (29)				
					≥60	17 (19)				
					NR	10 (11)				
79	Minnicelli, 2012 [117]	Case-control	Brazil	Median 5 (12)	≤18	62 (100)	62 (30.6)	33/61 (54.1)	FFPE	ISH
80	Mitarnun, 2004 [172]	Cross-sectional	Thailand	35.6 (31)	45 < Age > 18	5 (100)	5 (40)	3 (60)	FFPE	ISH + PCR
81	Monteiro, 2009 [118]	Cross-sectional	Brazil	Range, 95	≤15	7/10 (70)	12 (33.3)	10 (83.3)	FFPE	ISH
					>15	3/10 (30)				
82	Monteiro, 2019 [119]	Cross-sectional	Brazil	23,8 (95)	NR	NR	12 (33.3)	8/12 (66.7)	FFPE	ISH
83	Muddathir, 2020 [74]	Case-control	Sudan	Range, 11	≤18	34 (100)	34 (38.2)	15 (44.1)	FFPE	IHC
84	Mundo, 2017 [149]	Cohort	Italy	14.2 (38)	NR	NR	10 (50)	4 (40)	FFPE	ISH
85	Mutalima, 2008 [75]	Case-control	Malawi	7.1 ± 2.6 (15)	≤18	148 (100)	148 (40)	128/138 (92.8)	Sera	Immunofluorescence
86	Navari, 2015 [189]	Cross-sectional	Italian and African	35 ± 22.8 (74.5)	≤18	8 (26.7)	30 (7/20 [35%])	17 (56.7)	FFPE	DASL
					45 < Age > 18	9 (30)				
					≥45	10 (33.3)				
					NR	3 (10)				
87	Ndede1, 2019 [77]	Cross-sectional	Kenya	NR	≤18	33 (100)	33 (21.2)	32 (97)	Sera + fresh tumor biopsies	ELISA + IHC
88	Niedobitek, 1995 [78]	Case-control	Uganda and Malawi	8.2 ± 3.9 (14)	≤18	17 (100)	17 (53)	17 (100)	FFPE	ISH
89	Nomure, 2008 [166]	Cross-sectional	Japan	6.2 ± 2.7 (10)	≤18	12 (100)	12 (25)	10 (83.3)	FFPE	ISH
90	Nonoyama, 1973 [79]	Cross-sectional	Kenya	NR	NR	NR	23 (NR)	22 (95.7)	Fresh tumor biopsies	Nucleic acid hybridization
91	Nonoyama, 1974 [133]	Cross-sectional	USA	NR	NR	NR	3 (NR)	0 (0)	Fresh tumor biopsies	Nucleic acid hybridization
92	Nonoyama, 1975 [80]	Cross-sectional	Kenya	NR	NR	NR	26 (NR)	22 (84.6)	Fresh tumor biopsies	Nucleic acid hybridization
93	Okano, 1992 [167]	Cohort	Japan	14.7 ± 12.7 (35)	≤18	6 (85.7)	7 (42.9)	4 (57.1)	Fresh tumor biopsies	Southern blotting
					45 < Age > 18	1 (14.3)				
94	Olweny, 1977 [81]	Cross-sectional	Uganda	7.2 (13)	≤18	34 (100)	34 (32.3)	27 (79.4)	Fresh tumor biopsies	Nucleic acid hybridization
95	Ometto, 1997 [150]	Cross-sectional	Italy	NR	NR	NR	5 (NR)	4 (80.0)	FFPE	PCR
96	Onwubuya, 2015 [82]	Cross-sectional	Nigeria	16.9 (50)	0–20	6 (85.7)	7 (28.6)	2 (28.6)	FFPE	ISH
					41–60	1 (14.3)				
97	Ouyang, 2019 [168]	Cross-sectional	China	NR	NR	NR	22 (NR)	14 (63.6)	FFPE	ISH
98	Pagano, 1973 [134]	Cross-sectional	USA	NR	NR	NR	27 (NR)	22 (81.5)	Fresh tumor biopsies	Nucleic acid hybridization
99	Pallesen, 1991 [151]	Cross-sectional	Denmark	39.3 ± 6.4 (12)	45 < Age > 18	8 (61.5)	3 (0)	2 (66.7)	Fresh tumor biopsies	ISH
100	Parolini, 2002 [152]	Case-control	Italy	NR	NR	NR	12 (0)	12 (100)	FFPE	ISH
101	Pearson, 1969 [153]	Case-control	Sweden	NR	NR	NR	7 (NR)	3 (37.5)	Sera	Immunofluorescence
102	Pedersen, 1991 [154]	Cohort	Denmark	NR	NR	NR	12 (NR)	2/7 (28.6)	FFPE	ISH
103	Peh, 2001 [173]	Cross-sectional	Malaysia	NR	NR	NR	8 (0)	3 (37.5)	FFPE	ISH
104	Peh, 2004 [174]	Cross-sectional	Malaysia	Range, 15	≤18	22 (100)	22 (22.7)	6 (27.3)	FFPE	ISH
105	Peylan-Ramu, 2001 [99]	Cohort	Israel	Median, 5	≤18	32 (100)	32 (25)	11 (34.4)	FFPE	ISH
106	Piccaluga, 2016 [190]	Cross-sectional	Italy and Africa	NR	NR	NR	30 (NR)	13 (43.3)	FFPE	DASL
107	Pizza, 2008 [120]	Cohort	Brazil	6±2.7 (13)	≤18	53 (100)	53 (24.5)	33/50 (66.0)	FFPE	ISH
108	Prevot, 1992 [83]	Cross-sectional	Cameroon, Gabon	NR	NR	NR	14 (NR)	10 (83.3)	FFPE	ISH
109	Qin, 2018 [169]	Cohort	China	NR	≤18	105 (100)	105 (15.2)	18/59 (30.5)	Fresh tumor biopsies	ISH
110	Queiroga, 2008 [121]	Cross-sectional	Brazil	23.1 (93)	≤16	149 (47.9)	311 (28.9)	134/298 (45)	FFPE	ISH
					>16	143 (46)				
					NR	19 (6.1)				
111	Quintanilla-Martínez, 1997 [187]	Cross-sectional	Mexico and European	NR	NR	NR	5 (NR)	2 (40)	FFPE	ISH + PCR
112	Rao, 2000 [188]	Cross-sectional	Southern India and Argentina	7.4 ± 5.1 (23)	≤18	39 (92.9)	42 (33.3)	28 (66.7)	FFPE	ISH + PCR
					45 < Age > 18	2 (4.7)				
					NR	1 (2.4)				
113	Razzouk, 1996 [135]	Cross-sectional	USA	Range, 13	≤18	9 (100)	9 (33.3)	1 (11.1)	Fresh tumor biopsies	Immunofluorescence
114	Rea, 1994 [155]	Cross-sectional	France	NR	NR	NR	9 (NR)	5/8 (62.5)	FFPE	ISH
115	Reedman, 1974 [84]	Cross-sectional	Kenya	8.7 ± 5.3 (19)	≤18	16 (84.2)	19 (NR)	11/19 (57.9)	Fresh tumor biopsies	CF
					45 < Age > 18	2 (10.5)				
					NR	1 (5.3)				
116	Riverend, 1984 [122]	Case-control	Cuba	7.6 ± 3.3 (9)	≤18	7 (100)	7 (42.9)	6 (85.7)	FFPE	CF
117	Rowe, 1986 [180]	Cross-sectional	France, Algeria, La Rcunion, and England	12.1±13.4 (51)	≤18	14 (82.3)	17 (29.4)	9 (53)	Fresh tumor biopsies	Immunofluorescence
					45 < Age > 18	2 (11.8)				
					≥45	1 (5.9)				
118	Sakurai, 1983 [170]	Cross-sectional	Japan	15.8 ± 16.7 (41)	≤18	4 (80)	5 (40)	1/4 (25)	Fresh tumor biopsies	ELISA
					≥45	1 (20)				
119	Satou, 2015 [171]	Case-control	Japan	Range, 85	NR	NR	150 (20.7)	33 (22)	FFPE	ISH
120	Shiramizu, 1991 [186]	Cross-sectional	Ghana & USA	NR	NR	NR	54 (NR)	35 (64)	Fresh tumor biopsies	Southern blotting
121	Sinha, 2016 [125]	Cohort	India	NR	≤18	7 (77.7)	9 (NR)	3 (33.3)	Plasma	PCR
					NR	3 (33.3)				
122	Stevens, 2001 [85]	Case-control	Malawi	NR	NR	NR	12 (NR)	12 (100)	Blood	PCR
123	Subar, 1988 [136]	Cross-sectional	USA	NR	NR	NR	16 (NR)	6 (37.5)	Fresh tumor biopsies	Southern blotting
124	Sulitzeanu, 1988 [100]	Case-control	Israel	NR	NR	NR	14 (NR)	10 (71.4)	sera	LMI
125	Sutherland, 1978 [86]	Case-control	Uganda	NR	NR	NR	9 (NR)	1 (11.1)	Fresh tumor biopsies	Immunofluorescence
126	Syrjänen, 1992 [101]	Cross-sectional	Tanzania	Range, 15	NR	NR	29 (14/27 [51.9%])	20 (69)	FFPE	PCR
127	Tacyildiz, 1998 [102]	Cross-sectional	Turkey	5.9 (NR)	≤18	30 (100)	30 (NR)	28 (93.3)	FFPE	PCR
128	Tao, 1998 [87]	Cross-sectional	Ghana	NR	NR	NR	10 (NR)	7 (70)	Fresh tumor biopsies	PCR
129	Teitell, 2005 [137]	Cross-sectional	USA	8.9 ± 4.6 (14)	≤18	14 (100)	14 (14.3)	4 (28.6)	FFPE	ISH + PCR
130	Tinguely, 2000 [103]	Case-control	Turkey	4.8 (9.5)	≤18	30 (100)	30 (NR)	14 (46.7)	FFPE	ISH+PCR
131	Tumwine, 2010 [88]	Cross-sectional	Uganda	NR	NR	NR	86 (NR)	79(91.9)	FFPE	ISH
132	Uccini, 2018 [104]	Cross-sectional	Iraq	5.9 ± 3.1	≤18	125 (100)	125 (21.1)	100 (80)	FFPE	ISH
133	Westmoreland, 2017 [89]	Cohort	Malawi	9.3±3.8	≤18	88 (100)	88 (34.1)	76 (86.4)	Fresh tumor biopsies and sera	ISH
134	WG, 1996 [108]	Case-control	Brazil	Median, 6	≤18	13/24 (54.1)	24 (8/15 [53.3%])	17 (70.8)	FFPE	ISH
135	Xue, 2002 [90]	Case-control	Malawi	7 ± 2.4 (6)	≤18	7 (100)	7 (57.1)	4/5 (80)	Fresh tumor biopsies	ISH

NR: not reported; #: number of cases; FFPE: formalin-fixed paraffin-embedded; ISH: in situ hybridization; qSAT: quantitative suspension array technology; PCR: polymerase chain reaction; HIS-FISH: histology fluorescence in situ hybridization; IHC: immunohistochemistry; ELISA: enzyme-linked immunosorbent assay; DASL: cDNA-mediated annealing, selection, extension, and ligation; CF: complement fixation; and LMI: leukocyte migration inhibition.

**Table 3:** Subgroup analysis of prevalence of EBV in patients with BL.

Subgroups	Prevalence of EBV [95% CI]	Studies Number	Positive for EBV	Heterogeneity	
				I^2^, %	*p* Value
Time Interval Trend					
From 1969 to 1982	64.2 [52.0–75.6]	21	497	95.0	<0.01
From 1983 to 1995	60.9 [50.3–71.1]	37	473	95.0	<0.01
From 1996 to 2008	60.7 [51.7–69.3]	43	939	97.0	<0.01
From 2009 to 2021	54.0 [42.2–65.5]	34	1005	98.0	<0.01
Methods of EBV detection					
Nucleic acid hybridization	81.7 [67.8–92.5]	9	178	86.0	<0.01
Polymerase chain reaction (PCR)	74.7 [60.0–87.1]	17	255	91.0	<0.01
Immunofluorescence	60.0 [45.8–73.5]	18	539	96.0	<0.01
Immunoassay	54.7 [34.2–74.5]	7	453	90.0	<0.01
In situ hybridization (ISH)	54.3 [46.3–62.1]	59	1058	97.0	<0.01
ISH+PCR	53.2 [52.9–63.3]	9	121	60.0	0.01
Southern blot	47.1 [31.7–62.8]	7	110	92.0	<0.01
Geographical location					
Sub-Saharan Africa	76.5 [67.0–84.9]	35	1500	77.0	<0.01
Northern Africa	69.3 [58.1–79.4]	14	344	89.0	<0.01
Southern America	58.4 [50.0–66.6]	18	443	84.0	<0.01
Southern Asia	54.7 [30.5–77.9]	3	20	66.0	0.05
Northern America	54.3 [34.5–73.5]	12	97	84.0	<0.01
Europe	49.7 [36.9–62.5]	18	122	91.0	<0.01
Eastern Asia	29.5 [19.9–40.1]	16	119	86.0	<0.01
South-eastern Asia	29.1 [11.0–51.2]	4	12	62.0	0.05
Socio-demographic Index					
High SDI	43.0 [33.3–52.9]	35	250	83.0	0
High–middle SDI	54.5 [40.0–68.6]	21	201	87.0	0
Middle SDI	60.1 [52.4–67.5]	25	641	82.0	<0.01
Low–middle SDI	49.9 [31.4–68.5]	8	115	87.0	0
Low SDI	82.7 [74.4–89.8]	28	1343	94.0	0

BL: Burkitt lymphoma; EBV: Epstein-Barr virus; CI: Confidence interval; SCI: socio-demographic index.

**Table 4:** Sensitivity analyses.

Strategies of Sensitivity Analyses	Prevalence [95% CIs] (%)	Difference of Pooled Prevalence Compared to the Main Result	Number of Studies Analyzed	Total Number of Subjects	Heterogeneity	
					I^2^, %	*p* Value
Excluding small studies (<100)	64.0 [40.3–84.9]	4.6% higher	8	1613	99%	<0.001
Excluding low- and moderate-quality studies	58.7 [51.8–65.3]	0.7 lower	88	3383	93%	<0.01
Considering only cross-sectional studies	54.4 [50.1–64.6]	5% lower	79	2114	91%	<0.01
Considering only case-control studies	67.6 [58.0–76.5]	8.2% higher	39	2218	97%	<0.01
Considering only cohort studies	48.4 [35.9–61.1]	11% lower	17	475	85%	<0.01
Considering only studies where the age was less than 18 years old	64.9 [55.4–74.0]	5.5% higher	44	2187	95%	<0.01
Excluding outlier studies	61.0 [55.8–66.1]	1.6% higher	132	4798	92%	<0.01

CIs: confidence intervals.

## Data Availability

The data are contained within the article or Supplementary Material.

## Supplementary Materials

The following supporting information can be downloaded at: https://www.mdpi.com/article/10.3390/diagnostics13122068/s1, Figure S1: Subgroup analyses estimating the prevalence of EBV at (A–D) different times, using (E–K) different EBV detection methods, in (L–S) different regions, and based on the (T–X) socio-demographic index (SDI); Figure S2: Sensitivity analyses (A) excluding small studies, (B) excluding low- and moderate-quality studies, (C) considering only cross-sectional studies, (D) considering only case-control studies, (E) considering only cohort, (F) considering only studies where the age was less than 18 years old, and (G) excluding outlier studies estimating the prevalence of EBV in patients with BL; Table S1: Search strategies; Table S2: Quality assessment of the included cross-sectional studies; Table S3: Quality assessment of the included case-control studies; Table S4: Quality assessment of the included cohort studies.

## References

[1] Bauer M., Jasinski-Bergner S., Mandelboim O., Wickenhauser C., Seliger B. (2021). Epstein-Barr Virus-Associated Malignancies and Immune Escape: The Role of the Tumor Microenvironment and Tumor Cell Evasion Strategies. Cancers.

[2] Machon C., Fabrega-Ferrer M., Zhou D., Cuervo A., Carrascosa J.L., Stuart D.I., Coll M. (2019). Atomic structure of the Epstein-Barr virus portal. Nat. Commun..

[3] Zanella L., Reyes M.E., Riquelme I., Abanto M., Leon D., Viscarra T., Ili C., Brebi P. (2021). Genetic Patterns Found in the Nuclear Localization Signals (NLSs) Associated with EBV-1 and EBV-2 Provide New Insights into Their Contribution to Different Cell-Type Specificities. Cancers.

[4] Huang J., Zhang L., Chen J., Wan D., Zhou L., Zheng S., Qiao Y. (2021). The Landscape of Immune Cells Indicates Prognosis and Applicability of Checkpoint Therapy in Hepatocellular Carcinoma. Front. Oncol..

[5] Shannon-Lowe C., Rickinson A. (2019). The Global Landscape of EBV-Associated Tumors. Front. Oncol..

[6] Dunmire S.K., Verghese P.S., Balfour H.H. (2018). Primary Epstein-Barr virus infection. J. Clin. Virol..

[7] Ayee R., Ofori M.E.O., Wright E., Quaye O. (2020). Epstein Barr Virus Associated Lymphomas and Epithelia Cancers in Humans. J. Cancer.

[8] Damania B., Munz C. (2019). Immunodeficiencies that predispose to pathologies by human oncogenic gamma-herpesviruses. FEMS Microbiol. Rev..

[9] Morris M.A. (2019). Cancer-Associated Fibroblasts in Undifferentiated Nasopharyngeal Carcinoma: A Putative Role for the EBV-Encoded Oncoprotein, LMP1. Pathogens.

[10] Rigaud S., Fondaneche M.C., Lambert N., Pasquier B., Mateo V., Soulas P., Galicier L., Le Deist F., Rieux-Laucat F., Revy P. (2006). XIAP deficiency in humans causes an X-linked lymphoproliferative syndrome. Nature.

[11] Münz C., Moormann A. (2008). Immune escape by Epstein–Barr virus associated malignancies. Semin. Cancer Biol..

[12] Cohen J.I. (2000). Epstein-Barr virus infection. N. Engl. J. Med..

[13] Magrath I. (2012). Epidemiology: Clues to the pathogenesis of Burkitt lymphoma. Br. J. Haematol..

[14] Basso K., Dalla-Favera R. (2015). Germinal centres and B cell lymphomagenesis. Nat. Rev. Immunol..

[15] O’Malley D.P., Auerbach A., Weiss L.M. (2015). Practical Applications in Immunohistochemistry: Evaluation of Diffuse Large B-Cell Lymphoma and Related Large B-Cell Lymphomas. Arch. Pathol. Lab. Med..

[16] Nakamura N., Nakamine H., Tamaru J., Nakamura S., Yoshino T., Ohshima K., Abe M. (2002). The distinction between Burkitt lymphoma and diffuse large B-Cell lymphoma with c-myc rearrangement. Mod. Pathol..

[17] Chuang S.S., Ye H., Du M.Q., Lu C.L., Dogan A., Hsieh P.P., Huang W.T., Jung Y.C. (2007). Histopathology and immunohistochemistry in distinguishing Burkitt lymphoma from diffuse large B-cell lymphoma with very high proliferation index and with or without a starry-sky pattern: A comparative study with EBER and FISH. Am. J. Clin. Pathol..

[18] Sapkota S., Shaikh H. (2023). Non-Hodgkin Lymphoma.

[19] Burkitt D.P.J.C. (1991). Malignant lymphoma in African children. Cancer.

[20] O’Conor G.T. (1961). Malignant lymphoma in African children. II. A pathological entity. Cancer.

[21] Swerdlow S.H., Campo E., Pileri S.A., Harris N.L., Stein H., Siebert R., Advani R., Ghielmini M., Salles G.A., Zelenetz A.D. (2016). The 2016 revision of the World Health Organization classification of lymphoid neoplasms. Blood.

[22] Hammerl L., Colombet M., Rochford R., Ogwang D.M., Parkin D.M. (2019). The burden of Burkitt lymphoma in Africa. Infect. Agents Cancer.

[23] Gastwirt J.P., Roschewski M. (2018). Management of adults with Burkitt lymphoma. Clin. Adv. Hematol. Oncol..

[24] Parroche P., Lauw F.N., Goutagny N., Latz E., Monks B.G., Visintin A., Halmen K.A., Lamphier M., Olivier M., Bartholomeu D.C. (2007). Malaria hemozoin is immunologically inert but radically enhances innate responses by presenting malaria DNA to Toll-like receptor 9. Proc. Natl. Acad. Sci. USA.

[25] Molyneux E.M., Rochford R., Griffin B., Newton R., Jackson G., Menon G., Harrison C.J., Israels T., Bailey S. (2012). Burkitt’s lymphoma. Lancet.

[26] Crombie J.L., LaCasce A.S. (2019). Epstein Barr Virus Associated B-Cell Lymphomas and Iatrogenic Lymphoproliferative Disorders. Front. Oncol..

[27] Teras L.R., DeSantis C.E., Cerhan J.R., Morton L.M., Jemal A., Flowers C.R. (2016). 2016 US lymphoid malignancy statistics by World Health Organization subtypes. CA Cancer J. Clin..

[28] Rochford R. (2021). Reframing Burkitt lymphoma: Virology not epidemiology defines clinical variants. Ann. Lymphoma.

[29] Aloisi F., Giovannoni G., Salvetti M. (2023). Epstein-Barr virus as a cause of multiple sclerosis: Opportunities for prevention and therapy. Lancet Neurol..

[30] Soldan S.S., Lieberman P.M. (2023). Epstein-Barr virus and multiple sclerosis. Nat. Rev. Microbiol..

[31] Hutcheson R.L., Chakravorty A., Sugden B. (2020). Burkitt Lymphomas Evolve to Escape Dependencies on Epstein-Barr Virus. Front. Cell. Infect. Microbiol..

[32] Kimura H., Kwong Y.L. (2019). EBV Viral Loads in Diagnosis, Monitoring, and Response Assessment. Front. Oncol..

[33] Calderwood M.A., Venkatesan K., Xing L., Chase M.R., Vazquez A., Holthaus A.M., Ewence A.E., Li N., Hirozane-Kishikawa T., Hill D.E. (2007). Epstein-Barr virus and virus human protein interaction maps. Proc. Natl. Acad. Sci. USA.

[34] Dugan J.P., Coleman C.B., Haverkos B. (2019). Opportunities to Target the Life Cycle of Epstein-Barr Virus (EBV) in EBV-Associated Lymphoproliferative Disorders. Front. Oncol..

[35] Welch J.J.G., Schwartz C.L., Higman M., Chen L., Buxton A., Kanakry J.A., Kahwash S.B., Hutchison R.E., Friedman D.L., Ambinder R.F. (2017). Epstein-Barr virus DNA in serum as an early prognostic marker in children and adolescents with Hodgkin lymphoma. Blood Adv..

[36] Haralambieva E., Banham A.H., Bastard C., Delsol G., Gaulard P., Ott G., Pileri S., Fletcher J.A., Mason D.Y. (2003). Detection by the fluorescence in situ hybridization technique of MYC translocations in paraffin-embedded lymphoma biopsy samples. Br. J. Haematol..

[37] Hecht J.L., Aster J.C. (2000). Molecular biology of Burkitt’s lymphoma. J. Clin. Oncol..

[38] Ryan J.L., Fan H., Swinnen L.J., Schichman S.A., Raab-Traub N., Covington M., Elmore S., Gulley M.L. (2004). Epstein-Barr Virus (EBV) DNA in plasma is not encapsidated in patients with EBV-related malignancies. Diagn. Mol. Pathol..

[39] Machado A.S., Da Silva Robaina M.C., Magalhaes De Rezende L.M., Apa A.G., Amoedo N.D., Bacchi C.E., Klumb C.E. (2010). Circulating cell-free and Epstein-Barr virus DNA in pediatric B-non-Hodgkin lymphomas. Leuk. Lymphoma.

[40] Kanakry J., Ambinder R. (2015). The Biology and Clinical Utility of EBV Monitoring in Blood. Curr. Top. Microbiol. Immunol..

[41] Kanapuru B.J.C.O. (2013). Plasma EBV levels as a prognostic marker in patients with advanced Hodgkin lymphoma. Clin. Oncol..

[42] Sinha M., Rao C.R., Shafiulla M., Shankaranand B., Viveka B.K., Lakshmaiah K.C., Jacob L.A., Babu G.K., Jayshree R.S. (2016). Plasma Epstein Barr viral load in adult-onset Hodgkin lymphoma in South India. Hematol. Oncol. Stem Cell Ther..

[43] Fung S.Y., Lam J.W., Chan K.C. (2016). Clinical utility of circulating Epstein-Barr virus DNA analysis for the management of nasopharyngeal carcinoma. Chin. Clin. Oncol..

[44] Chai S.J., Pua K.C., Saleh A., Yap Y.Y., Lim P.V., Subramaniam S.K., Lum C.L., Krishnan G., Mahiyuddin W.R., Malaysian N.P.C.S.G. (2012). Clinical significance of plasma Epstein-Barr Virus DNA loads in a large cohort of Malaysian patients with nasopharyngeal carcinoma. J. Clin. Virol..

[45] AbuSalah M.A.H., Gan S.H., Al-Hatamleh M.A.I., Irekeola A.A., Shueb R.H., Yean Yean C. (2020). Recent Advances in Diagnostic Approaches for Epstein-Barr Virus. Pathogens.

[46] Jeong Y., Cho C.E., Kim J.E., Lee J., Kim N., Jung W.Y., Sung J., Kim J.H., Lee Y.J., Jung J. (2022). Deep learning model to predict Epstein-Barr virus associated gastric cancer in histology. Sci. Rep..

[47] Muti H.S., Heij L.R., Keller G., Kohlruss M., Langer R., Dislich B., Cheong J.H., Kim Y.W., Kim H., Kook M.C. (2021). Development and validation of deep learning classifiers to detect Epstein-Barr virus and microsatellite instability status in gastric cancer: A retrospective multicentre cohort study. Lancet Digit. Health.

[48] Carreras J., Roncador G., Hamoudi R. (2022). Artificial Intelligence Predicted Overall Survival and Classified Mature B-Cell Neoplasms Based on Immuno-Oncology and Immune Checkpoint Panels. Cancers.

[49] Page M.J., McKenzie J.E., Bossuyt P.M., Boutron I., Hoffmann T.C., Mulrow C.D., Shamseer L., Tetzlaff J.M., Akl E.A., Brennan S.E. (2021). The PRISMA 2020 statement: An updated guideline for reporting systematic reviews. Int. J. Surg..

[50] Stroup D.F., Berlin J.A., Morton S.C., Olkin I., Williamson G.D., Rennie D., Moher D., Becker B.J., Sipe T.A., Thacker S.B. (2000). Meta-analysis of observational studies in epidemiology: A proposal for reporting. Meta-analysis Of Observational Studies in Epidemiology (MOOSE) group. JAMA.

[51] Seak Y.S., Nor J., Tuan Kamauzaman T.H., Arithra A., Islam M.A. (2021). Efficacy and Safety of Intranasal Ketamine for Acute Pain Management in the Emergency Setting: A Systematic Review and Meta-Analysis. J. Clin. Med..

[52] Hasim N., Bakar M.A.A., Islam M.A. (2021). Efficacy and Safety of Isotonic and Hypotonic Intravenous Maintenance Fluids in Hospitalised Children: A Systematic Review and Meta-Analysis of Randomised Controlled Trials. Children.

[53] Viechtbauer W. (2010). Conducting Meta-Analyses inRwith themetaforPackage. J. Stat. Softw..

[54] Global Burden of Disease Collaborative Network (2020). Global Burden of Disease Study 2019 (GBD 2019) Socio-Demographic Index (SDI) 1950–2019.

[55] Standard Country and Area Codes Classifications (M49). https://unstats.un.org/unsD/methods/m49/m49chang.htm.

[56] Aguilar R., Casabonne D., O’Callaghan-Gordo C., Vidal M., Campo J.J., Mutalima N., Angov E., Dutta S., Gaur D., Chitnis C.E. (2017). Assessment of the Combined Effect of Epstein-Barr Virus and Plasmodium falciparum Infections on Endemic Burkitt Lymphoma Using a Multiplex Serological Approach. Front. Immunol..

[57] Aitken C., Sengupta S.K., Aedes C., Moss D.J., Sculley T.B. (1994). Heterogeneity within the Epstein-Barr virus nuclear antigen 2 gene in different strains of Epstein-Barr virus. J. Gen. Virol..

[58] Carpenter L.M., Newton R., Casabonne D., Ziegler J., Mbulaiteye S., Mbidde E., Wabinga H., Jaffe H., Beral V. (2008). Antibodies against malaria and Epstein-Barr virus in childhood Burkitt lymphoma: A case-control study in Uganda. Int. J. Cancer.

[59] Coghill A.E., Proietti C., Liu Z., Krause L., Bethony J., Prokunina-Olsson L., Obajemu A., Nkrumah F., Biggar R.J., Bhatia K. (2020). The Association between the Comprehensive Epstein-Barr Virus Serologic Profile and Endemic Burkitt Lymphoma. Cancer Epidemiol. Biomark. Prev..

[60] Cool C.D., Bitter M.A. (1997). The malignant lymphomas of Kenya: Morphology, immunophenotype, and frequency of Epstein-Barr virus in 73 cases. Hum. Pathol..

[61] de-The G., Geser A., Day N.E., Tukei P.M., Williams E.H., Beri D.P., Smith P.G., Dean A.G., Bronkamm G.W., Feorino P. (1978). Epidemiological evidence for causal relationship between Epstein-Barr virus and Burkitt’s lymphoma from Ugandan prospective study. Nature.

[62] Gerber P., Nkrumah F.K., Pritchett R., Kieff E. (1976). Comparative studies of Epstein-Barr virus strains from Ghana and the United States. Int. J. Cancer.

[63] Geser A., Lenoir G.M., Anvret M., Bornkamm G., Klein G., Williams E.H., Wright D.H., De-The G. (1983). Epstein-Barr virus markers in a series of Burkitt’s lymphomas from the West Nile District, Uganda. Eur. J. Cancer Clin. Oncol..

[64] Granai M., Mundo L., Akarca A.U., Siciliano M.C., Rizvi H., Mancini V., Onyango N., Nyagol J., Abinya N.O., Maha I. (2020). Immune landscape in Burkitt lymphoma reveals M2-macrophage polarization and correlation between PD-L1 expression and non-canonical EBV latency program. Infect. Agents Cancer.

[65] Henle G., Henle W. (1976). Epstein-Barr virus-specific IgA serum antibodies as an outstanding feature of nasopharyngeal carcinoma. Int. J. Cancer.

[66] Henle G., Henle W., Clifford P., Diehl V., Kafuko G.W., Kirya B.G., Klein G., Morrow R.H., Munube G.M., Pike P. (1969). Antibodies to Epstein-Barr virus in Burkitt’s lymphoma and control groups. J. Natl. Cancer Inst..

[67] Henle G., Henle W., Klein G., Gunven P., Clifford P., Morrow R.H., Ziegler J.L. (1971). Antibodies to early Epstein-Barr virus-induced antigens in Burkitt’s lymphoma. J. Natl. Cancer Inst..

[68] Iliyasu Y., Ayers L.W., Liman A.A., Waziri G.D., Shehu S.M. (2014). Epstein-Barr Virus Association with Malignant Lymphoma Subgroups in Zaria, Nigeria. Niger J. Surg. Sci..

[69] Kaymaz Y., Oduor C.I., Yu H., Otieno J.A., Ong’echa J.M., Moormann A.M., Bailey J.A. (2017). Comprehensive Transcriptome and Mutational Profiling of Endemic Burkitt Lymphoma Reveals EBV Type-Specific Differences. Mol. Cancer Res..

[70] Klein G., Geering G., Old L.J., Henle G., Henle W., Clifford P. (1970). Comparison of the anti-EBV titer and the EBV-associated membrane reactive and precipitating antibody levels in the sera of Burkitt lymphoma and nasopharyngeal carcinoma patients and controls. Int. J. Cancer.

[71] Klein G., Pearson G., Henle G., Henle W., Goldstein G., Clifford P. (1969). Relation between Epstein-Barr viral and cell membrane immunofluorescence in Burkitt tumor cells. 3. Comparison of blocking of direct membrane immunofluorescence and anti-EBV reactivities of different sera. J. Exp. Med..

[72] Labrecque L.G., Xue S.A., Kazembe P., Phillips J., Lampert I., Wedderburn N., Griffin B.E. (1999). Expression of Epstein-Barr virus lytically related genes in African Burkitt’s lymphoma: Correlation with patient response to therapy. Int. J. Cancer.

[73] Lindahl T., Klein G., Reedman B.M., Johansson B., Singh S. (1974). Relationship between Epstein-Barr virus (EBV) DNA and the EBV-determined nuclear antigen (EBNA) in Burkitt lymphoma biopsies and other lymphoproliferative malignancies. Int. J. Cancer.

[74] Muddathir A.R.M., Elradi W.E.O., Yousif B., Abd allah E.I. (2020). Frequency of Epstein Barr virus infection among Sudanese children patients with Burkitt’s lymphoma. Int. J. Adv. Med..

[75] Mutalima N., Molyneux E., Jaffe H., Kamiza S., Borgstein E., Mkandawire N., Liomba G., Batumba M., Lagos D., Gratrix F. (2008). Associations between Burkitt lymphoma among children in Malawi and infection with HIV, EBV and malaria: Results from a case-control study. PLoS ONE.

[76] Nadkarni J.S., Nadkarni J.J., Klein G., Henle W., Henle G., Clifford P. (1970). EB viral antigens in Burkitt tumor biopsies and early cultures. Int. J. Cancer.

[77] Ndede I., Mining S.K., Patel K., Wanjala F.M., Tenge C.N. (2019). Epstein barr virus IgG and EBER-1 in Burkitt’s lymphoma children at a referral hospital in western Kenya. Pan Afr. Med. J..

[78] Niedobitek G., Agathanggelou A., Rowe M., Jones E.L., Jones D.B., Turyaguma P., Oryema J., Wright D.H., Young L.S. (1995). Heterogeneous expression of Epstein-Barr virus latent proteins in endemic Burkitt’s lymphoma. Blood.

[79] Nonoyama M., Huang C.H., Pagano J.S., Klein G., Singh S. (1973). DNA of Epstein-Barr virus detected in tissue of Burkitt’s lymphoma and nasopharyngeal carcinoma. Proc. Natl. Acad. Sci. USA.

[80] Nonoyama M., Kawai Y., Pagano J.S. (1975). Detection of Epstein-Barr virus DNA in human tumors. Bibl Haematol..

[81] Olweny C.L., Atine I., Kaddu-Mukasa A., Owor R., Andersson-Anvret M., Klein G., Henle W., de-The G. (1977). Epstein-Barr virus genome studies in Burkitt’s and non-Burkitt’s lymphomas in Uganda. J. Natl. Cancer Inst..

[82] Onwubuya I.M., Adelusola K.A., Durosinmi M.A., Sabageh D., Ezike K.N. (2015). Lymphomas in Ile-Ife, Nigeria: Immunohistochemical Characterization and Detection of Epstein-Barr virus Encoded RNA. J. Clin. Diagn. Res..

[83] Prevot S., Hamilton-Dutoit S., Audouin J., Walter P., Pallesen G., Diebold J. (1992). Analysis of African Burkitt’s and high-grade B cell non-Burkitt’s lymphoma for Epstein-Barr virus genomes using in situ hybridization. Br. J. Haematol..

[84] Reedman B.M., Klein G., Pope J.H., Walters M.K., Hilgers J., Singh S., Johansson B. (1974). Epstein-Barr virus-associated complement-fixing and nuclear antigens in Burkitt lymphoma biopsies. Int. J. Cancer.

[85] Stevens S.J., Pronk I., Middeldorp J.M. (2001). Toward standardization of Epstein-Barr virus DNA load monitoring: Unfractionated whole blood as preferred clinical specimen. J. Clin. Microbiol..

[86] Sutherland J.C., Olweny C.L., Levine P.H., Mardiney M.R. (1978). Epstein-Barr virus-immune complexes in postmortem kidneys from African patients with Burkitt’s lymphoma and American patients with and without lymphoma. J. Natl. Cancer Inst..

[87] Tao Q., Robertson K.D., Manns A., Hildesheim A., Ambinder R.F. (1998). Epstein-Barr virus (EBV) in endemic Burkitt’s lymphoma: Molecular analysis of primary tumor tissue. Blood.

[88] Tumwine L.K., Orem J., Kerchan P., Byarugaba W., Pileri S.A. (2010). EBV, HHV8 and HIV in B cell non Hodgkin lymphoma in Kampala, Uganda. Infect. Agents Cancer.

[89] Westmoreland K.D., Montgomery N.D., Stanley C.C., El-Mallawany N.K., Wasswa P., van der Gronde T., Mtete I., Butia M., Itimu S., Chasela M. (2017). Plasma Epstein-Barr virus DNA for pediatric Burkitt lymphoma diagnosis, prognosis and response assessment in Malawi. Int. J. Cancer.

[90] Xue S.A., Labrecque L.G., Lu Q.L., Ong S.K., Lampert I.A., Kazembe P., Molyneux E., Broadhead R.L., Borgstein E., Griffin B.E. (2002). Promiscuous expression of Epstein-Barr virus genes in Burkitt’s lymphoma from the central African country Malawi. Int. J. Cancer.

[91] Akyol G., Sezer C., Poyraz A., Ataoğlu Ö., Çelik B., Uluoğlu Ö., Edali N. (1997). Epstein-barr virüs dna’sinin in-situ hibridizasyon yöntemi ile saptanmasi: Nazofarengeal karsinoma, burkitt ve non-burkitt lenfomalar. KBB Ve Baş Boyun Cerrahisi Derg..

[92] Al-Fahdawi K.A., Al-Zobae M.A., Al-Esawi A.O.J.i. (2016). Association Between Epstein-Barr Virus and Burkitt’s lymphoma in Western Iraq. (A molecular case-control study). Infection.

[93] Anwar N., Kingma D.W., Bloch A.R., Mourad M., Raffeld M., Franklin J., Magrath I., Bolkainy N.E., Jaffe E.S. (1995). The investigation of epstein–barr viral sequences in 41 cases of Burkitt’s lymphoma from Egypt. Epidemiologic correlations. Cancer.

[94] Cavdar A.O., Gozdasoglu S., Yavuz G., Babacan E., Unal E., Uluoglu O., Yucesan S., Magrath I.T., Akar N. (1993). Burkitt’s lymphoma between African and American types in Turkish children: Clinical, viral (EBV), and molecular studies. Med. Pediatr. Oncol..

[95] Cavdar A.O., Yavuz G., Babacan E., Gozdasoglu S., Unal E., Ertem U., Pamir A., Yucesan S., Gokcora H., Uluoglu O. (1994). Burkitt’s lymphoma in Turkish children: Clinical, viral [EBV] and molecular studies. Leuk. Lymphoma.

[96] Gotleib-Stematsky T., Ramot B., Vonsover B., Aghai E., Kende G., Ninio M., Modan M. (1976). Antibodies to Epstein-Barr viral capsid and early antigens associated with Burkitt’s lymphoma and lymphoblastic lymphosarcoma in Israel. J. Natl. Cancer Inst..

[97] Habeeb R., Al Hafar L., Monem F. (2021). Plasma Epstein-Barr Virus (Ebv) DNA as a Biomarker for Diagnosis of Syrian Ebv-Positive Burkitt’s Lymphoma. Bull. Pharm. Sci. Assiut.

[98] Kabyemera R., Masalu N., Rambau P., Kamugisha E., Kidenya B., De Rossi A., Petrara M.R., Mwizamuholya D. (2013). Relationship between non-Hodgkin’s lymphoma and blood levels of Epstein-Barr virus in children in north-western Tanzania: A case control study. BMC Pediatr..

[99] Peylan-Ramu N., Diment J., Krichevsky S., Ben-Yehuda D., Bhatia K., Magrath I.T. (2001). Expression of EBV encoded nuclear small non-polyadenylated RNA (EBER) molecules in 32 cases of childhood Burkitt’s lymphoma from Israel. Leuk. Lymphoma.

[100] Sulitzeanu D., Szigeti R., Hatzubai A., Dillner J., Hammarskjold M.L., Klein G., Klein E. (1988). Antibodies in human sera against the Epstein-Barr virus encoded latent membrane protein (LMP). Immunol. Lett..

[101] Syrjanen S., Kallio P., Sainio P., Fuju C., Syrjanen K. (1992). Epstein-Barr virus (EBV) genomes and c-myc oncogene in oral Burkitt’s lymphomas. Scand. J. Dent. Res..

[102] Tacyildiz N., Cavdar A.O., Ertem U., Oksal A., Kutluay L., Uluoglu O., Lin J.C. (1998). Unusually high frequency of a 69-bp deletion within the carboxy terminus of the LMP-1 oncogene of Epstein-Barr virus detected in Burkitt’s lymphoma of Turkish children. Leukemia.

[103] Tinguely M., Brundler M.A., Gogos S., Kerl K., Borisch B. (2000). Epstein-Barr virus association in pediatric abdominal non-Hodgkin-lymphomas from Turkey. Arch. Immunol. Ther. Exp..

[104] Uccini S., Al-Jadiry M.F., Cippitelli C., Talerico C., Scarpino S., Al-Darraji A.F., Al-Badri S.A.F., Alsaadawi A.R., Al-Hadad S.A., Ruco L. (2018). Burkitt lymphoma in Iraqi children: A distinctive form of sporadic disease with high incidence of EBV(+) cases and more frequent expression of MUM1/IRF4 protein in cases with head and neck presentation. Pediatr. Blood Cancer.

[105] Araujo I., Foss H.D., Bittencourt A., Hummel M., Demel G., Mendonca N., Herbst H., Stein H. (1996). Expression of Epstein-Barr virus-gene products in Burkitt’s lymphoma in Northeast Brazil. Blood.

[106] Bacchi C.E., Bacchi M.M., Rabenhorst S.H., Soares F.A., Fonseca L.E., Barbosa H.S., Weiss L.M., Gown A.M. (1996). AIDS-related lymphoma in Brazil. Histopathology, immunophenotype, and association with Epstein-Barr virus. Am. J. Clin. Pathol..

[107] Chabay P.A., De Matteo E.N., Aversa L., Maglio S., Grinstein S., Preciado M.V. (2002). Assessment of Epstein-Barr virus association with pediatric non-hodgkin lymphoma in immunocompetent and in immunocompromised patients in Argentina. Arch. Pathol. Lab. Med..

[108] Chen W.G., Chen Y.Y., Bacchi M.M., Bacchi C.E., Alvarenga M., Weiss L.M. (1996). Genotyping of Epstein-Barr virus in Brazilian Burkitt’s lymphoma and reactive lymphoid tissue. Type A with a high prevalence of deletions within the latent membrane protein gene. Am. J. Pathol..

[109] Drut R.M., Day S., Drut R., Meisner L. (1994). Demonstration of Epstein-Barr viral DNA in paraffin-embedded tissues of Burkitt’s lymphoma from Argentina using the polymerase chain reaction and in situ hybridization. Pediatr. Pathol..

[110] Figueira C.M.G. (2006). Deteccao do Genoma Viral em Tecido Neoplasico e Analise da Translocacao Cromossomial em Pacientes com Linfoma de Burkitt Associado ao Virus de Epstein-Barr em Diferentes Regioes do Brasil. Ph.D. Thesis.

[111] Gutierrez M.I., Bhatia K., Barriga F., Diez B., Muriel F.S., de Andreas M.L., Epelman S., Risueno C., Magrath I.T. (1992). Molecular epidemiology of Burkitt’s lymphoma from South America: Differences in breakpoint location and Epstein-Barr virus association from tumors in other world regions. Blood.

[112] Hamilton-Dutoit S., Pallesen G., Franzmann M., Karkov J., Black F., Skinhøj P., Pedersen C. (1991). AIDS-related lymphoma. Histopathology, immunophenotype, and association with Epstein-Barr virus as demonstrated by in situ nucleic acid hybridization. Am. J. Clin. Pathol..

[113] Hassan R., Klumb C.E., Felisbino F.E., Guiretti D.M., White L.R., Stefanoff C.G., Barros M.H., Seuanez H.N., Zalcberg I.R. (2008). Clinical and demographic characteristics of Epstein-Barr virus-associated childhood Burkitt’s lymphoma in Southeastern Brazil: Epidemiological insights from an intermediate risk region. Haematologica.

[114] Hassan R., White L.R., Stefanoff C.G., de Oliveira D.E., Felisbino F.E., Klumb C.E., Bacchi C.E., Seuanez H.N., Zalcberg I.R. (2006). Epstein-Barr virus (EBV) detection and typing by PCR: A contribution to diagnostic screening of EBV-positive Burkitt’s lymphoma. Diagn. Pathol..

[115] Klumb C.E., Hassan R., De Oliveira D.E., De Resende L.M., Carrico M.K., De Almeida Dobbin J., Pombo-De-Oliveira M.S., Bacchi C.E., Maia R.C. (2004). Geographic variation in Epstein-Barr virus-associated Burkitt’s lymphoma in children from Brazil. Int. J. Cancer.

[116] Lara J., Cohen M., De Matteo E., Aversa L., Preciado M.V., Chabay P. (2014). Epstein-Barr virus (EBV) association and latency profile in pediatric Burkitt’s lymphoma: Experience of a single institution in Argentina. J. Med. Virol..

[117] Minnicelli C., Barros M.H., Klumb C.E., Romano S.O., Zalcberg I.R., Hassan R. (2012). Relationship of Epstein-Barr virus and interleukin 10 promoter polymorphisms with the risk and clinical outcome of childhood Burkitt lymphoma. PLoS ONE.

[118] Monteiro T.A.F., Barros V.L.S., Arnaud M.V.C., Meneses M.R.C., Silva S.S., Dias D.M., Monteiro J.L.F. (2009). Vírus de Epstein Barr (EBV) em tecido de pacientes com linfoma de Burkitt (LB) residentes na região amazônica. Rev. Soc. Bras. Med. Trop..

[119] Monteiro T.A.F., Arnaud M.V.C., Barros V.L.S., Meneses M.R.C., Monteiro J.L.F., Polaro A.A. (2019). Epstein Barr Vírus (EBV): EBER 1 Gene of EBV in Cases of Burkitt Lymphoma Attending in a Reference Hospital for Cancer in Northern Brazil. Universidade Federal do Pará. Pós-Graduação em Oncologia e Ciências Médicas. https://patuaback.iec.gov.br/server/api/core/bitstreams/24ef5d60-79dc-47c9-b50d-f52211197969/content.

[120] Pizza M., Bruniera P., Luporini S.M., Marcelino da Silva H.R., Borsato M.L., de Castro H.C., Soares F.A., Paes R.A. (2008). Detection of Epstein-Barr virus in children and adolescents with Burkitt’s lymphoma by in situ hybridization using tissue microarrays. Hematology.

[121] Queiroga E.M., Gualco G., Chioato L., Harrington W.J., Araujo I., Weiss L.M., Bacchi C.E. (2008). Viral studies in burkitt lymphoma: Association with Epstein-Barr virus but not HHV-8. Am. J. Clin. Pathol..

[122] Riverend E., Rengifo E., Longchong M., Ruiz R., Tormo B., Garcia J., Portero R., Quintero S., Valdes M., Rodriguez T. (1984). Burkitt’s lymphoma in Cuba. I. Clinical and morphological features and EBV association. Oncology.

[123] Deyhimi P., Kalantari M. (2014). Study of Epstein-Barr virus expression in Burkitt’s lymphoma by polymerase chain reaction and in situ hybridization: A study in Iran. Dent. Res. J..

[124] Mansoor A., Stevenson M.S., Li R.Z., Frekko K., Weiss W., Ahmad M., Khan A.H., Mushtaq S., Saleem M., Raffeld M. (1997). Prevalence of Epstein-Barr viral sequences and EBV LMP1 oncogene deletions in Burkitt’s lymphoma from Pakistan: Epidemiological correlations. Hum. Pathol..

[125] Sinha M., Rao C.R., Premalata C.S., Shafiulla M., Lakshmaiah K.C., Jacob L.A., Babu G.K., Viveka B.K., Appaji L., Subramanyam J.R. (2016). Plasma Epstein-Barr virus and Hepatitis B virus in non-Hodgkin lymphomas: Two lymphotropic, potentially oncogenic, latently occurring DNA viruses. Indian J. Med. Paediatr. Oncol..

[126] Bingler M.A., Feingold B., Miller S.A., Quivers E., Michaels M.G., Green M., Wadowsky R.M., Rowe D.T., Webber S.A. (2008). Chronic high Epstein-Barr viral load state and risk for late-onset posttransplant lymphoproliferative disease/lymphoma in children. Am. J. Transplant..

[127] Edwards R.H., Raab-Traub N. (1994). Alterations of the p53 gene in Epstein-Barr virus-associated immunodeficiency-related lymphomas. J. Virol..

[128] Guarner J., Rio C.D., Carr D., Hendrix L.E., Eley J.W., Unger E.R. (1991). Non-hodgkin’s lymphomas in patients with human immunodeficiency virus infection. Presence of epstein–barr virus byIn situ hybridization, clinical presentation, and follow-up. Cancer.

[129] Gulley M.L., Sargeant K.P., Grider D.J., Eagan P.A., Davey D.D., Damm D.D., Robinson R.A., Vandersteen D.P., McGuff H.S., Banks P.M. (1995). Lymphomas of the oral soft tissues are not preferentially associated with latent or replicative Epstein-Barr virus. Oral Surg. Oral Med. Oral Pathol. Oral Radiol..

[130] Levine P.H., O’Conor G.T., Bernard C.W. (1972). Antibodies to Epstein-Barr virus (EBV) in American patients with Burkitt’s lymphoma. Cancer.

[131] Liebowitz D. (1998). Epstein-Barr virus and a cellular signaling pathway in lymphomas from immunosuppressed patients. N. Engl. J. Med..

[132] Mbulaiteye S.M., Pullarkat S.T., Nathwani B.N., Weiss L.M., Rao N., Emmanuel B., Lynch C.F., Hernandez B., Neppalli V., Hawes D. (2014). Epstein-Barr virus patterns in US Burkitt lymphoma tumors from the SEER residual tissue repository during 1979–2009. APMIS.

[133] Nonoyama M., Kawai Y., Huang C.H., Pagano J.S., Hirshaut Y., Levine P.H. (1974). Epstein-Barr virus DNA in Hodgkin’s disease, American Burkitt’s lymphoma, and other human tumors. Cancer Res..

[134] Pagano J.S., Huang C.H., Levine P. (1973). Absence of Epstein-Barr viral DNA in Amercian Burkitt’s lymphoma. N. Engl. J. Med..

[135] Razzouk B.I., Srinivas S., Sample C.E., Singh V., Sixbey J.W. (1996). Epstein-Barr Virus DNA recombination and loss in sporadic Burkitt’s lymphoma. J. Infect. Dis..

[136] Subar M., Neri A., Inghirami G., Knowles D.M., Dalla-Favera R. (1988). Frequent c-myc oncogene activation and infrequent presence of Epstein-Barr virus genome in AIDS-associated lymphoma. Blood.

[137] Teitell M.A., Lones M.A., Perkins S.L., Sanger W.G., Cairo M.S., Said J.W. (2005). TCL1 expression and Epstein-Barr virus status in pediatric Burkitt lymphoma. Am. J. Clin. Pathol..

[138] Căinap S., Răchisan A., Fetică B., Cosnarovici R., Mihut E., Popa G., Gheban D., Căinap C. (2012). EBV in pediatric neoplasia–intensity of infection as independent prognostic factor. J. Med. Life.

[139] Camilleri-Broet S., Davi F., Feuillard J., Bourgeois C., Seilhean D., Hauw J.J., Raphael M. (1995). High expression of latent membrane protein 1 of Epstein-Barr virus and BCL-2 oncoprotein in acquired immunodeficiency syndrome-related primary brain lymphomas. Blood.

[140] Carbone A., Gloghini A., Zagonel V., Tirelli U. (1996). Expression of Epstein-Barr virus-encoded latent membrane protein 1 in nonendemic Burkitt’s lymphomas. Blood.

[141] Carbone A., Gloghini A., Zanette I., Canal B., Volpe R. (1993). Demonstration of Epstein-Barr viral genomes by in situ hybridization in acquired immune deficiency syndrome-related high grade and anaplastic large cell CD30+ lymphomas. Am. J. Clin. Pathol..

[142] Gonin J., Larousserie F., Bastard C., Picquenot J.M., Couturier J., Radford-Weiss I., Dietrich C., Brousse N., Vacher-Lavenu M.C., Devergne O. (2011). Epstein-Barr virus-induced gene 3 (EBI3): A novel diagnosis marker in Burkitt lymphoma and diffuse large B-cell lymphoma. PLoS ONE.

[143] Grasser F.A., Murray P.G., Kremmer E., Klein K., Remberger K., Feiden W., Reynolds G., Niedobitek G., Young L.S., Mueller-Lantzsch N. (1994). Monoclonal antibodies directed against the Epstein-Barr virus-encoded nuclear antigen 1 (EBNA1): Immunohistologic detection of EBNA1 in the malignant cells of Hodgkin’s disease. Blood.

[144] Hamilton-Dutoit S.J., Raphael M., Audouin J., Diebold J., Lisse I., Pedersen C., Oksenhendler E., Marelle L., Pallesen G. (1993). In situ demonstration of Epstein-Barr virus small RNAs (EBER 1) in acquired immunodeficiency syndrome-related lymphomas: Correlation with tumor morphology and primary site. Blood.

[145] Hamilton-Dutoit S.J., Rea D., Raphael M., Sandvej K., Delecluse H.J., Gisselbrecht C., Marelle L., van Krieken H.J., Pallesen G. (1993). Epstein-Barr virus-latent gene expression and tumor cell phenotype in acquired immunodeficiency syndrome-related non-Hodgkin’s lymphoma. Correlation of lymphoma phenotype with three distinct patterns of viral latency. Am. J. Pathol..

[146] Hummel M., Anagnostopoulos I., Korbjuhn P., Stein H. (1995). Epstein-Barr virus in B-cell non-Hodgkin’s lymphomas: Unexpected infection patterns and different infection incidence in low- and high-grade types. J. Pathol..

[147] Kersten M.J., Van Gorp J., Pals S.T., Boon F., Van Oers M.H. (1998). Expression of Epstein-Barr virus latent genes and adhesion molecules in AIDS-related non-Hodgkin’s lymphomas: Correlation with histology and CD4-cell number. Leuk. Lymphoma.

[148] Marchini B., Dolcher M.P., Sabbatini A., Klein G., Migliorini P. (1994). Immune response to different sequences of the EBNA I molecule in Epstein-Barr virus-related disorders and in autoimmune diseases. J. Autoimmun..

[149] Mundo L., Ambrosio M.R., Picciolini M., Lo Bello G., Gazaneo S., Del Porro L., Lazzi S., Navari M., Onyango N., Granai M. (2017). Unveiling Another Missing Piece in EBV-Driven Lymphomagenesis: EBV-Encoded MicroRNAs Expression in EBER-Negative Burkitt Lymphoma Cases. Front. Microbiol..

[150] Ometto L., Menin C., Masiero S., Bonaldi L., Del Mistro A., Cattelan A.M., D’Andrea E., De Rossi A., Chieco-Bianchi L.J.B. (1997). Molecular profile of Epstein-Barr virus in human immunodeficiency virus type 1–related lymphadenopathies and lymphomas. Blood.

[151] Pallesen G., Hamilton-Dutoit S.J., Rowe M., Lisse I., Ralfkiaer E., Sandvej K., Young L.S. (1991). Expression of Epstein-Barr virus replicative proteins in AIDS-related non-Hodgkin’s lymphoma cells. J. Pathol..

[152] Parolini O., Kagerbauer B., Simonitsch-Klupp I., Ambros P., Jaeger U., Mann G., Haas O.A., Morra M., Gadner H., Terhorst C. (2002). Analysis of SH2D1A mutations in patients with severe Epstein-Barr virus infections, Burkitt’s lymphoma, and Hodgkin’s lymphoma. Ann. Hematol..

[153] Pearson G., Klein G., Henle G., Henle W., Clifford P. (1969). Relation between Epstein-Barr viral and cell membrane immunofluorescence in Burkitt tumor cells. IV. Differentiation between antibodies responsible for membrane and viral immunofluorescence. J. Exp. Med..

[154] Pedersen C., Gerstoft J., Lundgren J.D., Skinhoj P., Bottzauw J., Geisler C., Hamilton-Dutoit S.J., Thorsen S., Lisse I., Ralfkiaer E. (1991). HIV-associated lymphoma: Histopathology and association with Epstein-Barr virus genome related to clinical, immunological and prognostic features. Eur. J. Cancer.

[155] Rea D., Delecluse H.J., Hamilton-Dutoit S.J., Marelle L., Joab I., Edelman L., Finet J.F., Raphael M. (1994). Epstein-Barr virus latent and replicative gene expression in post-transplant lymphoproliferative disorders and AIDS-related non-Hodgkin’s lymphomas. French Study Group of Pathology for HIV-associated Tumors. Ann. Oncol..

[156] Chan J.K., Tsang W.Y., Ng C.S., Wong C.S., Lo E.S. (1995). A study of the association of Epstein-Barr virus with Burkitt’s lymphoma occurring in a Chinese population. Histopathology.

[157] Chao T.Y., Wang T.Y., Lee W. (1997). Association between Epstein-Barr virus and Burkitt’s lymphoma in Taiwan. Cancer.

[158] Chen B.J., Chang S.T., Weng S.F., Huang W.T., Chu P.Y., Hsieh P.P., Jung Y.C., Kuo C.C., Chuang Y.T., Chuang S.S. (2016). EBV-associated Burkitt lymphoma in Taiwan is not age-related. Leuk. Lymphoma.

[159] Cho E.Y., Kim K.H., Kim W.S., Yoo K.H., Koo H.H., Ko Y.H. (2008). The spectrum of Epstein-Barr virus-associated lymphoproliferative disease in Korea: Incidence of disease entities by age groups. J. Korean Med. Sci..

[160] Feng Y.F., Wu Q.L., Zong Y.S. (2007). Correlation of immunophenotype of sinonasal non-Hodgkin’s lymphoma to Epstein-Barr virus infection. Ai Zheng.

[161] Hishima T., Oyaizu N., Fujii T., Tachikawa N., Ajisawa A., Negishi M., Nakamura T., Iwamoto A., Hayashi Y., Matsubara D. (2006). Decrease in Epstein-Barr virus-positive AIDS-related lymphoma in the era of highly active antiretroviral therapy. Microbes Infect..

[162] Huang Y.H., Wu Q.L., Zong Y.S., Feng Y.F., Liang J.Z., Hou J.H., Shao Q., Fu J. (2009). Clinicopathologic features and Epstein-Barr virus infection status of Burkitt’s lymphoma in Guangzhou district. Ai Zheng.

[163] Kim D., Ko Y., Suh Y., Koo H., Huh J., Lee W. (2005). Characteristics of Epstein-Barr virus associated childhood non-Hodgkin’s lymphoma in the Republic of Korea. Virchows Arch..

[164] Lam K.Y., Lo C.Y., Kwong D.L., Lee J., Srivastava G. (1999). Malignant lymphoma of the thyroid. A 30-year clinicopathologic experience and an evaluation of the presence of Epstein-Barr virus. Am. J. Clin. Pathol..

[165] Lee S.H., Su I.J., Chen R.L., Lin K.S., Lin D.T., Chuu W.M., Lin K.S. (1991). A pathologic study of childhood lymphoma in Taiwan with special reference to peripheral T-cell lymphoma and the association with Epstein-Barr viral infection. Cancer.

[166] Nomura Y., Lavu E.K., Muta K., Niino D., Takeshita M., Hirose S., Nakamura S., Yoshino T., Kikuchi M., Ohshima K. (2008). Histological characteristics of 21 Papua New Guinean children with high-grade B-cell lymphoma, which is frequently associated with EBV infection. Pathol. Int..

[167] Okano M., Kikuta H., Abo W., Koizumi S., Aya T., Yano S., Takada K., Mizuno F., Osato T. (1992). Frequent association of Epstein-Barr virus in Japanese patients with Burkitt’s lymphoma. Jpn. J. Clin. Oncol..

[168] Ouyang C., Deng Z., Zhou J., Fu C., Liu S., Tao Y., Xiao D. (2019). Chromatin remodeling factor lymphoid-specific helicase links with Epstein-Barr virus associated the follicular germinal center B cell lymphomas. J. Cancer Res. Ther..

[169] Qin C., Huang Y., Feng Y., Li M., Guo N., Rao H. (2018). Clinicopathological features and EBV infection status of lymphoma in children and adolescents in South China: A retrospective study of 662 cases. Diagn. Pathol..

[170] Sakurai M., Hayashi Y., Abe R., Nakazawa S. (1983). Chromosome translocations, surface immunoglobulins, and Epstein-Barr virus in Japanese Burkitt’s lymphoma. Cancer Genet. Cytogenet..

[171] Satou A., Asano N., Nakazawa A., Osumi T., Tsurusawa M., Ishiguro A., Elsayed A.A., Nakamura N., Ohshima K., Kinoshita T. (2015). Epstein-Barr virus (EBV)-positive sporadic burkitt lymphoma: An age-related lymphoproliferative disorder?. Am. J. Surg. Pathol..

[172] Mitarnun W., Pradutkanchana J., Ishida T. (2004). Epstein-Barr virus-associated nodal malignant lymphoma in Thailand. Asian Pac. J. Cancer Prev..

[173] Peh S.C. (2001). Host ethnicity influences non-Hodgkin’s lymphoma subtype frequency and Epstein-Barr virus association rate: The experience of a multi-ethnic patient population in Malaysia. Histopathology.

[174] Peh S.C., Nadarajah V.S., Tai Y.C., Kim L.H., Abdullah W.A. (2004). Pattern of Epstein-Barr virus association in childhood non-Hodgkin’s lymphoma: Experience of university of malaya medical center. Pathol. Int..

[175] Abdelrahim L.M., Peh S.-C., Kallarakkal T.G. (2018). Epstein–Barr virus infection in B-cell Non-Hodgkin’s Lymphomas of the Oral and Maxillofacial Region: Is there any evidence?. Malays. J. Pathol..

[176] Boyle M.J., Sewell W.A., Sculley T.B., Apolloni A., Turner J.J., Swanson C.E., Penny R., Cooper D.A. (1991). Subtypes of Epstein-Barr virus in human immunodeficiency virus-associated non-Hodgkin lymphoma. Blood.

[177] Banatvala J.E., Best J.M., Waller D.K. (1972). Epstein-Barr virus-specific IgM in infectious mononucleosis, Burkitt lymphoma, nasopharyngeal carcinoma. Lancet.

[178] Hirshaut Y., Cohen M.H., Stevens D.A. (1973). Epstein-Barr-virus antibodies in American and African Burkitt’s lymphoma. Lancet.

[179] Joab I., Nicolas J.C., Schwaab G., de-The G., Clausse B., Perricaudet M., Zeng Y. (1991). Detection of anti-Epstein-Barr-virus transactivator (ZEBRA) antibodies in sera from patients with nasopharyngeal carcinoma. Int. J. Cancer.

[180] Rowe M., Rooney C.M., Edwards C.F., Lenoir G.M., Rickinson A.B. (1986). Epstein-Barr virus status and tumour cell phenotype in sporadic Burkitt’s lymphoma. Int. J. Cancer.

[181] Ambrosio M.R., Navari M., Di Lisio L., Leon E.A., Onnis A., Gazaneo S., Mundo L., Ulivieri C., Gomez G., Lazzi S. (2014). The Epstein Barr-encoded BART-6-3p microRNA affects regulation of cell growth and immuno response in Burkitt lymphoma. Infect. Agents Cancer.

[182] Bellan C., Lazzi S., Hummel M., Palummo N., de Santi M., Amato T., Nyagol J., Sabattini E., Lazure T., Pileri S.A. (2005). Immunoglobulin gene analysis reveals 2 distinct cells of origin for EBV-positive and EBV-negative Burkitt lymphomas. Blood.

[183] Lehtinen T., Isola J., Aine R., Alavaikko M., Lehtinen M. (1992). Accumulation of p53 protein correlates with tumour proliferative activity in EBV positive Burkitt’s lymphoma. Hematol. Oncol..

[184] Vargas-Ayala R.C., Jay A., Manara F., Maroui M.A., Hernandez-Vargas H., Diederichs A., Robitaille A., Sirand C., Ceraolo M.G., Romero-Medina M.C. (2019). Interplay between the Epigenetic Enzyme Lysine (K)-Specific Demethylase 2B and Epstein-Barr Virus Infection. J Virol.

[185] Barriga F., Kiwanuka J., Alvarez-Mon M., Shiramizu B., Huber B., Levine P., Magrath I. (1988). Significance of chromosome 8 breakpoint location in Burkitt’s lymphoma: Correlation with geographical origin and association with Epstein-Barr virus. Mechanisms in B-Cell Neoplasia 1988.

[186] Shiramizu B., Barriga F., Neequaye J., Jafri A., Dalla-Favera R., Neri A., Guttierez M., Levine P., Magrath I. (1991). Patterns of chromosomal breakpoint locations in Burkitt’s lymphoma: Relevance to geography and Epstein-Barr virus association. Blood.

[187] Quintanilla-Martínez L., Lome-Maldonado C., Ott G., Gschwendtner A., Gredler E., Reyes E., Angeles-Angeles A., Fend F. (1997). Primary Non-Hodgkin’s Lymphoma of the Intestine: High Prevalence of Epstein-Barr Virus in Mexican Lymphomas as Compared with European Cases. Blood.

[188] Rao C.R., Gutierrez M.I., Bhatia K., Fend F., Franklin J., Appaji L., Gallo G., O’Conor G., Lalitha N., Magrath I. (2000). Association of Burkitt’s lymphoma with the Epstein-Barr virus in two developing countries. Leuk. Lymphoma.

[189] Navari M., Etebari M., De Falco G., Ambrosio M.R., Gibellini D., Leoncini L., Piccaluga P.P. (2015). The presence of Epstein-Barr virus significantly impacts the transcriptional profile in immunodeficiency-associated Burkitt lymphoma. Front. Microbiol..

[190] Piccaluga P.P., Navari M., De Falco G., Ambrosio M.R., Lazzi S., Fuligni F., Bellan C., Rossi M., Sapienza M.R., Laginestra M.A. (2016). Virus-encoded microRNA contributes to the molecular profile of EBV-positive Burkitt lymphomas. Oncotarget.

[191] Epstein M.A., Achong B.G., Barr Y.M. (1964). Virus Particles in Cultured Lymphoblasts from Burkitt’s Lymphoma. Lancet.

[192] Epstein M.A., Barr Y.M. (1964). Cultivation in Vitro of Human Lymphoblasts from Burkitt’s Malignant Lymphoma. Lancet.

[193] Henle G., Henle W., Diehl V. (1968). Relation of Burkitt’s tumor-associated herpes-ytpe virus to infectious mononucleosis. Proc. Natl. Acad. Sci. USA.

[194] Bi C.F., Tang Y., Zhang W.Y., Zhao S., Wang X.Q., Yang Q.P., Li G.D., Liu W.P. (2012). Sporadic Burkitt lymphomas of children and adolescents in Chinese: A clinicopathological study of 43 cases. Diagn. Pathol..

[195] Huang H., Liu Z.L., Zeng H., Zhang S.H., Huang C.S., Xu H.Y., Wu Y., Zeng S.T., Xiong F., Yang W.P. (2015). Clinicopathological study of sporadic Burkitt lymphoma in children. Chin. Med. J..

[196] Yakimchuk K., Iravani M., Hasni M., Rhönnstad P., Nilsson S., Jondal M., Okret S.J.L. (2011). Effect of ligand-activated estrogen receptor β on lymphoma growth in vitro and in vivo. Leukemia.

[197] Jacobs B.M., Giovannoni G., Cuzick J., Dobson R. (2020). Systematic review and meta-analysis of the association between Epstein-Barr virus, multiple sclerosis and other risk factors. Mult. Scler..

[198] Bae J.-M., Kim E.H. (2016). Epstein-Barr Virus Infection and Risk of Breast Cancer: An Adaptive Meta-Analysis for Case-Control Studies. Arch. Clin. Infect. Dis..

[199] Pyo J.S., Kim N.Y., Kang D.W. (2020). Clinicopathological Significance of EBV-Infected Gastric Carcinomas: A Meta-Analysis. Medicina.

[200] Hu J., Zhang X., Tao H., Jia Y. (2022). The prognostic value of Epstein-Barr virus infection in Hodgkin lymphoma: A systematic review and meta-analysis. Front. Oncol..

[201] Abusalah M.A.H., Irekeola A.A., Hanim Shueb R., Jarrar M., Yean Yean C. (2022). Prognostic Epstein-Barr Virus (EBV) miRNA biomarkers for survival outcome in EBV-associated epithelial malignancies: Systematic review and meta-analysis. PLoS ONE.

[202] Balfour H.H., Meirhaeghe M.R., Stancari A.L., Geris J.M., Condon L.M., Cederberg L.E. (2022). Declining Epstein-Barr Virus Antibody Prevalence in College Freshmen Strengthens the Rationale for a Prophylactic EBV Vaccine. Vaccines.

[203] Zhong L., Krummenacher C., Zhang W., Hong J., Feng Q., Chen Y., Zhao Q., Zeng M.S., Zeng Y.X., Xu M. (2022). Urgency and necessity of Epstein-Barr virus prophylactic vaccines. NPJ Vaccines.

[204] Gulley M.L., Raab-Traub N. (1993). Detection of Epstein-Barr virus in human tissues by molecular genetic techniques. Arch. Pathol. Lab. Med..

[205] Cao P., Zhang M., Wang W., Dai Y., Sai B., Sun J., Wang L., Wang F., Li G., Xiang J. (2017). Fluorescence in situ hybridization is superior for monitoring Epstein Barr viral load in infectious mononucleosis patients. BMC Infect. Dis..

[206] Fan H., Gulley M.L. (2001). Molecular Methods for Detecting Epstein-Barr Virus (Part I): In Situ Hybridization to Epstein-Barr Virus-Encoded RNA (EBER) Transcripts. Methods Mol. Med..

[207] Weiss L.M., Chen Y.Y. (2013). EBER in situ hybridization for Epstein-Barr virus. Methods in Molecular Biology.

[208] Khan G., Fitzmaurice C., Naghavi M., Ahmed L.A. (2020). Global and regional incidence, mortality and disability-adjusted life-years for Epstein-Barr virus-attributable malignancies, 1990–2017. BMJ Open.

[209] Fitzmaurice C., Abate D., Abbasi N., Abbastabar H., Abd-Allah F., Abdel-Rahman O., Abdelalim A., Abdoli A., Abdollahpour I., Global Burden of Disease Cancer Collaboration (2019). Global, Regional, and National Cancer Incidence, Mortality, Years of Life Lost, Years Lived With Disability, and Disability-Adjusted Life-Years for 29 Cancer Groups, 1990 to 2017: A Systematic Analysis for the Global Burden of Disease Study. JAMA Oncol..

