# Supplementary material for: Worldwide Prevalence of Epstein–Barr Virus in Patients with Burkitt Lymphoma: A Systematic Review and Meta-Analysis
Source: Diagnostics (Basel). 2023 Jun 15;13(12):2068. doi: 10.3390/diagnostics13122068 (PMC10296943; doi:10.3390/diagnostics13122068)
Supplement: Supplementary file 1 [file diagnostics-13-02068-s001.zip › diagnostics-2281093-supplementary.pdf]

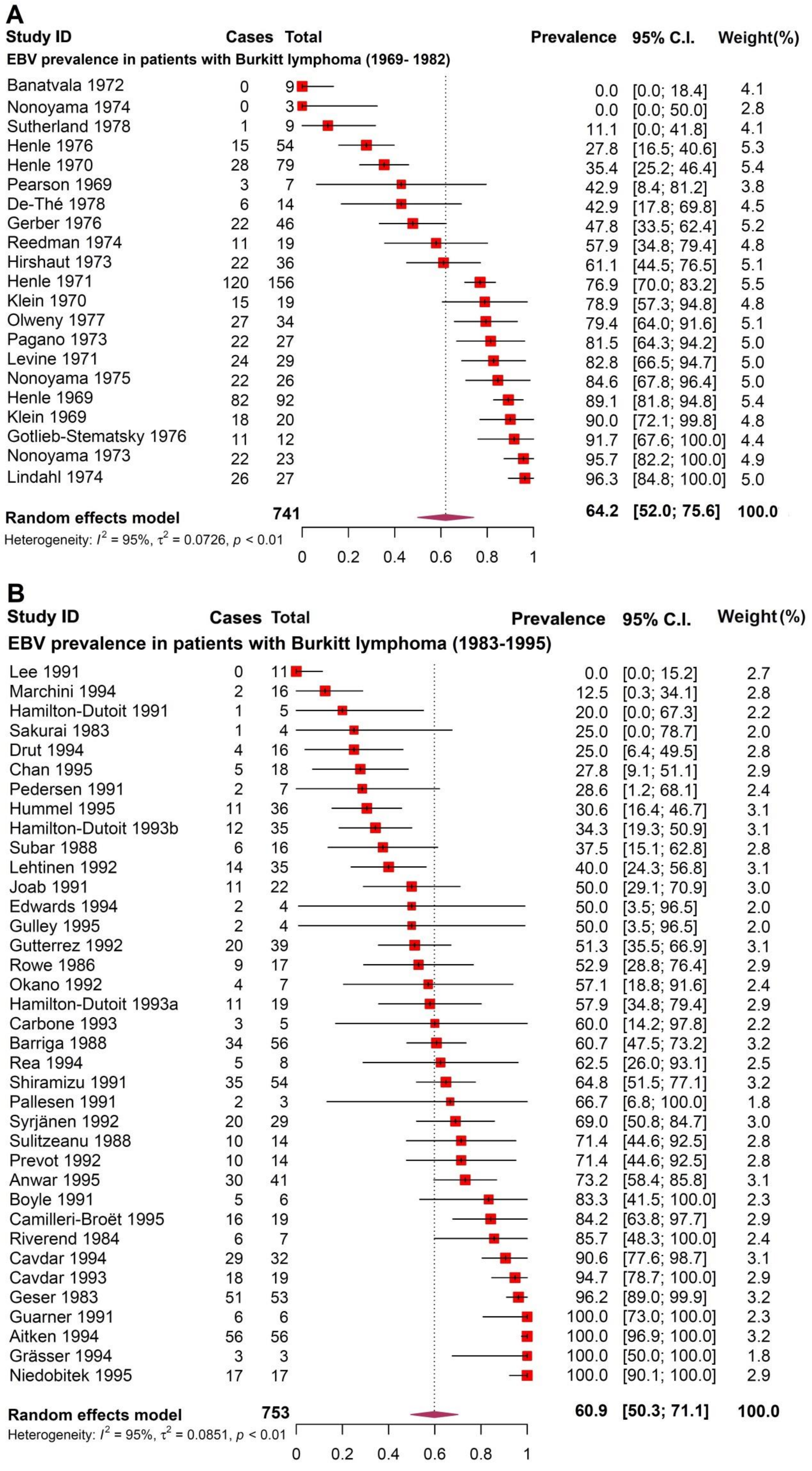
A
Study ID
Cases
Total
Prevalence
95% C.I.
Weight(%)
EBV prevalence in patients with Burkitt lymphoma (1969- 1982)
Banatvala 1972 0 9 0.0 [0.0; 18.4] 4.1
Nonoyama 1974 0 3 0.0 [0.0; 50.0] 2.8
Sutherland 1978 1 9 11.1 [0.0; 41.8] 4.1
Henle 1976 15 54 27.8 [16.5; 40.6] 5.3
Henle 1970 28 79 35.4 [25.2; 46.4] 5.4
Pearson 1969 3 7 42.9 [8.4; 81.2] 3.8
De-Thé 1978 6 14 42.9 [17.8; 69.8] 4.5
Gerber 1976 22 46 47.8 [33.5; 62.4] 5.2
Reedman 1974 11 19 57.9 [34.8; 79.4] 4.8
Hirshaut 1973 22 36 61.1 [44.5; 76.5] 5.1
Henle 1971 120 156 76.9 [70.0; 83.2] 5.5
Klein 1970 15 19 78.9 [57.3; 94.8] 4.8
Olweny 1977 27 34 79.4 [64.0; 91.6] 5.1
Pagano 1973 22 27 81.5 [64.3; 94.2] 5.0
Levine 1971 24 29 82.8 [66.5; 94.7] 5.0
Nonoyama 1975 22 26 84.6 [67.8; 96.4] 5.0
Henle 1969 82 92 89.1 [81.8; 94.8] 5.4
Klein 1969 18 20 90.0 [72.1; 99.8] 4.8
Gotlieb-Stematsky 1976 11 12 91.7 [67.6; 100.0] 4.4
Nonoyama 1973 22 23 95.7 [82.2; 100.0] 4.9
Lindahl 1974 26 27 96.3 [84.8; 100.0] 5.0
Random effects model 741 64.2 [52.0; 75.6] 100.0
Heterogeneity: I2 = 95%, τ2 = 0.0726, p < 0.01
0 0.2 0.4 0.6 0.8 1
B
Study ID
Cases
Total
Prevalence
95% C.I.
Weight (%)
EBV prevalence in patients with Burkitt lymphoma (1983-1995)
Lee 1991 0 11 0.0 [0.0; 15.2] 2.7
Marchini 1994 2 16 12.5 [0.3; 34.1] 2.8
Hamilton-Dutoit 1991 1 5 20.0 [0.0; 67.3] 2.2
Sakurai 1983 1 4 25.0 [0.0; 78.7] 2.0
Drut 1994 4 16 25.0 [6.4; 49.5] 2.8
Chan 1995 5 18 27.8 [9.1; 51.1] 2.9
Pedersen 1991 2 7 28.6 [1.2; 68.1] 2.4
Hummel 1995 11 36 30.6 [16.4; 46.7] 3.1
Hamilton-Dutoit 1993b 12 35 34.3 [19.3; 50.9] 3.1
Subar 1988 6 16 37.5 [15.1; 62.8] 2.8
Lehtinen 1992 14 35 40.0 [24.3; 56.8] 3.1
Joab 1991 11 22 50.0 [29.1; 70.9] 3.0
Edwards 1994 2 4 50.0 [3.5; 96.5] 2.0
Gulley 1995 2 4 50.0 [3.5; 96.5] 2.0
Gutterrez 1992 20 39 51.3 [35.5; 66.9] 3.1
Rowe 1986 9 17 52.9 [28.8; 76.4] 2.9
Okano 1992 4 7 57.1 [18.8; 91.6] 2.4
Hamilton-Dutoit 1993a 11 19 57.9 [34.8; 79.4] 2.9
Carbone 1993 3 5 60.0 [14.2; 97.8] 2.2
Barriga 1988 34 56 60.7 [47.5; 73.2] 3.2
Rea 1994 5 8 62.5 [26.0; 93.1] 2.5
Shiramizu 1991 35 54 64.8 [51.5; 77.1] 3.2
Pallesen 1991 2 3 66.7 [6.8; 100.0] 1.8
Syrjänen 1992 20 29 69.0 [50.8; 84.7] 3.0
Sulitzeanu 1988 10 14 71.4 [44.6; 92.5] 2.8
Prevot 1992 10 14 71.4 [44.6; 92.5] 2.8
Anwar 1995 30 41 73.2 [58.4; 85.8] 3.1
Boyle 1991 5 6 83.3 [41.5; 100.0] 2.3
Camilleri-Broët 1995 16 19 84.2 [63.8; 97.7] 2.9
Riverend 1984 6 7 85.7 [48.3; 100.0] 2.4
Cavdar 1994 29 32 90.6 [77.6; 98.7] 3.1
Cavdar 1993 18 19 94.7 [78.7; 100.0] 2.9
Geser 1983 51 53 96.2 [89.0; 99.9] 3.2
Guarner 1991 6 6 100.0 [73.0; 100.0] 2.3
Aitken 1994 56 56 100.0 [96.9; 100.0] 3.2
Grässer 1994 3 3 100.0 [50.0; 100.0] 1.8
Niedobitek 1995 17 17 100.0 [90.1; 100.0] 2.9
Random effects model 753 60.9 [50.3; 71.1] 100.0
Heterogeneity: I2 = 95%, τ2 = 0.0851, p < 0.01
0 0.2 0.4 0.6 0.8 1

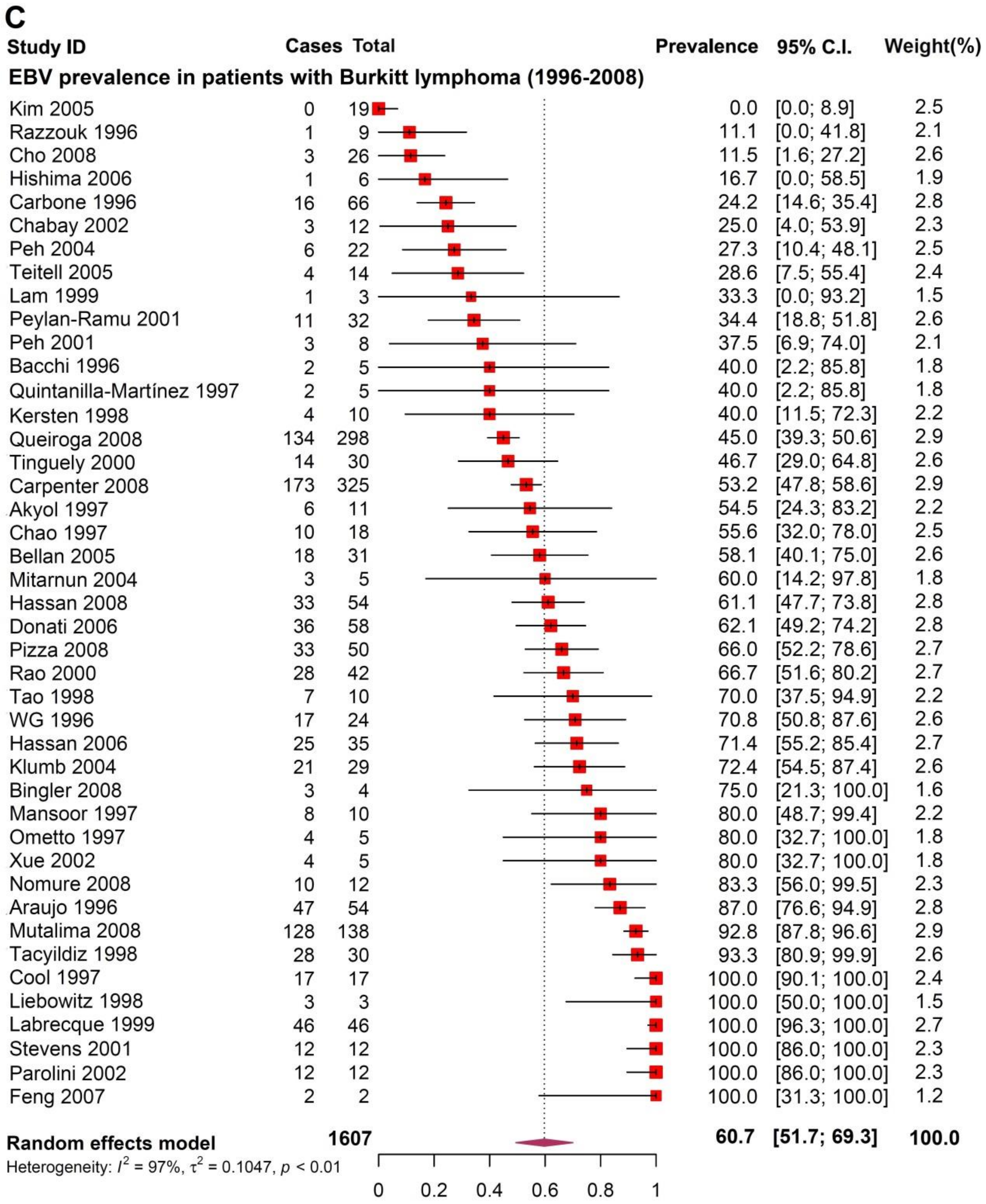
C
Study ID
Cases Total
Prevalence
95% C.I.
Weight(%)
EBV prevalence in patients with Burkitt lymphoma (1996-2008)
Kim 2005 0 19 0.0 [0.0; 8.9] 2.5
Razzouk 1996 1 9 11.1 [0.0; 41.8] 2.1
Cho 2008 3 26 11.5 [1.6; 27.2] 2.6
Hishima 2006 1 6 16.7 [0.0; 58.5] 1.9
Carbone 1996 16 66 24.2 [14.6; 35.4] 2.8
Chabay 2002 3 12 25.0 [4.0; 53.9] 2.3
Peh 2004 6 22 27.3 [10.4; 48.1] 2.5
Teitell 2005 4 14 28.6 [7.5; 55.4] 2.4
Lam 1999 1 3 33.3 [0.0; 93.2] 1.5
Peylan-Ramu 2001 11 32 34.4 [18.8; 51.8] 2.6
Peh 2001 3 8 37.5 [6.9; 74.0] 2.1
Bacchi 1996 2 5 40.0 [2.2; 85.8] 1.8
Quintanilla-Martínez 1997 2 5 40.0 [2.2; 85.8] 1.8
Kersten 1998 4 10 40.0 [11.5; 72.3] 2.2
Queiroga 2008 134 298 45.0 [39.3; 50.6] 2.9
Tinguely 2000 14 30 46.7 [29.0; 64.8] 2.6
Carpenter 2008 173 325 53.2 [47.8; 58.6] 2.9
Akyol 1997 6 11 54.5 [24.3; 83.2] 2.2
Chao 1997 10 18 55.6 [32.0; 78.0] 2.5
Bellan 2005 18 31 58.1 [40.1; 75.0] 2.6
Mitarnun 2004 3 5 60.0 [14.2; 97.8] 1.8
Hassan 2008 33 54 61.1 [47.7; 73.8] 2.8
Donati 2006 36 58 62.1 [49.2; 74.2] 2.8
Pizza 2008 33 50 66.0 [52.2; 78.6] 2.7
Rao 2000 28 42 66.7 [51.6; 80.2] 2.7
Tao 1998 7 10 70.0 [37.5; 94.9] 2.2
WG 1996 17 24 70.8 [50.8; 87.6] 2.6
Hassan 2006 25 35 71.4 [55.2; 85.4] 2.7
Klumb 2004 21 29 72.4 [54.5; 87.4] 2.6
Bingler 2008 3 4 75.0 [21.3; 100.0] 1.6
Mansoor 1997 8 10 80.0 [48.7; 99.4] 2.2
Ometto 1997 4 5 80.0 [32.7; 100.0] 1.8
Xue 2002 4 5 80.0 [32.7; 100.0] 1.8
Nomure 2008 10 12 83.3 [56.0; 99.5] 2.3
Araujo 1996 47 54 87.0 [76.6; 94.9] 2.8
Mutalima 2008 128 138 92.8 [87.8; 96.6] 2.9
Tacyildiz 1998 28 30 93.3 [80.9; 99.9] 2.6
Cool 1997 17 17 100.0 [90.1; 100.0] 2.4
Liebowitz 1998 3 3 100.0 [50.0; 100.0] 1.5
Labrecque 1999 46 46 100.0 [96.3; 100.0] 2.7
Stevens 2001 12 12 100.0 [86.0; 100.0] 2.3
Parolini 2002 12 12 100.0 [86.0; 100.0] 2.3
Feng 2007 2 2 100.0 [31.3; 100.0] 1.2
Random effects model 1607 60.7 [51.7; 69.3] 100.0
Heterogeneity: I^2 = 97%, τ^2 = 0.1047, p < 0.01
0 0.2 0.4 0.6 0.8 1

D

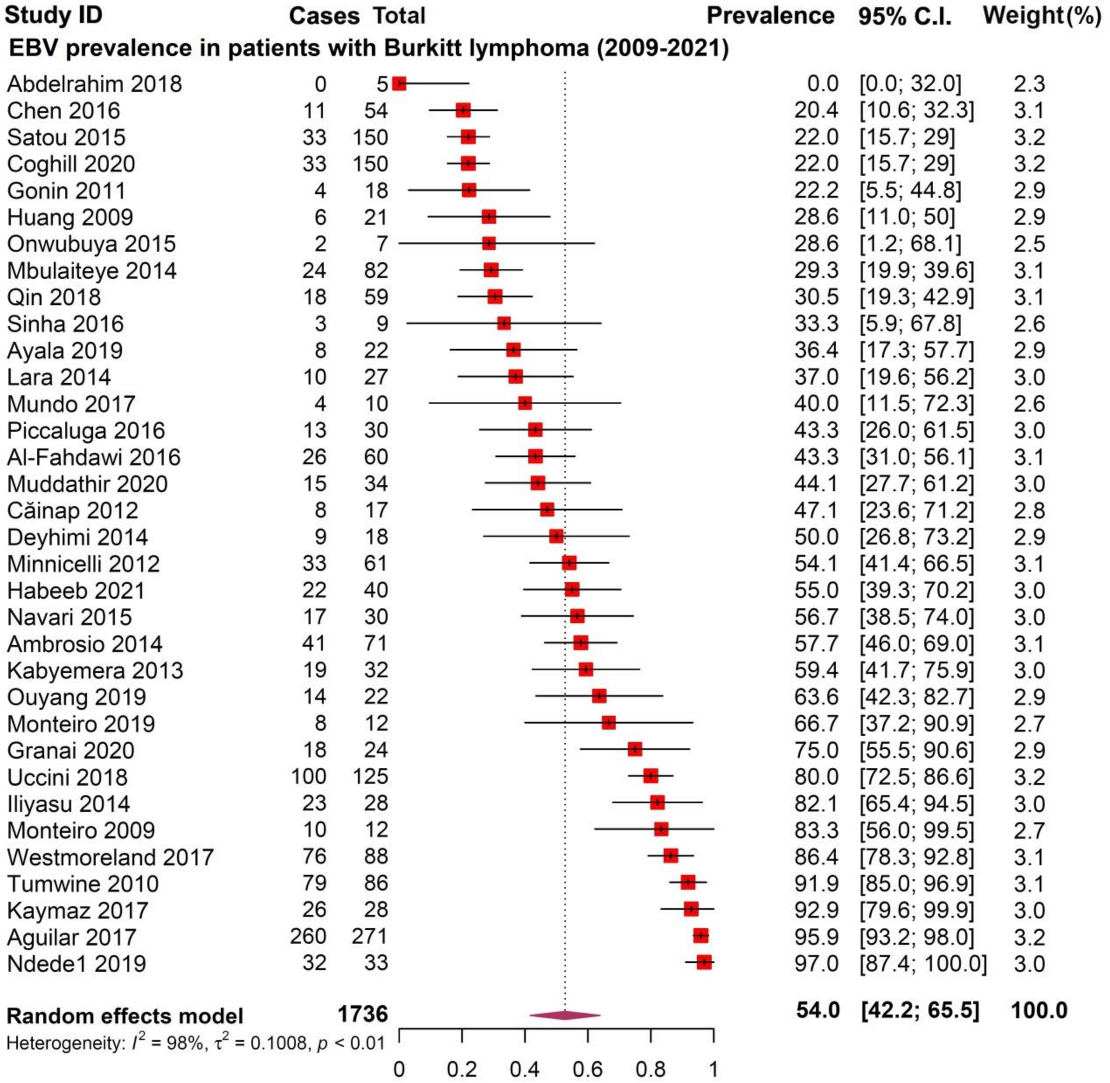
Study ID Cases Total Prevalence 95% C.I. Weight(%)
EBV prevalence in patients with Burkitt lymphoma (2009-2021)
Abdelrahim 2018 0 5 0.0 [0.0; 32.0] 2.3
Chen 2016 11 54 20.4 [10.6; 32.3] 3.1
Satou 2015 33 150 22.0 [15.7; 29] 3.2
Coghill 2020 33 150 22.0 [15.7; 29] 3.2
Gonin 2011 4 18 22.2 [5.5; 44.8] 2.9
Huang 2009 6 21 28.6 [11.0; 50] 2.9
Onwubuya 2015 2 7 28.6 [1.2; 68.1] 2.5
Mbulaiteye 2014 24 82 29.3 [19.9; 39.6] 3.1
Qin 2018 18 59 30.5 [19.3; 42.9] 3.1
Sinha 2016 3 9 33.3 [5.9; 67.8] 2.6
Ayala 2019 8 22 36.4 [17.3; 57.7] 2.9
Lara 2014 10 27 37.0 [19.6; 56.2] 3.0
Mundo 2017 4 10 40.0 [11.5; 72.3] 2.6
Piccaluga 2016 13 30 43.3 [26.0; 61.5] 3.0
Al-Fahdawi 2016 26 60 43.3 [31.0; 56.1] 3.1
Muddathir 2020 15 34 44.1 [27.7; 61.2] 3.0
Căinap 2012 8 17 47.1 [23.6; 71.2] 2.8
Deyhimi 2014 9 18 50.0 [26.8; 73.2] 2.9
Minnicelli 2012 33 61 54.1 [41.4; 66.5] 3.1
Habeeb 2021 22 40 55.0 [39.3; 70.2] 3.0
Navari 2015 17 30 56.7 [38.5; 74.0] 3.0
Ambrosio 2014 41 71 57.7 [46.0; 69.0] 3.1
Kabyemera 2013 19 32 59.4 [41.7; 75.9] 3.0
Ouyang 2019 14 22 63.6 [42.3; 82.7] 2.9
Monteiro 2019 8 12 66.7 [37.2; 90.9] 2.7
Granai 2020 18 24 75.0 [55.5; 90.6] 2.9
Uccini 2018 100 125 80.0 [72.5; 86.6] 3.2
Iliyasu 2014 23 28 82.1 [65.4; 94.5] 3.0
Monteiro 2009 10 12 83.3 [56.0; 99.5] 2.7
Westmoreland 2017 76 88 86.4 [78.3; 92.8] 3.1
Tumwine 2010 79 86 91.9 [85.0; 96.9] 3.1
Kaymaz 2017 26 28 92.9 [79.6; 99.9] 3.0
Aguilar 2017 260 271 95.9 [93.2; 98.0] 3.2
Ndede1 2019 32 33 97.0 [87.4; 100.0] 3.0
Random effects model 1736 54.0 [42.2; 65.5] 100.0
Heterogeneity: I^2 = 98%, τ^2 = 0.1008, p < 0.01
0 0.2 0.4 0.6 0.8 1

E

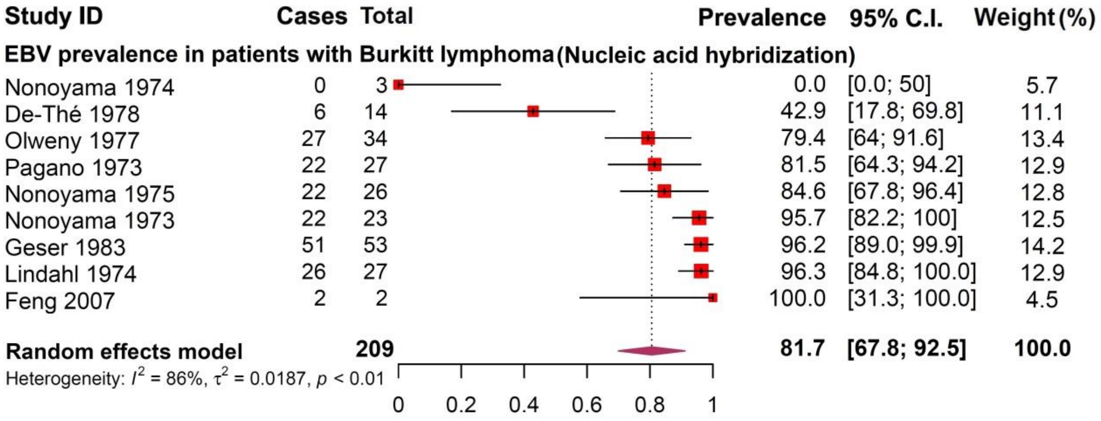
Study ID Cases Total Prevalence 95% C.I. Weight(%)
EBV prevalence in patients with Burkitt lymphoma (Nucleic acid hybridization)
Nonoyama 1974 0 3 0.0 [0.0; 50] 5.7
De-Thé 1978 6 14 42.9 [17.8; 69.8] 11.1
Olweny 1977 27 34 79.4 [64; 91.6] 13.4
Pagano 1973 22 27 81.5 [64.3; 94.2] 12.9
Nonoyama 1975 22 26 84.6 [67.8; 96.4] 12.8
Nonoyama 1973 22 23 95.7 [82.2; 100] 12.5
Geser 1983 51 53 96.2 [89.0; 99.9] 14.2
Lindahl 1974 26 27 96.3 [84.8; 100.0] 12.9
Feng 2007 2 2 100.0 [31.3; 100.0] 4.5
Random effects model 209 81.7 [67.8; 92.5] 100.0
Heterogeneity: I^2 = 86%, τ^2 = 0.0187, p < 0.01
0 0.2 0.4 0.6 0.8 1

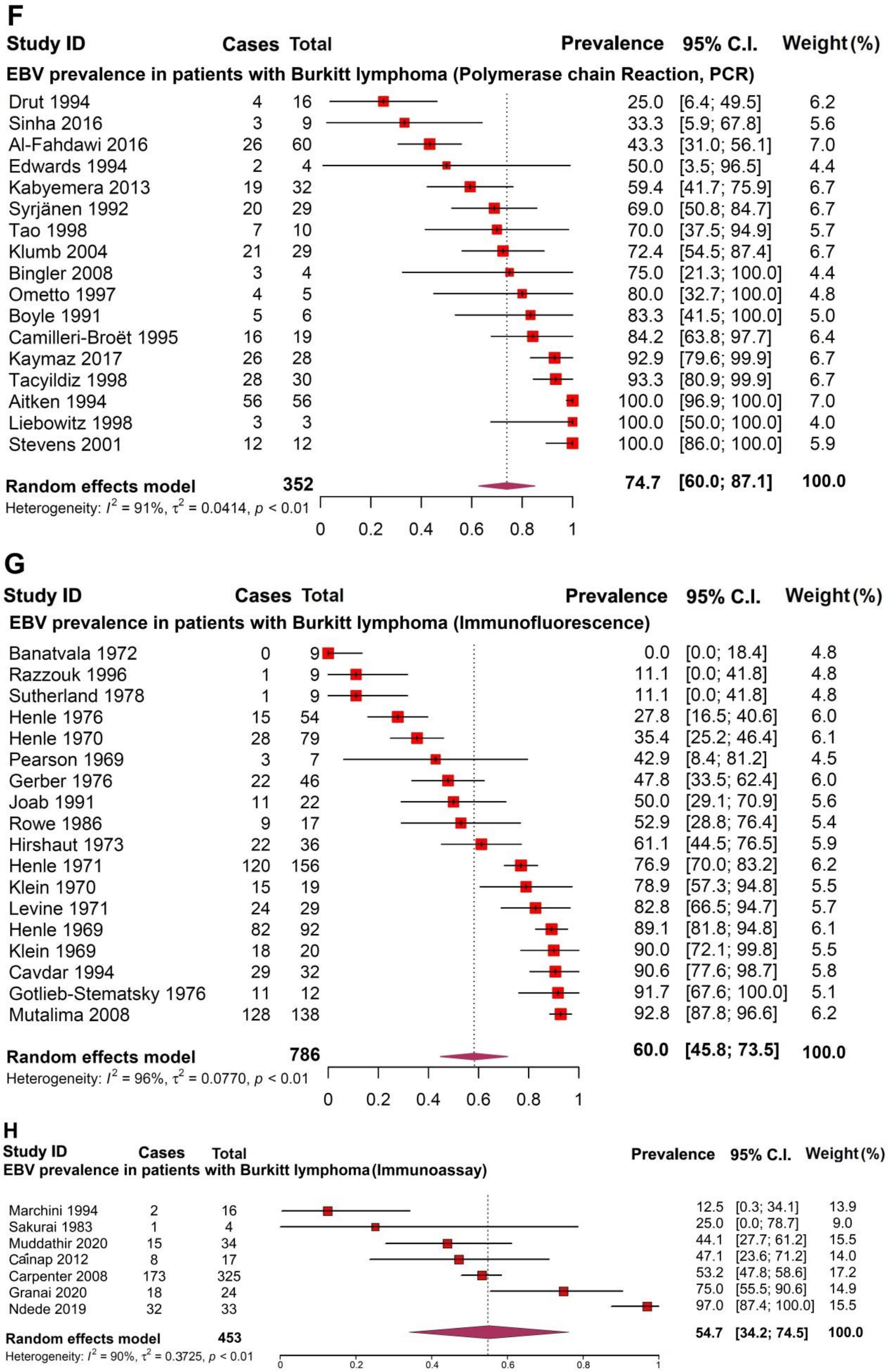
F
Study ID Cases Total Prevalence 95% C.I. Weight (%)
EBV prevalence in patients with Burkitt lymphoma (Polymerase chain Reaction, PCR)
Drut 1994 4 16 25.0 [6.4; 49.5] 6.2
Sinha 2016 3 9 33.3 [5.9; 67.8] 5.6
Al-Fahdawi 2016 26 60 43.3 [31.0; 56.1] 7.0
Edwards 1994 2 4 50.0 [3.5; 96.5] 4.4
Kabyemera 2013 19 32 59.4 [41.7; 75.9] 6.7
Syrjänen 1992 20 29 69.0 [50.8; 84.7] 6.7
Tao 1998 7 10 70.0 [37.5; 94.9] 5.7
Klumb 2004 21 29 72.4 [54.5; 87.4] 6.7
Bingler 2008 3 4 75.0 [21.3; 100.0] 4.4
Ometto 1997 4 5 80.0 [32.7; 100.0] 4.8
Boyle 1991 5 6 83.3 [41.5; 100.0] 5.0
Camilleri-Broët 1995 16 19 84.2 [63.8; 97.7] 6.4
Kaymaz 2017 26 28 92.9 [79.6; 99.9] 6.7
Tacyildiz 1998 28 30 93.3 [80.9; 99.9] 6.7
Aitken 1994 56 56 100.0 [96.9; 100.0] 7.0
Liebowitz 1998 3 3 100.0 [50.0; 100.0] 4.0
Stevens 2001 12 12 100.0 [86.0; 100.0] 5.9
Random effects model 352 74.7 [60.0; 87.1] 100.0
Heterogeneity: I^2 = 91%, τ^2 = 0.0414, p < 0.01
0 0.2 0.4 0.6 0.8 1
G
Study ID Cases Total Prevalence 95% C.I. Weight (%)
EBV prevalence in patients with Burkitt lymphoma (Immunofluorescence)
Banatvala 1972 0 9 0.0 [0.0; 18.4] 4.8
Razzouk 1996 1 9 11.1 [0.0; 41.8] 4.8
Sutherland 1978 1 9 11.1 [0.0; 41.8] 4.8
Henle 1976 15 54 27.8 [16.5; 40.6] 6.0
Henle 1970 28 79 35.4 [25.2; 46.4] 6.1
Pearson 1969 3 7 42.9 [8.4; 81.2] 4.5
Gerber 1976 22 46 47.8 [33.5; 62.4] 6.0
Joab 1991 11 22 50.0 [29.1; 70.9] 5.6
Rowe 1986 9 17 52.9 [28.8; 76.4] 5.4
Hirshaut 1973 22 36 61.1 [44.5; 76.5] 5.9
Henle 1971 120 156 76.9 [70.0; 83.2] 6.2
Klein 1970 15 19 78.9 [57.3; 94.8] 5.5
Levine 1971 24 29 82.8 [66.5; 94.7] 5.7
Henle 1969 82 92 89.1 [81.8; 94.8] 6.1
Klein 1969 18 20 90.0 [72.1; 99.8] 5.5
Cavdar 1994 29 32 90.6 [77.6; 98.7] 5.8
Gotlieb-Stematsky 1976 11 12 91.7 [67.6; 100.0] 5.1
Mutalima 2008 128 138 92.8 [87.8; 96.6] 6.2
Random effects model 786 60.0 [45.8; 73.5] 100.0
Heterogeneity: I^2 = 96%, τ^2 = 0.0770, p < 0.01
0 0.2 0.4 0.6 0.8 1
H
Study ID Cases Total Prevalence 95% C.I. Weight (%)
EBV prevalence in patients with Burkitt lymphoma (Immunoassay)
Marchini 1994 2 16 12.5 [0.3; 34.1] 13.9
Sakurai 1983 1 4 25.0 [0.0; 78.7] 9.0
Muddathir 2020 15 34 44.1 [27.7; 61.2] 15.5
Caînap 2012 8 17 47.1 [23.6; 71.2] 14.0
Carpenter 2008 173 325 53.2 [47.8; 58.6] 17.2
Granai 2020 18 24 75.0 [55.5; 90.6] 14.9
Ndede 2019 32 33 97.0 [87.4; 100.0] 15.5
Random effects model 453 54.7 [34.2; 74.5] 100.0
Heterogeneity: I^2 = 90%, τ^2 = 0.3725, p < 0.01
0 0.2 0.4 0.6 0.8 1

I

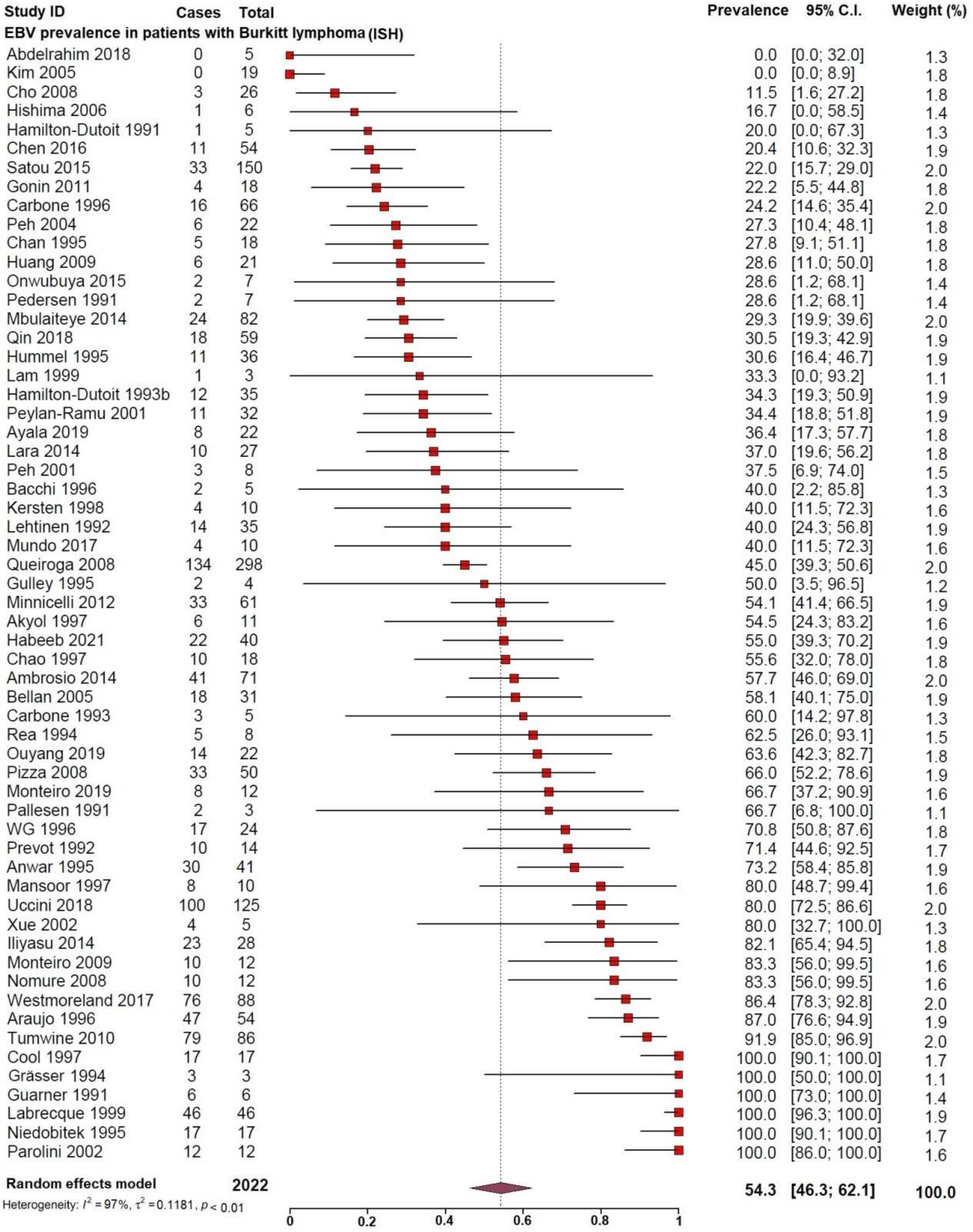
Study ID Cases Total Prevalence 95% C.I. Weight (%)
EBV prevalence in patients with Burkitt lymphoma (ISH)
Abdelrahim 2018 0 5 0.0 [0.0; 32.0] 1.3
Kim 2005 0 19 0.0 [0.0; 8.9] 1.8
Cho 2008 3 26 11.5 [1.6; 27.2] 1.8
Hishima 2006 1 6 16.7 [0.0; 58.5] 1.4
Hamilton-Dutoit 1991 1 5 20.0 [0.0; 67.3] 1.3
Chen 2016 11 54 20.4 [10.6; 32.3] 1.9
Satou 2015 33 150 22.0 [15.7; 29.0] 2.0
Gonin 2011 4 18 22.2 [5.5; 44.8] 1.8
Carbone 1996 16 66 24.2 [14.6; 35.4] 2.0
Peh 2004 6 22 27.3 [10.4; 48.1] 1.8
Chan 1995 5 18 27.8 [9.1; 51.1] 1.8
Huang 2009 6 21 28.6 [11.0; 50.0] 1.8
Onwubuya 2015 2 7 28.6 [1.2; 68.1] 1.4
Pedersen 1991 2 7 28.6 [1.2; 68.1] 1.4
Mbulaiteye 2014 24 82 29.3 [19.9; 39.6] 2.0
Qin 2018 18 59 30.5 [19.3; 42.9] 1.9
Hummel 1995 11 36 30.6 [16.4; 46.7] 1.9
Lam 1999 1 3 33.3 [0.0; 93.2] 1.1
Hamilton-Dutoit 1993b 12 35 34.3 [19.3; 50.9] 1.9
Peylan-Ramu 2001 11 32 34.4 [18.8; 51.8] 1.9
Ayala 2019 8 22 36.4 [17.3; 57.7] 1.8
Lara 2014 10 27 37.0 [19.6; 56.2] 1.8
Peh 2001 3 8 37.5 [6.9; 74.0] 1.5
Bacchi 1996 2 5 40.0 [2.2; 85.8] 1.3
Kersten 1998 4 10 40.0 [11.5; 72.3] 1.6
Lehtinen 1992 14 35 40.0 [24.3; 56.8] 1.9
Mundo 2017 4 10 40.0 [11.5; 72.3] 1.6
Queiroga 2008 134 298 45.0 [39.3; 50.6] 2.0
Gulley 1995 2 4 50.0 [3.5; 96.5] 1.2
Minnicelli 2012 33 61 54.1 [41.4; 66.5] 1.9
Akyol 1997 6 11 54.5 [24.3; 83.2] 1.6
Habeeb 2021 22 40 55.0 [39.3; 70.2] 1.9
Chao 1997 10 18 55.6 [32.0; 78.0] 1.8
Ambrosio 2014 41 71 57.7 [46.0; 69.0] 2.0
Bellan 2005 18 31 58.1 [40.1; 75.0] 1.9
Carbone 1993 3 5 60.0 [14.2; 97.8] 1.3
Rea 1994 5 8 62.5 [26.0; 93.1] 1.5
Ouyang 2019 14 22 63.6 [42.3; 82.7] 1.8
Pizza 2008 33 50 66.0 [52.2; 78.6] 1.9
Monteiro 2019 8 12 66.7 [37.2; 90.9] 1.6
Pallesen 1991 2 3 66.7 [6.8; 100.0] 1.1
WG 1996 17 24 70.8 [50.8; 87.6] 1.8
Prevot 1992 10 14 71.4 [44.6; 92.5] 1.7
Anwar 1995 30 41 73.2 [58.4; 85.8] 1.9
Mansoor 1997 8 10 80.0 [48.7; 99.4] 1.6
Uccini 2018 100 125 80.0 [72.5; 86.6] 2.0
Xue 2002 4 5 80.0 [32.7; 100.0] 1.3
Iliyasu 2014 23 28 82.1 [65.4; 94.5] 1.8
Monteiro 2009 10 12 83.3 [56.0; 99.5] 1.6
Nomure 2008 10 12 83.3 [56.0; 99.5] 1.6
Westmoreland 2017 76 88 86.4 [78.3; 92.8] 2.0
Araujo 1996 47 54 87.0 [76.6; 94.9] 1.9
Tumwine 2010 79 86 91.9 [85.0; 96.9] 2.0
Cool 1997 17 17 100.0 [90.1; 100.0] 1.7
Grässer 1994 3 3 100.0 [50.0; 100.0] 1.1
Guarner 1991 6 6 100.0 [73.0; 100.0] 1.4
Labrecque 1999 46 46 100.0 [96.3; 100.0] 1.9
Niedobitek 1995 17 17 100.0 [90.1; 100.0] 1.7
Parolini 2002 12 12 100.0 [86.0; 100.0] 1.6
Random effects model 2022 54.3 [46.3; 62.1] 100.0
Heterogeneity: I^2 = 97%, τ^2 = 0.1181, p < 0.01
0 0.2 0.4 0.6 0.8 1

J

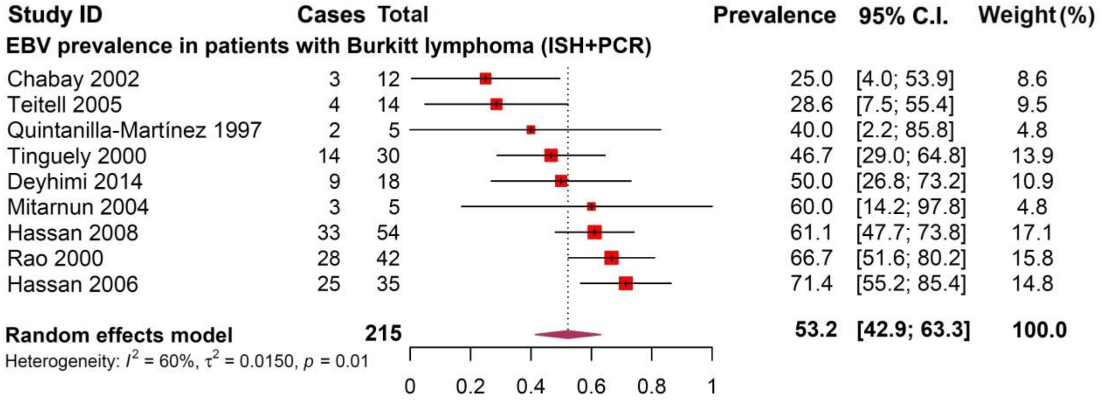
Study ID Cases Total Prevalence 95% C.I. Weight (%)
EBV prevalence in patients with Burkitt lymphoma (ISH+PCR)
Chabay 2002 3 12 25.0 [4.0; 53.9] 8.6
Teitell 2005 4 14 28.6 [7.5; 55.4] 9.5
Quintanilla-Martínez 1997 2 5 40.0 [2.2; 85.8] 4.8
Tinguely 2000 14 30 46.7 [29.0; 64.8] 13.9
Deyhimi 2014 9 18 50.0 [26.8; 73.2] 10.9
Mitarnun 2004 3 5 60.0 [14.2; 97.8] 4.8
Hassan 2008 33 54 61.1 [47.7; 73.8] 17.1
Rao 2000 28 42 66.7 [51.6; 80.2] 15.8
Hassan 2006 25 35 71.4 [55.2; 85.4] 14.8
Random effects model 215 53.2 [42.9; 63.3] 100.0
Heterogeneity: I^2 = 60%, τ^2 = 0.0150, p = 0.01
0 0.2 0.4 0.6 0.8 1

K

EBV prevalence in patients with Burkitt lymphoma (Southern Blot)

Study ID	Cases	Total	Prevalence	95% C.I.	Weight (%)
Lee 1991	0	11	0.0	[0.0; 15.2]	11.7
Subar 1988	6	16	37.5	[15.1; 62.8]	13.4
Gutterrez 1992	20	39	51.3	[35.5; 66.9]	16.5
Okano 1992	4	7	57.1	[18.8; 91.6]	9.6
Hamilton−Dutoit 1993a	11	19	57.9	[34.8; 79.4]	14.1
Barriga 1988	34	56	60.7	[47.5; 73.2]	17.4
Shiramizu 1991	35	54	64.8	[51.5; 77.1]	17.3
**Random effects model**		**202**	**47.1**	**[31.7; 62.8]**	**100.0**

Heterogeneity: $I^2 = 92\%$, $\tau^2 = 0.0807$, $p < 0.01$

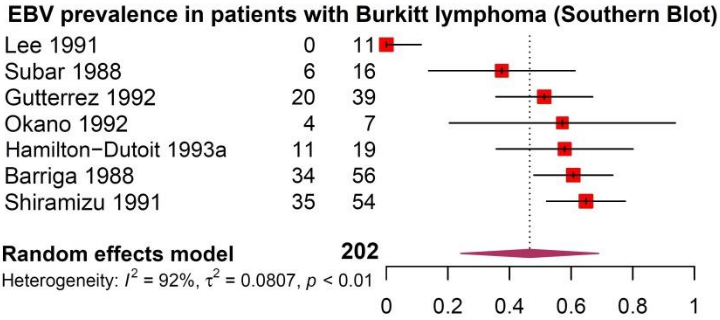

L

EBV prevalence in patients with Burkitt lymphoma (Sub-Saharan Africa)

Study ID	Cases	Total	Prevalence	95% C.I.	Weight (%)
Sutherland 1978	1	9	11.1	[0.0; 41.8]	2.4
Coghill 2020	33	150	22.0	[15.7; 29]	3.1
Henle 1976	15	54	27.8	[16.5; 40.6]	3.0
Onwubuya 2015	2	7	28.6	[1.2; 68.1]	2.3
Henle 1970	28	79	35.4	[25.2; 46.4]	3.1
De-Thé 1978	6	14	42.9	[17.8; 69.8]	2.7
Muddathir 2020	15	34	44.1	[27.7; 61.2]	2.9
Gerber 1976	22	46	47.8	[33.5; 62.4]	3.0
Carpenter 2008	173	325	53.2	[47.8; 58.6]	3.1
Aitken 1994	56	100	56.0	[46.1; 65.6]	3.1
Reedman 1974	11	19	57.9	[34.8; 79.4]	2.8
Tao 1998	7	10	70.0	[37.5; 94.9]	2.5
Prevot 1992	10	14	71.4	[44.6; 92.5]	2.7
Granai 2020	18	24	75.0	[55.5; 90.6]	2.8
Henle 1971	120	156	76.9	[70.0; 83.2]	3.1
Klein 1970	15	19	78.9	[57.3; 94.8]	2.8
Olweny 1977	27	34	79.4	[64.0; 91.6]	2.9
Xue 2002	4	5	80.0	[32.7; 100]	2.1
Iliyasu 2014	23	28	82.1	[65.4; 94.5]	2.9
Nonoyama 1975	22	26	84.6	[67.8; 96.4]	2.9
Westmoreland 2017	76	88	86.4	[78.3; 92.8]	3.1
Henle 1969	82	92	89.1	[81.8; 94.8]	3.1
Klein 1969	18	20	90.0	[72.1; 99.8]	2.8
Tumwine 2010	79	86	91.9	[85.0; 96.9]	3.1
Mutalima 2008	128	138	92.8	[87.8; 96.6]	3.1
Kaymaz 2017	26	28	92.9	[79.6; 99.9]	2.9
Nonoyama 1973	22	23	95.7	[82.2; 100.0]	2.8
Aguilar 2017	260	271	95.9	[93.2; 98.0]	3.1
Geser 1983	51	53	96.2	[89.0; 99.9]	3.0
Lindahl 1974	26	27	96.3	[84.8; 100.0]	2.9
Ndede1 2019	32	33	97.0	[87.4; 100.0]	2.9
Cool 1997	17	17	100.0	[90.1; 100.0]	2.7
Labrecque 1999	46	46	100.0	[96.3; 100.0]	3.0
Niedobitek 1995	17	17	100.0	[90.1; 100.0]	2.7
Stevens 2001	12	12	100.0	[86.0; 100.0]	2.6
**Random effects model**		**2104**	**76.5**	**[67; 84.9]**	**100.0**

Heterogeneity: $I^2 = 77\%$, $\tau^2 = 0.3594$, $p < 0.01$

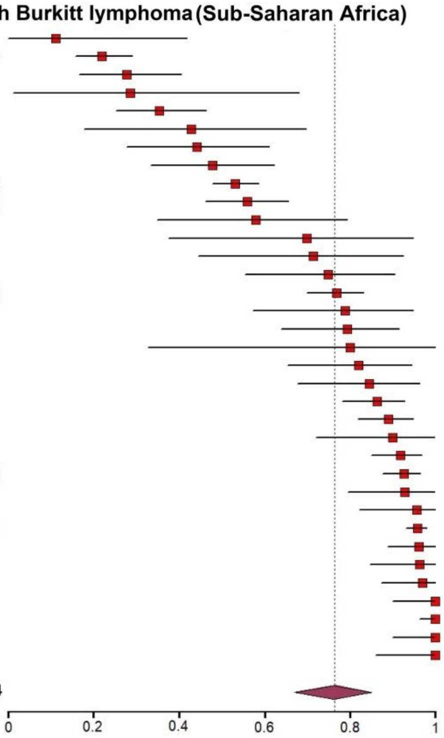

M

EBV prevalence in patients with Burkitt lymphoma (Northern Africa)

Study ID	Cases	Total	Prevalence	95% C.I.	Weight (%)
Peylan-Ramu 2001	11	32	34.4	[18.8; 51.8]	7.40
Al-Fahdawi 2016	26	60	43.3	[31.0; 56.1]	8.0
Tinguely 2000	14	30	46.7	[29.0; 64.8]	7.3
Akyol 1997	6	11	54.5	[24.3; 83.2]	5.7
Habeeb 2021	22	40	55.0	[39.3; 70.2]	7.6
Kabyemera 2013	19	32	59.4	[41.7; 75.9]	7.4
Syrjänen 1992	20	29	69.0	[50.8; 84.7]	7.3
Sulitzeanu 1988	10	14	71.4	[44.6; 92.5]	6.1
Anwar 1995	30	41	73.2	[58.4; 85.8]	7.7
Uccini 2018	100	125	80.0	[72.5; 86.6]	8.4
Cavdar 1994	29	32	90.6	[77.6; 98.7]	7.4
Gotlieb-Stematsky 1976	11	12	91.7	[67.6; 100.0]	5.8
Tacyildiz 1998	28	30	93.3	[80.9; 99.9]	7.3
Cavdar 1993	18	19	94.7	[78.7; 100.0]	6.6
**Random effects model**		**507**	**69.3**	**[58.1; 79.4]**	**100.0**

Heterogeneity: $I^2 = 89\%$, $\tau^2 = 0.0338$, $p < 0.01$

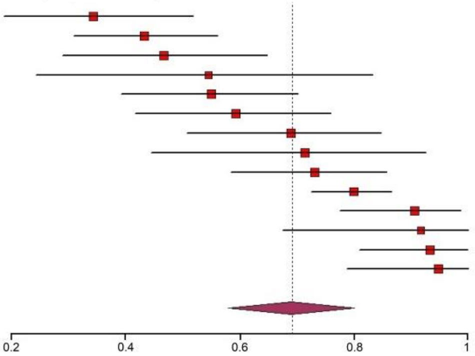

N

EBV prevalence in patients with Burkitt lymphoma (Southern America)

Study ID	Cases	Total	Prevalence	95% C.I.	Weight (%)
Hamilton-Dutoit 1991	1	5	20.0	[0.0; 67.3]	2.7
Chabay 2002	3	12	25.0	[4.0; 53.9]	4.4
Drut 1994	4	16	25.0	[6.4; 49.5]	4.9
Lara 2014	10	27	37.0	[19.6; 56.2]	5.9
Bacchi 1996b	2	5	40.0	[2.2; 85.8]	2.7
Queiroga 2008	134	298	45.0	[39.3; 50.6]	8.0
Gutterrez 1992	20	39	51.3	[35.5; 66.9]	6.5
Minnicelli 2012	33	61	54.1	[41.4; 66.5]	7.0
Hassan 2008	33	54	61.1	[47.7; 73.8]	6.9
Donati 2006	36	58	62.1	[49.2; 74.2]	7.0
Pizza 2008	33	53	62.3	[48.7; 74.9]	6.9
Monteiro 2019	8	12	66.7	[37.2; 90.9]	4.4
WG 1996	17	24	70.8	[50.8; 87.6]	5.7
Hassan 2006	25	35	71.4	[55.2; 85.4]	6.3
Klumb 2004	21	29	72.4	[54.5; 87.4]	6.0
Monteiro 2009	10	12	83.3	[56.0; 99.5]	4.4
Riverend 1984	6	7	85.7	[48.3; 100.0]	3.3
Araujo 1996	47	54	87.0	[76.6; 94.9]	6.9
**Random effects model**		**801**	**58.4**	**[50.0; 66.6]**	**100.0**

Heterogeneity: $I^2 = 84\%$, $\tau^2 = 0.0285$, $p < 0.01$

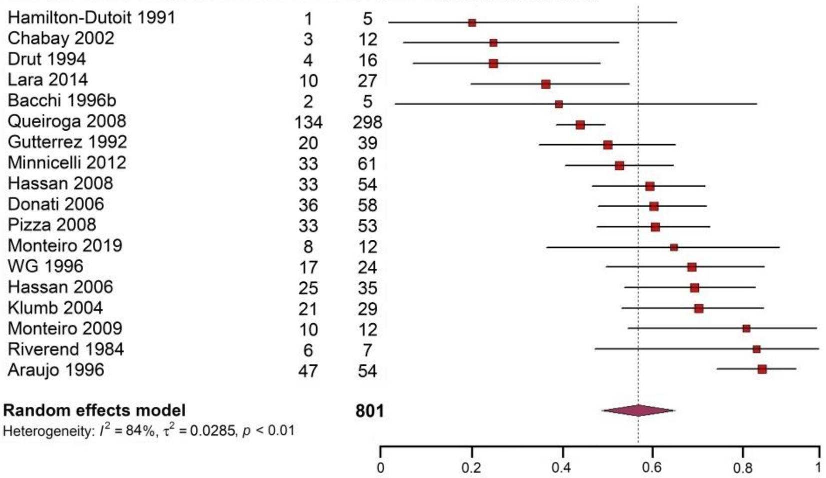

O

EBV prevalence in patients with Burkitt lymphoma (Southern Asia)

Study ID	Cases	Total	Prevalence	95% C.I.	Weight (%)
Sinha 2016	3	9	33.3	[5.9; 67.8]	18.5
Deyhimi 2014	9	18	50.0	[26.8; 73.2]	38.6
Mansoor 1997	8	10	80.0	[48.7; 99.4]	24.4
**Random effects model**		**37**	**54.7**	**[30.5; 77.9]**	**100.0**

Heterogeneity: $I^2 = 66\%$, $\tau^2 = 0.0345$, $p = 0.05$

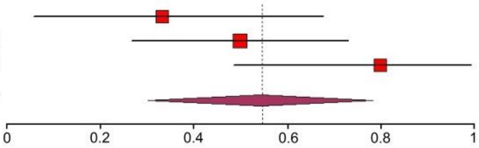

P

EBV prevalence in patients with Burkitt lymphoma (Northern America)

Study ID	Cases	Total	Prevalence	95% C.I.	Weight (%)
Nonoyama 1974	0	3	0.0	[0.0; 50.0]	6.2
Razzouk 1996	1	9	11.1	[0.0; 41.8]	8.7
Teitell 2005	4	14	28.6	[7.5; 55.4]	9.4
Mbulaiteye 2014	24	82	29.3	[19.9; 39.6]	10.9
Subar 1988	6	16	37.5	[15.1; 62.8]	9.6
Edwards 1994	2	4	50.0	[3.5; 96.5]	6.9
Gulley 1995	2	4	50.0	[3.5; 96.5]	6.9
Bingler 2008	3	4	75.0	[21.3; 100.0]	6.9
Pagano 1973	22	27	81.5	[64.3; 94.2]	10.2
Levine 1971	24	29	82.8	[66.5; 94.7]	10.3
Guarner 1991	6	6	100.0	[73.0; 100.0]	7.8
Liebowitz 1998	3	3	100.0	[50.0; 100.0]	6.2
**Random effects model**		**201**	**54.3**	**[34.5; 73.5]**	**100.0**

Heterogeneity: $I^2 = 84\%$, $\tau^2 = 0.3443$, $p < 0.01$

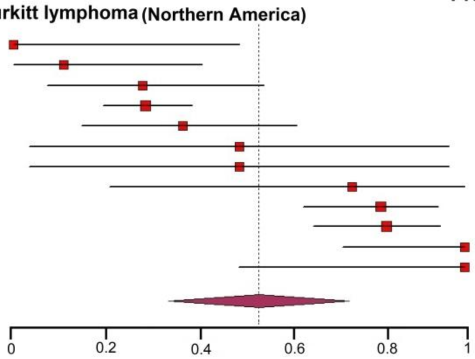

Q

EBV prevalence in patients with Burkitt lymphoma (Europe)

Study ID	Cases	Total	Prevalence	95% C.I.	Weight (%)
Marchini 1994	2	16	12.5	[0.3; 34.1]	6.2
Gonin 2011	4	18	22.2	[5.5; 44.8]	6.3
Carbone 1996	16	66	24.2	[14.6; 35.4]	7.4
Pedersen 1991	2	7	28.6	[1.2; 68.1]	4.9
Hummel 1995	11	36	30.6	[16.4; 46.7]	7.0
Hamilton-Dutoit 1993b	12	35	34.3	[19.3; 50.9]	7.0
Mundo 2017	4	10	40.0	[11.5; 72.3]	5.5
Kersten 1998	4	10	40.0	[11.5; 72.3]	5.5
Pearson 1969	3	7	42.9	[8.4; 81.2]	4.9
Căinap 2012	8	17	47.1	[23.6; 71.2]	6.3
Hamilton-Dutoit 1993a	11	19	57.9	[34.8; 79.4]	6.4
Carbone 1993	3	5	60.0	[14.2; 97.8]	4.3
Rea 1994	5	8	62.5	[26.0; 93.1]	5.1
Pallesen 1991	2	3	66.7	[6.8; 100.0]	3.4
Ometto 1997	4	5	80.0	[32.7; 100.0]	4.3
Camilleri-Broët 1995	16	19	84.2	[63.8; 97.7]	6.4
Parolini 2002	12	12	100.0	[86.0; 100.0]	5.8
Grässer 1994	3	3	100.0	[50.0; 100.0]	3.4
**Random effects model**		**296**	**49.7**	**[36.9; 62.5]**	**100.0**

Heterogeneity: $I^2 = 91\%$, $\tau^2 = 0.1006$, $p < 0.01$

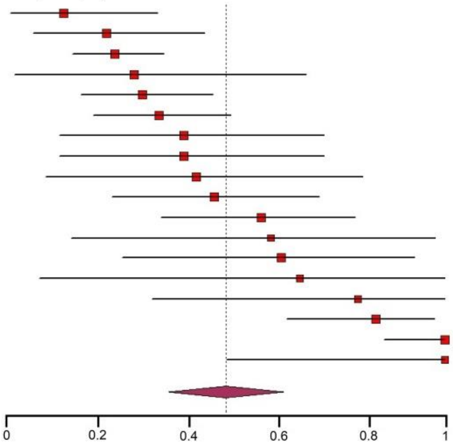

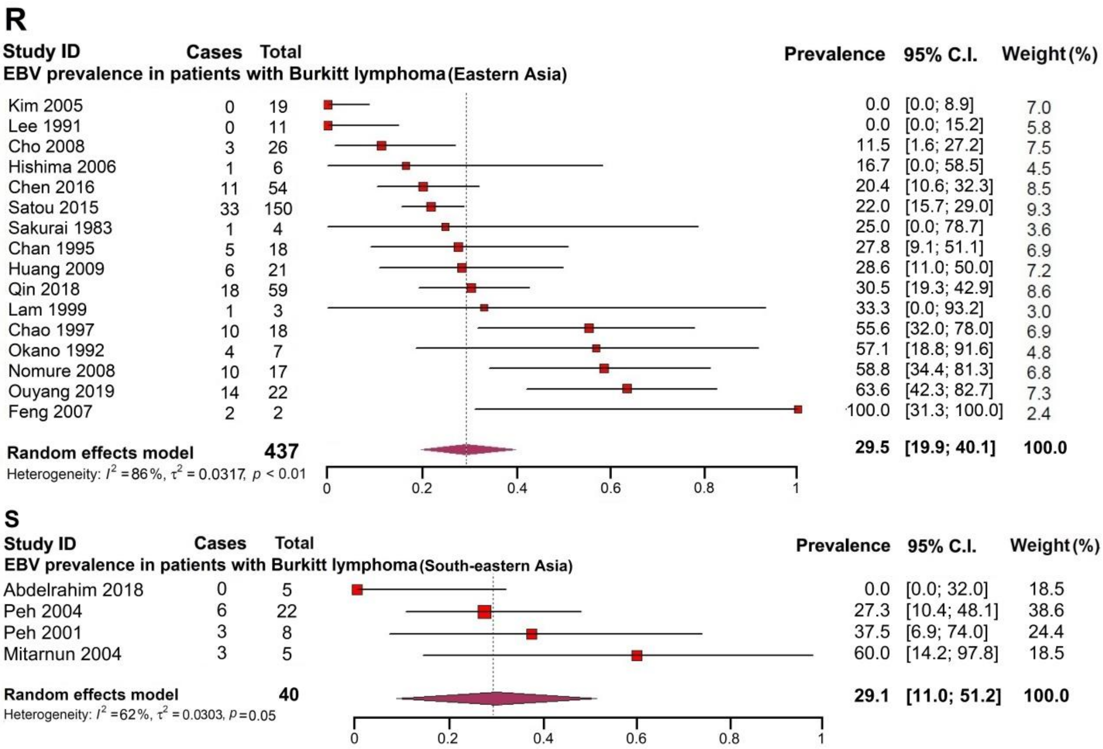
R
Study ID
Cases
Total
Prevalence
95% C.I.
Weight (%)
EBV prevalence in patients with Burkitt lymphoma (Eastern Asia)
Kim 2005 0 19 0.0 [0.0; 8.9] 7.0
Lee 1991 0 11 0.0 [0.0; 15.2] 5.8
Cho 2008 3 26 11.5 [1.6; 27.2] 7.5
Hishima 2006 1 6 16.7 [0.0; 58.5] 4.5
Chen 2016 11 54 20.4 [10.6; 32.3] 8.5
Satou 2015 33 150 22.0 [15.7; 29.0] 9.3
Sakurai 1983 1 4 25.0 [0.0; 78.7] 3.6
Chan 1995 5 18 27.8 [9.1; 51.1] 6.9
Huang 2009 6 21 28.6 [11.0; 50.0] 7.2
Qin 2018 18 59 30.5 [19.3; 42.9] 8.6
Lam 1999 1 3 33.3 [0.0; 93.2] 3.0
Chao 1997 10 18 55.6 [32.0; 78.0] 6.9
Okano 1992 4 7 57.1 [18.8; 91.6] 4.8
Nomure 2008 10 17 58.8 [34.4; 81.3] 6.8
Ouyang 2019 14 22 63.6 [42.3; 82.7] 7.3
Feng 2007 2 2 100.0 [31.3; 100.0] 2.4
Random effects model 437 29.5 [19.9; 40.1] 100.0
Heterogeneity: $I^2 = 86\%$, $\tau^2 = 0.0317$, $p < 0.01$
0
0.2
0.4
0.6
0.8
1
S
Study ID
Cases
Total
Prevalence
95% C.I.
Weight (%)
EBV prevalence in patients with Burkitt lymphoma (South-eastern Asia)
Abdelrahim 2018 0 5 0.0 [0.0; 32.0] 18.5
Peh 2004 6 22 27.3 [10.4; 48.1] 38.6
Peh 2001 3 8 37.5 [6.9; 74.0] 24.4
Mitarnun 2004 3 5 60.0 [14.2; 97.8] 18.5
Random effects model 40 29.1 [11.0; 51.2] 100.0
Heterogeneity: $I^2 = 62\%$, $\tau^2 = 0.0303$, $p = 0.05$
0
0.2
0.4
0.6
0.8
1

T

EBV prevalence in patients with Burkitt lymphoma (High SDI)

Study ID	Cases	Total	Prevalence	95% C.I.	Weight (%)
Kim 2005	0	19	0.0	[0; 8.9]	3.3
Lee 1991	0	11	0.0	[0; 15.2]	2.9
Nonoyama 1974	0	3	0.0	[0; 50]	1.9
Razzouk 1996	1	9	11.1	[0; 41.8]	2.8
Cho 2008	3	26	11.5	[1.6; 27.2]	3.4
Marchini 1994	2	16	12.5	[0.3; 34.1]	3.2
Hishima 2006	1	6	16.7	[0; 58.5]	2.5
Chen 2016	11	54	20.4	[10.6; 32.3]	3.7
Satou 2015	33	150	22.0	[15.7; 29]	3.8
Gonin 2011	4	18	22.2	[5.5; 44.8]	3.3
Sakurai 1983	1	4	25.0	[0; 78.7]	2.1
Pedersen 1991	2	7	28.6	[1.2; 68.1]	2.6
Teitell 2005	4	14	28.6	[7.5; 55.4]	3.1
Mbulaiteye 2014	24	82	29.3	[19.9; 39.6]	3.8
Hummel 1995	11	36	30.6	[16.4; 46.7]	3.6
Hamilton-Dutoit 1993b	12	35	34.3	[19.3; 50.9]	3.5
Subar 1988	6	16	37.5	[15.1; 62.8]	3.2
Kersten 1998	4	10	40.0	[11.5; 72.3]	2.9
Pearson 1969	3	7	42.9	[8.4; 81.2]	2.6
Edwards 1994	2	4	50.0	[3.5; 96.5]	2.1
Gulley 1995	2	4	50.0	[3.5; 96.5]	2.1
Chao 1997	10	18	55.6	[32; 78]	3.3
Okano 1992	4	7	57.1	[18.8; 91.6]	2.6
Hamilton-Dutoit 1993a	11	19	57.9	[34.8; 79.4]	3.3
Rea 1994	5	8	62.5	[26; 93.1]	2.7
Pallesen 1991	2	3	66.7	[6.8; 100]	1.9
Bingler 2008	3	4	75.0	[21.3; 100]	2.1
Pagano 1973	22	27	81.5	[64.3; 94.2]	3.4
Levine 1971	24	29	82.8	[66.5; 94.7]	3.5
Boyle 1991	5	6	83.3	[41.5; 100]	2.5
Nomure 2008	10	12	83.3	[56; 99.5]	3.0
Camilleri-Broët 1995	16	19	84.2	[63.8; 97.7]	3.3
Grässer 1994	3	3	100.0	[50; 100]	1.9
Guarner 1991	6	6	100.0	[73; 100]	2.5
Liebowitz 1998	3	3	100.0	[50; 100]	1.9
**Random effects model**		**695**	**43.0**	**[33.3; 52.9]**	**100.0**

Heterogeneity: $I^2 = 83\%$, $\tau^2 = 0.258$, $p = 0$

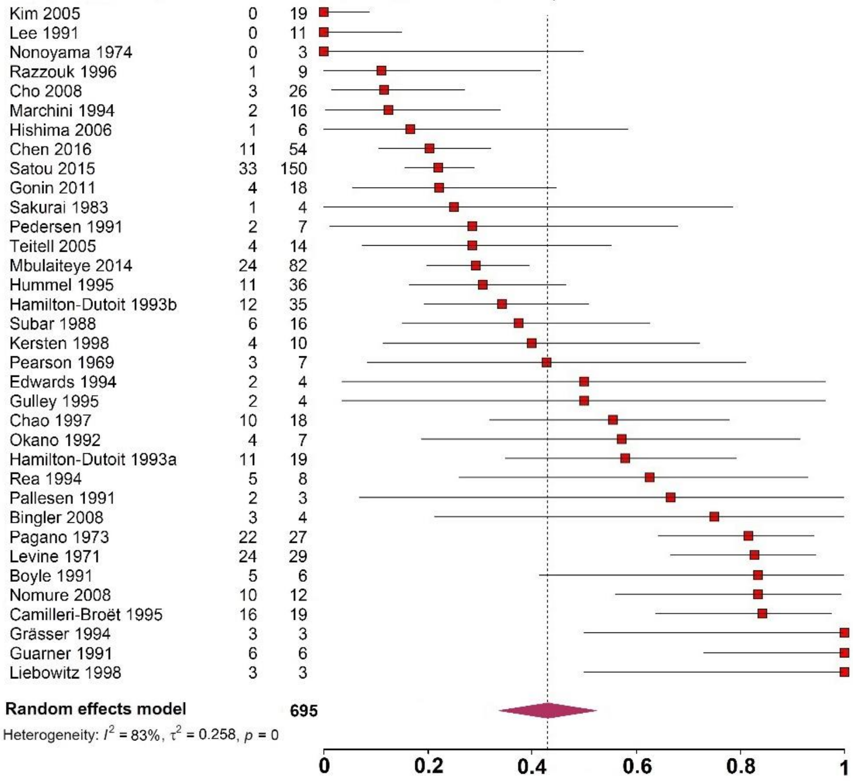

U

EBV prevalence in patients with Burkitt lymphoma (High-middle SDI)

Study ID	Cases	Total	Prevalence	95% C.I.	Weight (%)
Abdelrahim 2018	0	5	0.0	[0.0; 32]	3.8
Hamilton-Dutoit 1991	1	5	20.0	[0.0; 67.3]	3.8
Carbone 1996	16	66	24.2	[14.6; 35.4]	5.5
Chabay 2002	3	12	25.0	[4.0; 53.9]	4.7
Drut 1994	4	16	25.0	[6.4; 49.5]	4.9
Peh 2004	6	22	27.3	[10.4; 48.1]	5.1
Peylan-Ramu 2001	11	32	34.4	[18.8; 51.8]	5.3
Lara 2014	10	27	37.0	[19.6; 56.2]	5.2
Peh 2001	3	8	37.5	[6.9; 74.0]	4.4
Mundo 2017	4	10	40.0	[11.5; 72.3]	4.6
Tinguely 2000	14	30	46.7	[29.0; 64.8]	5.3
Cinap 2012	8	17	47.1	[23.6; 71.2]	5.0
Akyol 1997	6	11	54.5	[24.3; 83.2]	4.7
Carbone 1993	3	5	60.0	[14.2; 97.8]	3.8
Sulitzeanu 1988	10	14	71.4	[44.6; 92.5]	4.8
Ometto 1997	4	5	80.0	[32.7; 100.0]	3.8
Cavdar 1994	29	32	90.6	[77.6; 98.7]	5.3
Gotlieb-Stematsky 1976	11	12	91.7	[67.6; 100.0]	4.7
Tacyildiz 1998	28	30	93.3	[80.9; 99.9]	5.3
Cavdar 1993	18	19	94.7	[78.7; 100.0]	5.0
Parolini 2002	12	12	100.0	[86.0; 100.0]	4.7
**Random effects model**		**390**	**54.5**	**[40; 68.6]**	**100.0**

Heterogeneity: $I^2 = 87\%$, $\tau^2 = 0.366$, $p = 0$

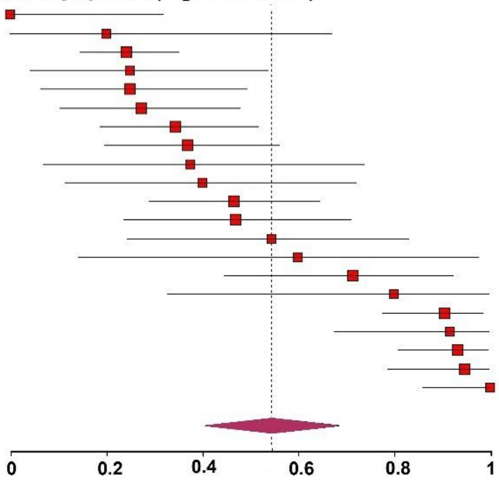

V

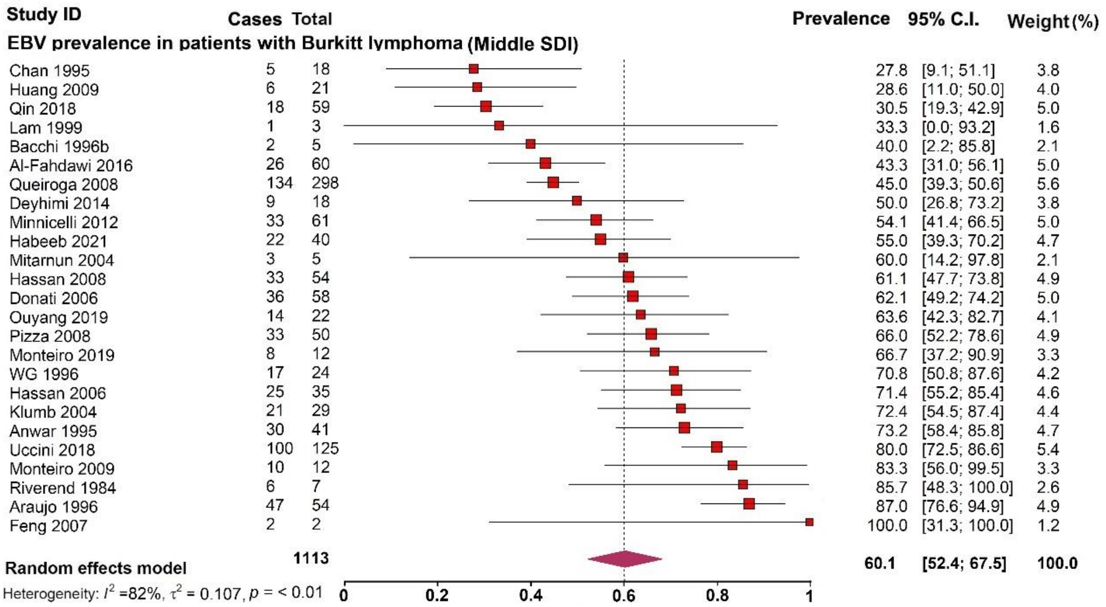

W

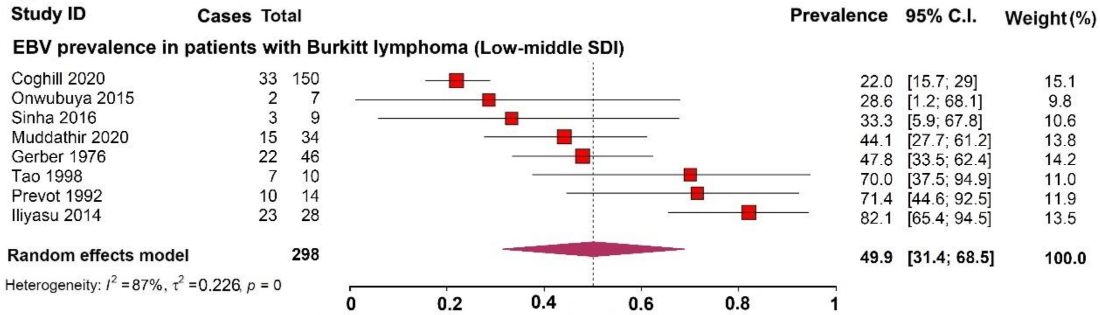

X

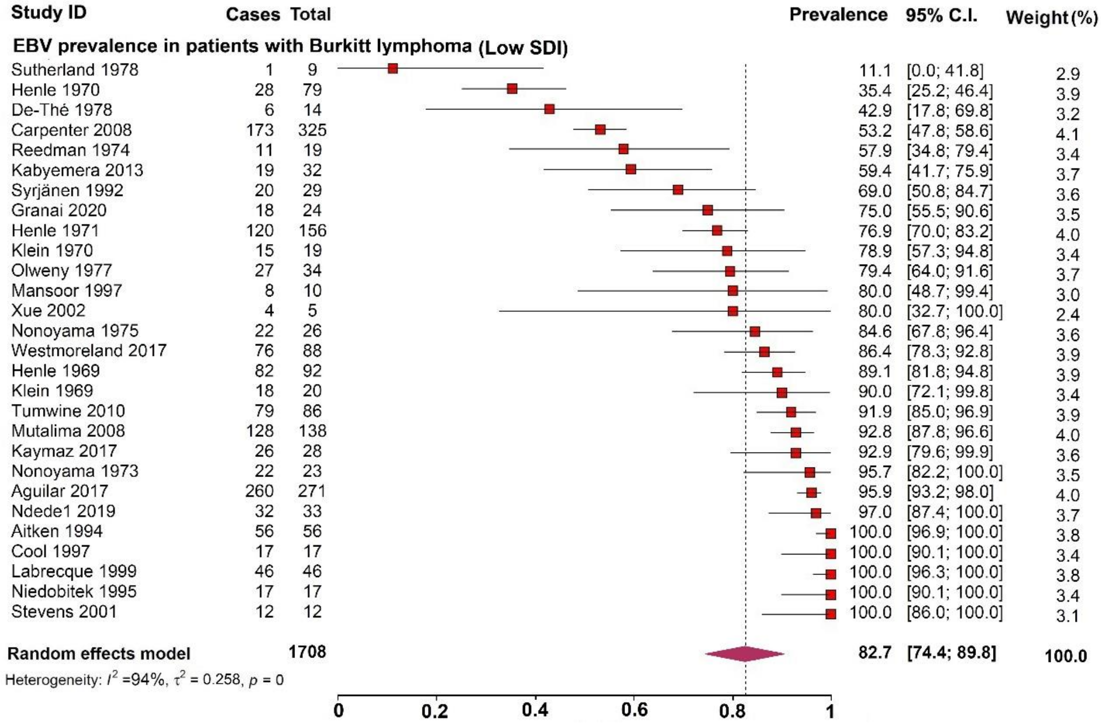

Figure S1. Subgroup analyses estimate the prevalence of the EBV virus at (A-D) different times, using (E-K) different EBV virus detection methods, in (L-S) different regions, and based on the (T-X) Socio-demographic Index (SDI).

A

EBV prevalence in patients with Burkitt lymphoma (considering only studies where age ≥18)

Study ID	Cases	Total	Prevalence	95% C.I.	Weight (%)
Kim 2005	0	19	0.0	[0.0; 8.9]	2.2
Lee 1991	0	11	0.0	[0.0; 15.2]	2.1
Razzouk 1996	1	9	11.1	[0.0; 41.8]	2.0
Coghill 2020	33	150	22.0	[15.7; 29]	2.5
Chabay 2002	3	12	25.0	[4.0; 53.9]	2.1
Drut 1994	4	16	25.0	[6.4; 49.5]	2.2
Peh 2004	6	22	27.3	[10.4; 48.1]	2.3
Teitell 2005	4	14	28.6	[7.5; 55.4]	2.1
Qin 2018	18	59	30.5	[19.3; 42.9]	2.4
Peylan-Ramu 2001	11	32	34.4	[18.8; 51.8]	2.3
Lara 2014	10	27	37.0	[19.6; 56.2]	2.3
De-Thé 1978	6	14	42.9	[17.8; 69.8]	2.1
Muddathir 2020	15	34	44.1	[27.7; 61.2]	2.3
Tinguely 2000	14	30	46.7	[29.0; 64.8]	2.3
Căinap 2012	8	17	47.1	[23.6; 71.2]	2.2
Gerber 1976	22	46	47.8	[33.5; 62.4]	2.4
Carpenter 2008	173	325	53.2	[47.8; 58.6]	2.5
Minnicelli 2012	33	61	54.1	[41.4; 66.5]	2.4
Kabyemera 2013	19	32	59.4	[41.7; 75.9]	2.3
Hassan 2008	33	54	61.1	[47.7; 73.8]	2.4
Donati 2006	36	58	62.1	[49.2; 74.2]	2.4
Pizza 2008	33	50	66.0	[52.2; 78.6]	2.4
WG 1996	17	24	70.8	[50.8; 87.6]	2.3
Klumb 2004	21	29	72.4	[54.5; 87.4]	2.3
Bingler 2008	3	4	75.0	[21.3; 100.0]	1.6
Klein 1970	15	19	78.9	[57.3; 94.8]	2.2
Olweny 1977	27	34	79.4	[64.0; 91.6]	2.3
Uccini 2018	100	125	80.0	[72.5; 86.6]	2.5
Xue 2002	4	5	80.0	[32.7; 100.0]	1.7
Nomure 2008	10	12	83.3	[56; 99.5]	2.1
Riverend 1984	6	7	85.7	[48.3; 100.0]	1.9
Westmoreland 2017	76	88	86.4	[78.3; 92.8]	2.5
Araujo 1996	47	54	87.0	[76.6; 94.9]	2.4
Cavdar 1994	29	32	90.6	[77.6; 98.7]	2.3
Mutalima 2008	128	138	92.8	[87.8; 96.6]	2.5
Kaymaz 2017	26	28	92.9	[79.6; 99.9]	2.3
Tacyildiz 1998	28	30	93.3	[80.9; 99.9]	2.3
Cavdar 1993	18	19	94.7	[78.7; 100.0]	2.2
Aguilar 2017	260	271	95.9	[93.2; 98.0]	2.5
Geser 1983	51	53	96.2	[89.0; 99.9]	2.4
Lindahl 1974	26	27	96.3	[84.8; 100.0]	2.3
Ndede1 2019	32	33	97.0	[87.4; 100.0]	2.3
Labrecque 1999	46	46	100.0	[96.3; 100.0]	2.4
Niedobitek 1995	17	17	100.0	[90.1; 100.0]	2.2
**Random effects model**		**2187**	**64.9**	**[55.4; 74.0]**	**100.0**

Heterogeneity: I^2 = 98%, τ^2 = 0.0787, p = 0

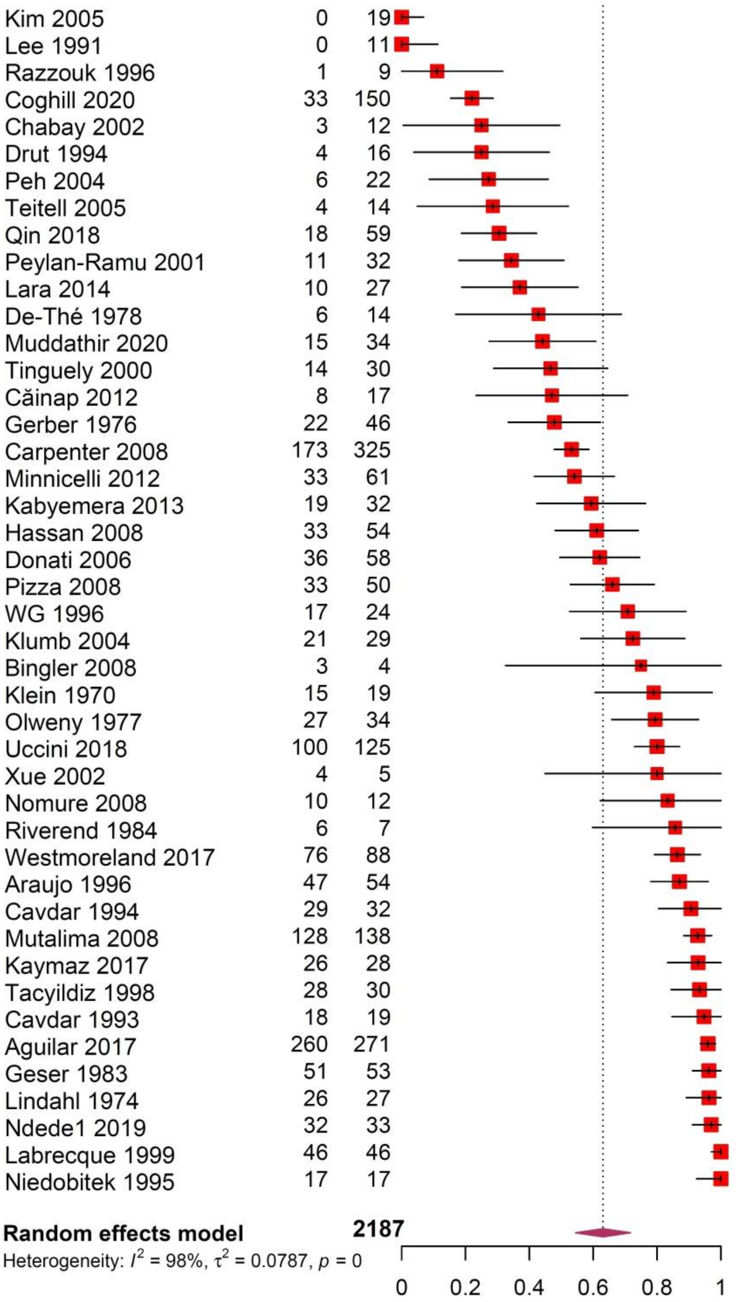

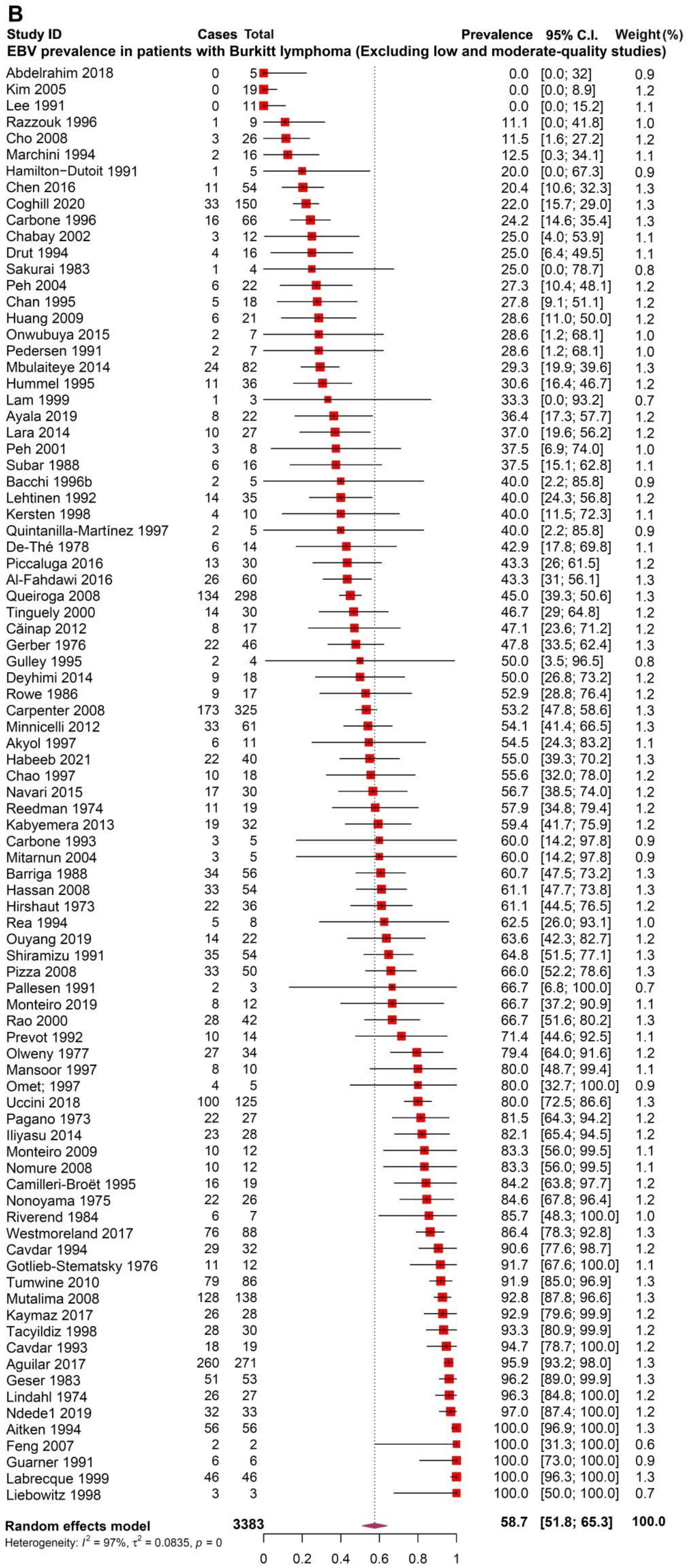
B
Study ID Cases Total Prevalence 95% C.I. Weight (%)
EBV prevalence in patients with Burkitt lymphoma (Excluding low and moderate-quality studies)
Abdelrahim 2018 0 5 0.0 [0.0; 32] 0.9
Kim 2005 0 19 0.0 [0.0; 8.9] 1.2
Lee 1991 0 11 0.0 [0.0; 15.2] 1.1
Razzouk 1996 1 9 11.1 [0.0; 41.8] 1.0
Cho 2008 3 26 11.5 [1.6; 27.2] 1.2
Marchini 1994 2 16 12.5 [0.3; 34.1] 1.1
Hamilton−Dutoit 1991 1 5 20.0 [0.0; 67.3] 0.9
Chen 2016 11 54 20.4 [10.6; 32.3] 1.3
Coghill 2020 33 150 22.0 [15.7; 29.0] 1.3
Carbone 1996 16 66 24.2 [14.6; 35.4] 1.3
Chabay 2002 3 12 25.0 [4.0; 53.9] 1.1
Drut 1994 4 16 25.0 [6.4; 49.5] 1.1
Sakurai 1983 1 4 25.0 [0.0; 78.7] 0.8
Peh 2004 6 22 27.3 [10.4; 48.1] 1.2
Chan 1995 5 18 27.8 [9.1; 51.1] 1.2
Huang 2009 6 21 28.6 [11.0; 50.0] 1.2
Onwubuya 2015 2 7 28.6 [1.2; 68.1] 1.0
Pedersen 1991 2 7 28.6 [1.2; 68.1] 1.0
Mbulaiteye 2014 24 82 29.3 [19.9; 39.6] 1.3
Hummel 1995 11 36 30.6 [16.4; 46.7] 1.2
Lam 1999 1 3 33.3 [0.0; 93.2] 0.7
Ayala 2019 8 22 36.4 [17.3; 57.7] 1.2
Lara 2014 10 27 37.0 [19.6; 56.2] 1.2
Peh 2001 3 8 37.5 [6.9; 74.0] 1.0
Subar 1988 6 16 37.5 [15.1; 62.8] 1.1
Bacchi 1996b 2 5 40.0 [2.2; 85.8] 0.9
Lehtinen 1992 14 35 40.0 [24.3; 56.8] 1.2
Kersten 1998 4 10 40.0 [11.5; 72.3] 1.1
Quintanilla-Martínez 1997 2 5 40.0 [2.2; 85.8] 0.9
De-Thé 1978 6 14 42.9 [17.8; 69.8] 1.1
Piccaluga 2016 13 30 43.3 [26; 61.5] 1.2
Al-Fahdawi 2016 26 60 43.3 [31; 56.1] 1.3
Queiroga 2008 134 298 45.0 [39.3; 50.6] 1.3
Tinguely 2000 14 30 46.7 [29; 64.8] 1.2
Căinap 2012 8 17 47.1 [23.6; 71.2] 1.2
Gerber 1976 22 46 47.8 [33.5; 62.4] 1.3
Gulley 1995 2 4 50.0 [3.5; 96.5] 0.8
Deyhimi 2014 9 18 50.0 [26.8; 73.2] 1.2
Rowe 1986 9 17 52.9 [28.8; 76.4] 1.2
Carpenter 2008 173 325 53.2 [47.8; 58.6] 1.3
Minnicelli 2012 33 61 54.1 [41.4; 66.5] 1.3
Akyol 1997 6 11 54.5 [24.3; 83.2] 1.1
Habeeb 2021 22 40 55.0 [39.3; 70.2] 1.3
Chao 1997 10 18 55.6 [32.0; 78.0] 1.2
Navari 2015 17 30 56.7 [38.5; 74.0] 1.2
Reedman 1974 11 19 57.9 [34.8; 79.4] 1.2
Kabyemera 2013 19 32 59.4 [41.7; 75.9] 1.2
Carbone 1993 3 5 60.0 [14.2; 97.8] 0.9
Mitarnun 2004 3 5 60.0 [14.2; 97.8] 0.9
Barriga 1988 34 56 60.7 [47.5; 73.2] 1.3
Hassan 2008 33 54 61.1 [47.7; 73.8] 1.3
Hirshaut 1973 22 36 61.1 [44.5; 76.5] 1.2
Rea 1994 5 8 62.5 [26.0; 93.1] 1.0
Ouyang 2019 14 22 63.6 [42.3; 82.7] 1.2
Shiramizu 1991 35 54 64.8 [51.5; 77.1] 1.3
Pizza 2008 33 50 66.0 [52.2; 78.6] 1.3
Pallesen 1991 2 3 66.7 [6.8; 100.0] 0.7
Monteiro 2019 8 12 66.7 [37.2; 90.9] 1.1
Rao 2000 28 42 66.7 [51.6; 80.2] 1.3
Prevot 1992 10 14 71.4 [44.6; 92.5] 1.1
Olweny 1977 27 34 79.4 [64.0; 91.6] 1.2
Mansoor 1997 8 10 80.0 [48.7; 99.4] 1.1
Omet; 1997 4 5 80.0 [32.7; 100.0] 0.9
Uccini 2018 100 125 80.0 [72.5; 86.6] 1.3
Pagano 1973 22 27 81.5 [64.3; 94.2] 1.2
Iliyasu 2014 23 28 82.1 [65.4; 94.5] 1.2
Monteiro 2009 10 12 83.3 [56.0; 99.5] 1.1
Nomure 2008 10 12 83.3 [56.0; 99.5] 1.1
Camilleri-Broët 1995 16 19 84.2 [63.8; 97.7] 1.2
Nonoyama 1975 22 26 84.6 [67.8; 96.4] 1.2
Riverend 1984 6 7 85.7 [48.3; 100.0] 1.0
Westmoreland 2017 76 88 86.4 [78.3; 92.8] 1.3
Cavdar 1994 29 32 90.6 [77.6; 98.7] 1.2
Gotlieb-Stematsky 1976 11 12 91.7 [67.6; 100.0] 1.1
Tumwine 2010 79 86 91.9 [85.0; 96.9] 1.3
Mutalima 2008 128 138 92.8 [87.8; 96.6] 1.3
Kaymaz 2017 26 28 92.9 [79.6; 99.9] 1.2
Tacyildiz 1998 28 30 93.3 [80.9; 99.9] 1.2
Cavdar 1993 18 19 94.7 [78.7; 100.0] 1.2
Aguilar 2017 260 271 95.9 [93.2; 98.0] 1.3
Geser 1983 51 53 96.2 [89.0; 99.9] 1.3
Lindahl 1974 26 27 96.3 [84.8; 100.0] 1.2
Ndede1 2019 32 33 97.0 [87.4; 100.0] 1.2
Aitken 1994 56 56 100.0 [96.9; 100.0] 1.3
Feng 2007 2 2 100.0 [31.3; 100.0] 0.6
Guarner 1991 6 6 100.0 [73.0; 100.0] 0.9
Labrecque 1999 46 46 100.0 [96.3; 100.0] 1.3
Liebowitz 1998 3 3 100.0 [50.0; 100.0] 0.7
Random effects model 3383 58.7 [51.8; 65.3] 100.0
Heterogeneity: I^2 = 97%, τ^2 = 0.0835, p = 0
0 0.2 0.4 0.6 0.8 1

C

EBV prevalence in patients with Burkitt lymphoma (considering only cross-sectional studies)

Study ID	Cases	Total	Prevalence	95% C.I.	Weight (%)
Abdelrahim 2018	0	5	0.0	[0.0; 32.0]	1.0
Kim 2005	0	19	0.0	[0.0; 8.9]	1.3
Lee 1991	0	11	0.0	[0; 15.2]	1.2
Nonoyama 1974	0	3	0.0	[0; 50.0]	0.9
Razzouk 1996	1	9	11.1	[0.0; 41.8]	1.2
Cho 2008	3	26	11.5	[1.6; 27.2]	1.4
Marchini 1994	2	16	12.5	[0.3; 34.1]	1.3
Hamilton-Dutoit 1991	1	5	20.0	[0.0; 67.3]	1.0
Chen 2016	11	54	20.4	[10.6; 32.3]	1.5
Carbone 1996	16	66	24.2	[14.6; 35.4]	1.5
Chabay 2002	3	12	25.0	[4.0; 53.9]	1.3
Drut 1994	4	16	25.0	[6.4; 49.5]	1.3
Sakurai 1983	1	4	25.0	[0.0; 78.7]	1.0
Peh 2004	6	22	27.3	[10.4; 48.1]	1.4
Chan 1995	5	18	27.8	[9.1; 51.1]	1.3
Huang 2009	6	21	28.6	[11.0; 50]	1.4
Onwubuya 2015	2	7	28.6	[1.2; 68.1]	1.1
Teitell 2005	4	14	28.6	[7.5; 55.4]	1.3
Mbulaiteye 2014	24	82	29.3	[19.9; 39.6]	1.5
Hummel 1995	11	36	30.6	[16.4; 46.7]	1.4
Lam 1999	1	3	33.3	[0.0; 93.2]	0.9
Hamilton-Dutoit 1993b	12	35	34.3	[19.3; 50.9]	1.4
Ayala 2019	8	22	36.4	[17.3; 57.7]	1.4
Lara 2014	10	27	37.0	[19.6; 56.2]	1.4
Peh 2001	3	8	37.5	[6.9; 74.0]	1.2
Subar 1988	6	16	37.5	[15.1; 62.8]	1.3
Bacchi 1996	2	5	40.0	[2.2; 85.8]	1.0
Kersten 1998	4	10	40.0	[11.5; 72.3]	1.2
Lehtinen 1992	14	35	40.0	[24.3; 56.8]	1.4
Quintanilla-Martínez 1997	2	5	40.0	[2.2; 85.8]	1.0
Piccaluga 2016	13	30	43.3	[26.0; 61.5]	1.4
Queiroga 2008	134	298	45.0	[39.3; 50.6]	1.5
Deyhimi 2014	9	18	50.0	[26.8; 73.2]	1.3
Edwards 1994	2	4	50.0	[3.5; 96.5]	1.0
Gulley 1995	2	4	50.0	[3.5; 96.5]	1.0
Gutterrez 1992	20	39	51.3	[35.5; 66.9]	1.4
Rowe 1986	9	17	52.9	[28.8; 76.4]	1.3
Akyol 1997	6	11	54.5	[24.3; 83.2]	1.2
Chao 1997	10	18	55.6	[32.0; 78.0]	1.3
Navari 2015	17	30	56.7	[38.5; 74.0]	1.4
Hamilton-Dutoit 1993a	11	19	57.9	[34.8; 79.4]	1.3
Reedman 1974	11	19	57.9	[34.8; 79.4]	1.3
Carbone 1993	3	5	60.0	[14.2; 97.8]	1.0
Mitarnun 2004	3	5	60.0	[14.2; 97.8]	1.0
Barriga 1988	34	56	60.7	[47.5; 73.2]	1.5
Hassan 2008	33	54	61.1	[47.7; 73.8]	1.5
Rea 1994	5	8	62.5	[26.0; 93.1]	1.2
Ouyang 2019	14	22	63.6	[42.3; 82.7]	1.4
Shiramizu 1991	35	54	64.8	[51.5; 77.1]	1.5
Monteiro 2019	8	12	66.7	[37.2; 90.9]	1.3
Pallesen 1991	2	3	66.7	[6.8; 100.0]	0.9
Rao 2000	28	42	66.7	[51.6; 80.2]	1.4
Syrjänen 1992	20	29	69.0	[50.8; 84.7]	1.4
Tao 1998	7	10	70.0	[37.5; 94.9]	1.2
Prevot 1992	10	14	71.4	[44.6; 92.5]	1.3
Olweny 1977	27	34	79.4	[64.0; 91.6]	1.4
Mansoor 1997	8	10	80.0	[48.7; 99.4]	1.2
Ometto 1997	4	5	80.0	[32.7; 100.0]	1.0
Uccini 2018	100	125	80.0	[72.5; 86.6]	1.5
Pagano 1973	22	27	81.5	[64.3; 94.2]	1.4
Iliyasu 2014	23	28	82.1	[65.4; 94.5]	1.4
Monteiro 2009	10	12	83.3	[56.0; 99.5]	1.3
Nomure 2008	10	12	83.3	[56.0; 99.5]	1.3
Camilleri-Broët 1995	16	19	84.2	[63.8; 97.7]	1.3
Nonoyama 1975	22	26	84.6	[67.8; 96.4]	1.4
Tumwine 2010	79	86	91.9	[85.0; 96.9]	1.5
Kaymaz 2017	26	28	92.9	[79.6; 99.9]	1.4
Tacyildiz 1998	28	30	93.3	[80.9; 99.9]	1.4
Nonoyama 1973	22	23	95.7	[82.2; 100.0]	1.4
Geser 1983	51	53	96.2	[89.0; 99.9]	1.5
Lindahl 1974	26	27	96.3	[84.8; 100.0]	1.4
Ndede1 2019	32	33	97.0	[87.4; 100.0]	1.4
Aitken 1994	56	56	100.0	[96.9; 100.0]	1.5
Cool 1997	17	17	100.0	[90.1; 100.0]	1.3
Feng 2007	2	2	100.0	[31.3; 100.0]	0.7
Grässer 1994	3	3	100.0	[50.0; 100.0]	0.9
Guarner 1991	6	6	100.0	[73.0; 100.0]	1.1
Labrecque 1999	46	46	100.0	[96.3; 100.0]	1.4
Liebowitz 1998	3	3	100.0	[50.0; 100.0]	0.9
**Random effects model**		**2144**	**57.4**	**[50.1; 64.6]**	**100.0**

Heterogeneity: $I^2 = 97\%$, $\tau^2 = 0.0996$, $p = 0$

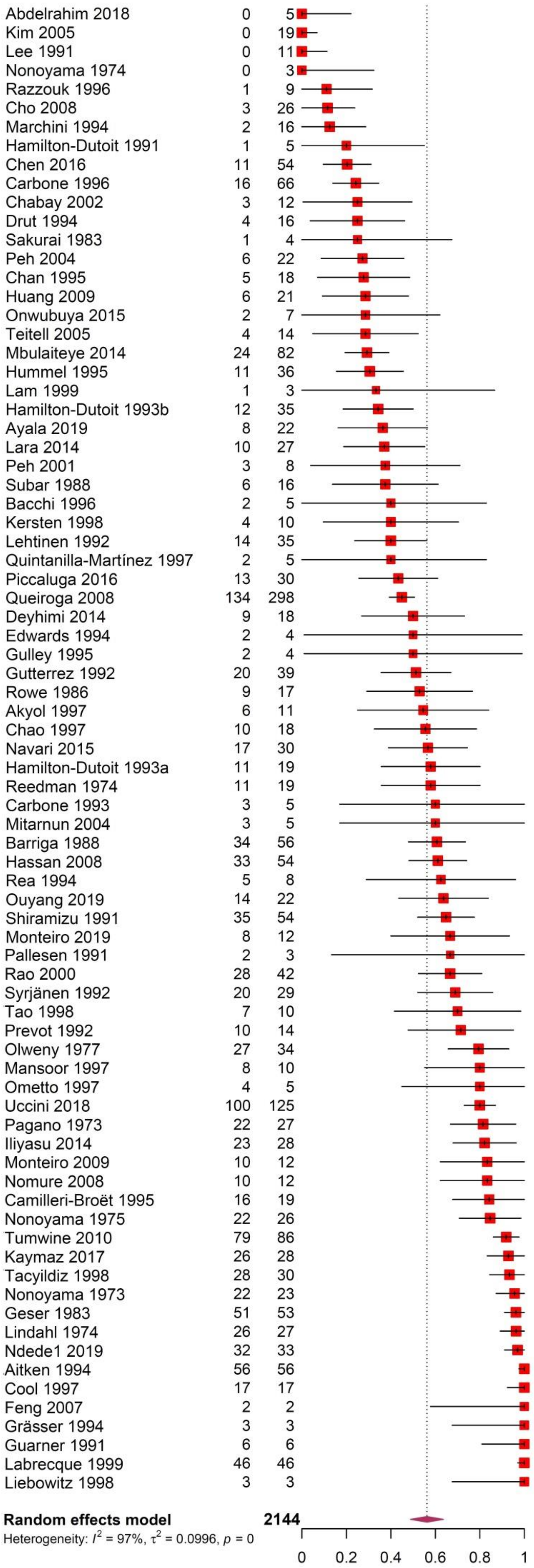

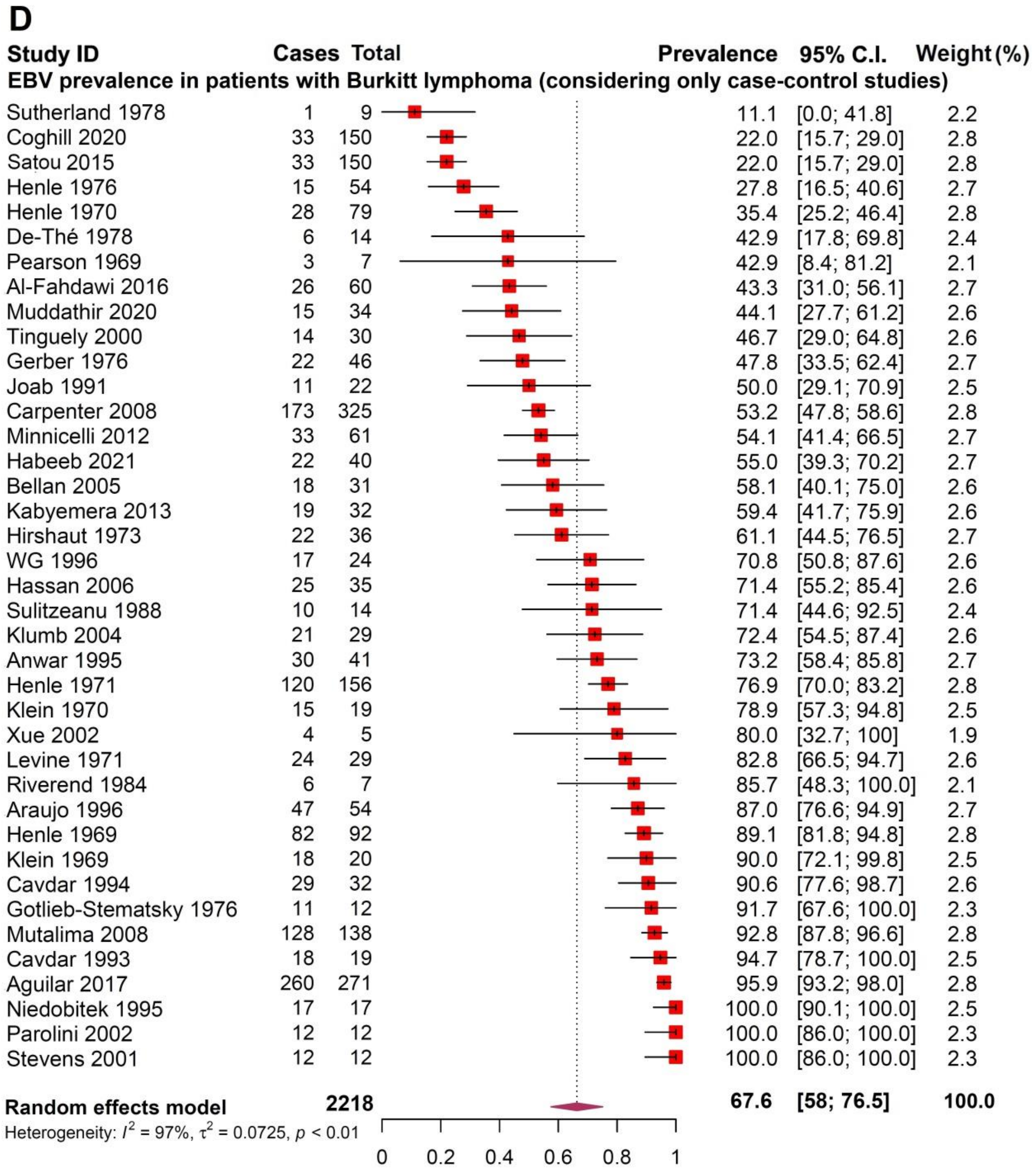
D
Study ID Cases Total Prevalence 95% C.I. Weight (%)
EBV prevalence in patients with Burkitt lymphoma (considering only case-control studies)
Sutherland 1978 1 9 11.1 [0.0; 41.8] 2.2
Coghill 2020 33 150 22.0 [15.7; 29.0] 2.8
Satou 2015 33 150 22.0 [15.7; 29.0] 2.8
Henle 1976 15 54 27.8 [16.5; 40.6] 2.7
Henle 1970 28 79 35.4 [25.2; 46.4] 2.8
De-Thé 1978 6 14 42.9 [17.8; 69.8] 2.4
Pearson 1969 3 7 42.9 [8.4; 81.2] 2.1
Al-Fahdawi 2016 26 60 43.3 [31.0; 56.1] 2.7
Muddathir 2020 15 34 44.1 [27.7; 61.2] 2.6
Tinguely 2000 14 30 46.7 [29.0; 64.8] 2.6
Gerber 1976 22 46 47.8 [33.5; 62.4] 2.7
Joab 1991 11 22 50.0 [29.1; 70.9] 2.5
Carpenter 2008 173 325 53.2 [47.8; 58.6] 2.8
Minnicelli 2012 33 61 54.1 [41.4; 66.5] 2.7
Habeeb 2021 22 40 55.0 [39.3; 70.2] 2.7
Bellan 2005 18 31 58.1 [40.1; 75.0] 2.6
Kabyemera 2013 19 32 59.4 [41.7; 75.9] 2.6
Hirshaut 1973 22 36 61.1 [44.5; 76.5] 2.7
WG 1996 17 24 70.8 [50.8; 87.6] 2.6
Hassan 2006 25 35 71.4 [55.2; 85.4] 2.6
Sulitzeanu 1988 10 14 71.4 [44.6; 92.5] 2.4
Klumb 2004 21 29 72.4 [54.5; 87.4] 2.6
Anwar 1995 30 41 73.2 [58.4; 85.8] 2.7
Henle 1971 120 156 76.9 [70.0; 83.2] 2.8
Klein 1970 15 19 78.9 [57.3; 94.8] 2.5
Xue 2002 4 5 80.0 [32.7; 100] 1.9
Levine 1971 24 29 82.8 [66.5; 94.7] 2.6
Riverend 1984 6 7 85.7 [48.3; 100.0] 2.1
Araujo 1996 47 54 87.0 [76.6; 94.9] 2.7
Henle 1969 82 92 89.1 [81.8; 94.8] 2.8
Klein 1969 18 20 90.0 [72.1; 99.8] 2.5
Cavdar 1994 29 32 90.6 [77.6; 98.7] 2.6
Gotlieb-Stematsky 1976 11 12 91.7 [67.6; 100.0] 2.3
Mutalima 2008 128 138 92.8 [87.8; 96.6] 2.8
Cavdar 1993 18 19 94.7 [78.7; 100.0] 2.5
Aguilar 2017 260 271 95.9 [93.2; 98.0] 2.8
Niedobitek 1995 17 17 100.0 [90.1; 100.0] 2.5
Parolini 2002 12 12 100.0 [86.0; 100.0] 2.3
Stevens 2001 12 12 100.0 [86.0; 100.0] 2.3
Random effects model 2218 67.6 [58; 76.5] 100.0
Heterogeneity: I^2 = 97%, τ^2 = 0.0725, p < 0.01
0 0.2 0.4 0.6 0.8 1

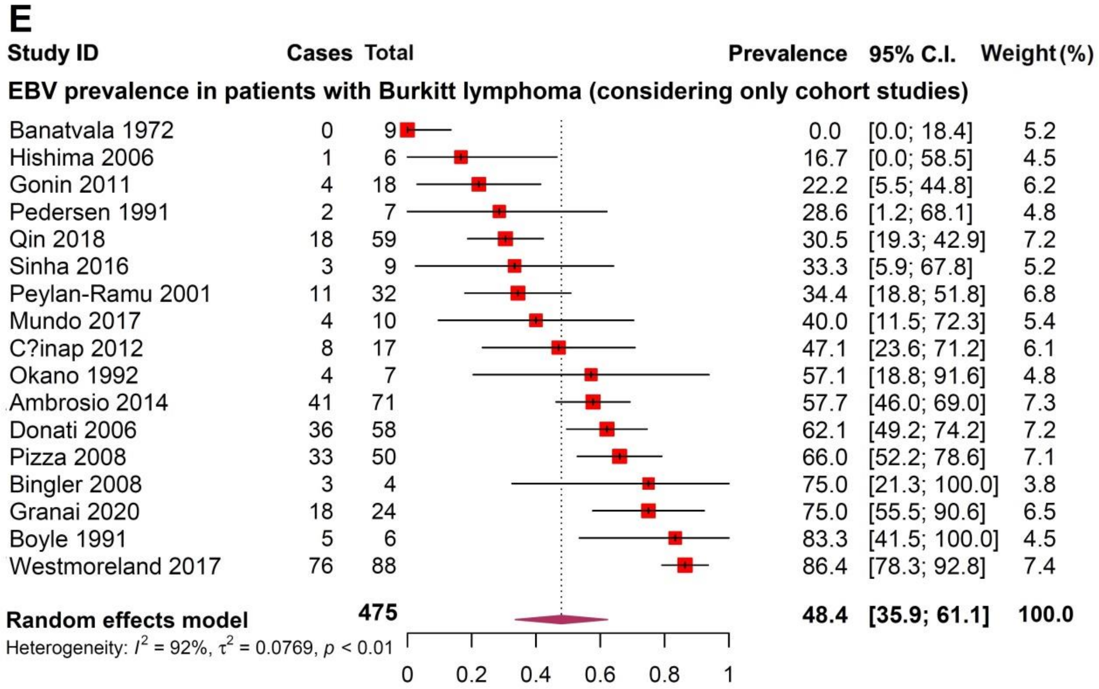
E
Study ID Cases Total Prevalence 95% C.I. Weight (%)
EBV prevalence in patients with Burkitt lymphoma (considering only cohort studies)
Banatvala 1972 0 9 0.0 [0.0; 18.4] 5.2
Hishima 2006 1 6 16.7 [0.0; 58.5] 4.5
Gonin 2011 4 18 22.2 [5.5; 44.8] 6.2
Pedersen 1991 2 7 28.6 [1.2; 68.1] 4.8
Qin 2018 18 59 30.5 [19.3; 42.9] 7.2
Sinha 2016 3 9 33.3 [5.9; 67.8] 5.2
Peylan-Ramu 2001 11 32 34.4 [18.8; 51.8] 6.8
Mundo 2017 4 10 40.0 [11.5; 72.3] 5.4
C?inap 2012 8 17 47.1 [23.6; 71.2] 6.1
Okano 1992 4 7 57.1 [18.8; 91.6] 4.8
Ambrosio 2014 41 71 57.7 [46.0; 69.0] 7.3
Donati 2006 36 58 62.1 [49.2; 74.2] 7.2
Pizza 2008 33 50 66.0 [52.2; 78.6] 7.1
Bingler 2008 3 4 75.0 [21.3; 100.0] 3.8
Granai 2020 18 24 75.0 [55.5; 90.6] 6.5
Boyle 1991 5 6 83.3 [41.5; 100.0] 4.5
Westmoreland 2017 76 88 86.4 [78.3; 92.8] 7.4
Random effects model 475 48.4 [35.9; 61.1] 100.0
Heterogeneity: I^2 = 92%, τ^2 = 0.0769, p < 0.01
0 0.2 0.4 0.6 0.8 1

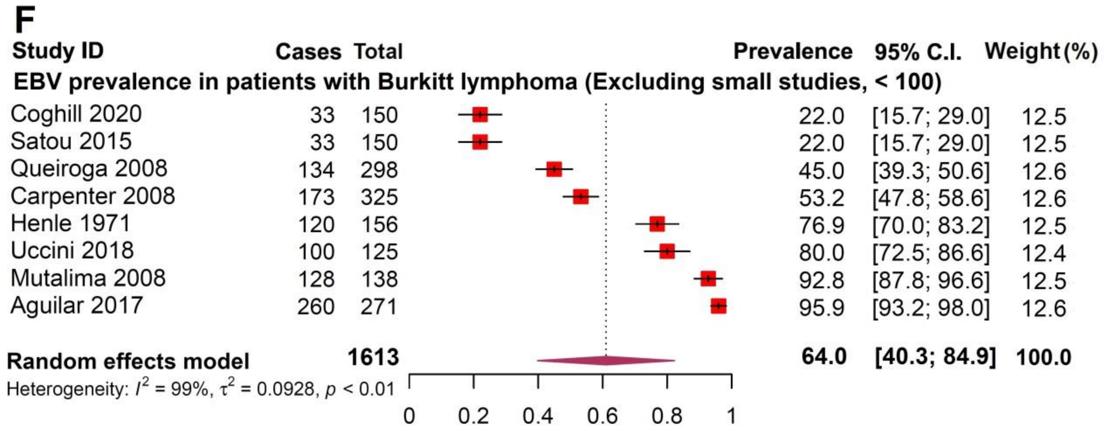
F
Study ID Cases Total Prevalence 95% C.I. Weight (%)
EBV prevalence in patients with Burkitt lymphoma (Excluding small studies, < 100)
Coghill 2020 33 150 22.0 [15.7; 29.0] 12.5
Satou 2015 33 150 22.0 [15.7; 29.0] 12.5
Queiroga 2008 134 298 45.0 [39.3; 50.6] 12.6
Carpenter 2008 173 325 53.2 [47.8; 58.6] 12.6
Henle 1971 120 156 76.9 [70.0; 83.2] 12.5
Uccini 2018 100 125 80.0 [72.5; 86.6] 12.4
Mutalima 2008 128 138 92.8 [87.8; 96.6] 12.5
Aguilar 2017 260 271 95.9 [93.2; 98.0] 12.6
Random effects model 1613 64.0 [40.3; 84.9] 100.0
Heterogeneity: I^2 = 99%, τ^2 = 0.0928, p < 0.01
0 0.2 0.4 0.6 0.8 1

G

EBV prevalence in patients with Burkitt lymphoma (Excluding outlier studies)

Study ID	Cases	Total	Prevalence	95% C.I.	Weight (%)
Abdelrahim 2018	0	5	0.0	[0.0; 32.0]	0.6
Nonoyama 1974	0	3	0.0	[0.0; 50.0]	0.5
Razzouk 1996	1	9	11.1	[0.0; 41.8]	0.7
Sutherland 1978	1	9	11.1	[0.0; 41.8]	0.7
Cho 2008	3	26	11.5	[1.6; 27.2]	0.8
Marchini 1994	2	16	12.5	[0.3; 34.1]	0.7
Hishima 2006	1	6	16.7	[0.0; 58.5]	0.6
Hamilton-Dutoit 1991	1	5	20.0	[0.0; 67.3]	0.6
Chen 2016	11	54	20.4	[10.6; 32.3]	0.8
Coghill 2020	33	150	22.0	[15.7; 29]	0.9
Satou 2015	33	150	22.0	[15.7; 29]	0.9
Gonin 2011	4	18	22.2	[5.5; 44.8]	0.8
Carbone 1996	16	66	24.2	[14.6; 35.4]	0.8
Chabay 2002	3	12	25.0	[4.0; 53.9]	0.7
Drut 1994	4	16	25.0	[6.4; 49.5]	0.7
Sakurai 1983	1	4	25.0	[0.0; 78.7]	0.5
Peh 2004	6	22	27.3	[10.4; 48.1]	0.8
Chan 1995	5	18	27.8	[9.1; 51.1]	0.8
Henle 1976	15	54	27.8	[16.5; 40.6]	0.8
Huang 2009	6	21	28.6	[11.0; 50]	0.8
Onwubuya 2015	2	7	28.6	[1.2; 68.1]	0.6
Teitell 2005	4	14	28.6	[7.5; 55.4]	0.7
Pedersen 1991	2	7	28.6	[1.2; 68.1]	0.6
Mbulaiteye 2014	24	82	29.3	[19.9; 39.6]	0.9
Qin 2018	18	59	30.5	[19.3; 42.9]	0.8
Hummel 1995	11	36	30.6	[16.4; 46.7]	0.8
Lam 1999	1	3	33.3	[0.0; 93.2]	0.5
Sinha 2016	3	9	33.3	[5.9; 67.8]	0.7
Hamilton-Dutoit 1993b	12	35	34.3	[19.3; 50.9]	0.8
Peylan-Ramu 2001	11	32	34.4	[18.8; 51.8]	0.8
Henle 1970	28	79	35.4	[25.2; 46.4]	0.8
Ayala 2019	8	22	36.4	[17.3; 57.7]	0.8
Lara 2014	10	27	37.0	[19.6; 56.2]	0.8
Peh 2001	3	8	37.5	[6.9; 74.0]	0.7
Subar 1988	6	16	37.5	[15.1; 62.8]	0.7
Bacchi 1996	2	5	40.0	[2.2; 85.8]	0.6
Kersten 1998	4	10	40.0	[11.5; 72.3]	0.7
Lehtinen 1992	14	35	40.0	[24.3; 56.8]	0.8
Quintanilla-Martínez 1997	2	5	40.0	[2.2; 85.8]	0.6
Mundo 2017	4	10	40.0	[11.5; 72.3]	0.7
De-Thé 1978	6	14	42.9	[17.8; 69.8]	0.7
Pearson 1969	3	7	42.9	[8.4; 81.2]	0.6
Piccaluga 2016	13	30	43.3	[26.0; 61.5]	0.8
Al-Fahdawi 2016	26	60	43.3	[31.0; 56.1]	0.8
Muddathir 2020	15	34	44.1	[27.7; 61.2]	0.8
Queiroga 2008	134	298	45.0	[39.3; 50.6]	0.9
Tinguely 2000	14	30	46.7	[29.0; 64.8]	0.8
Căinap 2012	8	17	47.1	[23.6; 71.2]	0.8
Gerber 1976	22	46	47.8	[33.5; 62.4]	0.8
Deyhimi 2014	9	18	50.0	[26.8; 73.2]	0.8
Edwards 1994	2	4	50.0	[3.5; 96.5]	0.5
Gulley 1995	2	4	50.0	[3.5; 96.5]	0.5
Joab 1991	11	22	50.0	[29.1; 70.9]	0.8
Gutterrez 1992	20	39	51.3	[35.5; 66.9]	0.8
Rowe 1986	9	17	52.9	[28.8; 76.4]	0.8
Carpenter 2008	173	325	53.2	[47.8; 58.6]	0.9
Minnicelli 2012	33	61	54.1	[41.4; 66.5]	0.8
Akyol 1997	6	11	54.5	[24.3; 83.2]	0.7
Habeeb 2021	22	40	55.0	[39.3; 70.2]	0.8
Chao 1997	10	18	55.6	[32.0; 78.0]	0.8
Navari 2015	17	30	56.7	[38.5; 74.0]	0.8
Okano 1992	4	7	57.1	[18.8; 91.6]	0.6
Ambrosio 2014	41	71	57.7	[46.0; 69.0]	0.8
Hamilton-Dutoit 1993a	11	19	57.9	[34.8; 79.4]	0.8
Reedman 1974	11	19	57.9	[34.8; 79.4]	0.8
Bellan 2005	18	31	58.1	[40.1; 75.0]	0.8
Kabyemera 2013	19	32	59.4	[41.7; 75.9]	0.8
Carbone 1993	3	5	60.0	[14.2; 97.8]	0.6
Mitarnun 2004	3	5	60.0	[14.2; 97.8]	0.6
Barriga 1988	34	56	60.7	[47.5; 73.2]	0.8
Hassan 2008	33	54	61.1	[47.7; 73.8]	0.8
Hirshaut 1973	22	36	61.1	[44.5; 76.5]	0.8
Donati 2006	36	58	62.1	[49.2; 74.2]	0.8
Rea 1994	5	8	62.5	[26.0; 93.1]	0.7
Ouyang 2019	14	22	63.6	[42.3; 82.7]	0.8
Shiramizu 1991	35	54	64.8	[51.5; 77.1]	0.8
Pizza 2008	33	50	66.0	[52.2; 78.6]	0.8
Monteiro 2019	8	12	66.7	[37.2; 90.9]	0.7
Pallesen 1991	2	3	66.7	[6.8; 100.0]	0.5
Rao 2000	28	42	66.7	[51.6; 80.2]	0.8
Syrjänen 1992	20	29	69.0	[50.8; 84.7]	0.8
Tao 1998	7	10	70.0	[37.5; 94.9]	0.7
WG 1996	17	24	70.8	[50.8; 87.6]	0.8
Prevot 1992	10	14	71.4	[44.6; 92.5]	0.7
Hassan 2006	25	35	71.4	[55.2; 85.4]	0.8
Sulitzeanu 1988	10	14	71.4	[44.6; 92.5]	0.7
Klumb 2004	21	29	72.4	[54.5; 87.4]	0.8
Anwar 1995	30	41	73.2	[58.4; 85.8]	0.8
Bingler 2008	3	4	75.0	[21.3; 100.0]	0.5
Granai 2020	18	24	75.0	[55.5; 90.6]	0.8
Henle 1971	120	156	76.9	[70.0; 83.2]	0.9
Klein 1970	15	19	78.9	[57.3; 94.8]	0.8
Olweny 1977	27	34	79.4	[64.0; 91.6]	0.8
Mansoor 1997	8	10	80.0	[48.7; 99.4]	0.7
Ometto 1997	4	5	80.0	[32.7; 100]	0.6
Uccini 2018	100	125	80.0	[72.5; 86.6]	0.9
Xue 2002	4	5	80.0	[32.7; 100.0]	0.6
Pagano 1973	22	27	81.5	[64.3; 94.2]	0.8
Iliyasu 2014	23	28	82.1	[65.4; 94.5]	0.8
Levine 1971	24	29	82.8	[66.5; 94.7]	0.8
Monteiro 2009	10	12	83.3	[56.0; 99.5]	0.7
Nomure 2008	10	12	83.3	[56.0; 99.5]	0.7
Boyle 1991	5	6	83.3	[41.5; 100.0]	0.6
Camilleri-Broët 1995	16	19	84.2	[63.8; 97.7]	0.8
Nonoyama 1975	22	26	84.6	[67.8; 96.4]	0.8
Riverend 1984	6	7	85.7	[48.3; 100.0]	0.6
Westmoreland 2017	76	88	86.4	[78.3; 92.8]	0.9
Araujo 1996	47	54	87.0	[76.6; 94.9]	0.9
Henle 1969	82	92	89.1	[81.8; 94.8]	0.9
Klein 1969	18	20	90.0	[72.1; 99.8]	0.8
Cavdar 1994	29	32	90.6	[77.6; 98.7]	0.8
Gotlieb-Stematsky 1976	11	12	91.7	[67.6; 100.0]	0.7
Tumwine 2010	79	86	91.9	[85.0; 96.9]	0.9
Mutalima 2008	128	138	92.8	[87.8; 96.6]	0.9
Kaymaz 2017	26	28	92.9	[79.6; 99.9]	0.8
Tacyildiz 1998	28	30	93.3	[80.9; 99.9]	0.8
Cavdar 1993	18	19	94.7	[78.7; 100.0]	0.8
Nonoyama 1973	22	23	95.7	[82.2; 100.0]	0.8
Aguilar 2017	260	271	95.9	[93.2; 98.0]	0.9
Geser 1983	51	53	96.2	[89.0; 99.9]	0.9
Lindahl 1974	26	27	96.3	[84.8; 100.0]	0.8
Ndede1 2019	32	33	97.0	[87.4; 100.0]	0.8
Aitken 1994	56	56	100.0	[96.9; 100.0]	0.9
Cool 1997	17	17	100.0	[90.1; 100.0]	0.8
Feng 2007	2	2	100.0	[31.3; 100.0]	0.4
Grässer 1994	3	3	100.0	[50.0; 100.0]	0.5
Guarner 1991	6	6	100.0	[73.0; 100.0]	0.6
Labrecque 1999	46	46	100.0	[96.3; 100.0]	0.9
Liebowitz 1998	3	3	100.0	[50.0; 100.0]	0.5
Niedobitek 1995	17	17	100.0	[90.1; 100.0]	0.8
Parolini 2002	12	12	100.0	[86.0; 100.0]	0.7
Stevens 2001	12	12	100.0	[86.0; 100.0]	0.7
**Random effects model**		**4,798**	**61.0**	**[55.8; 66.1]**	**100.0**

Heterogeneity: $I^2 = 92\%$, $\tau^2 = 0.3187$, $p < 0.01$

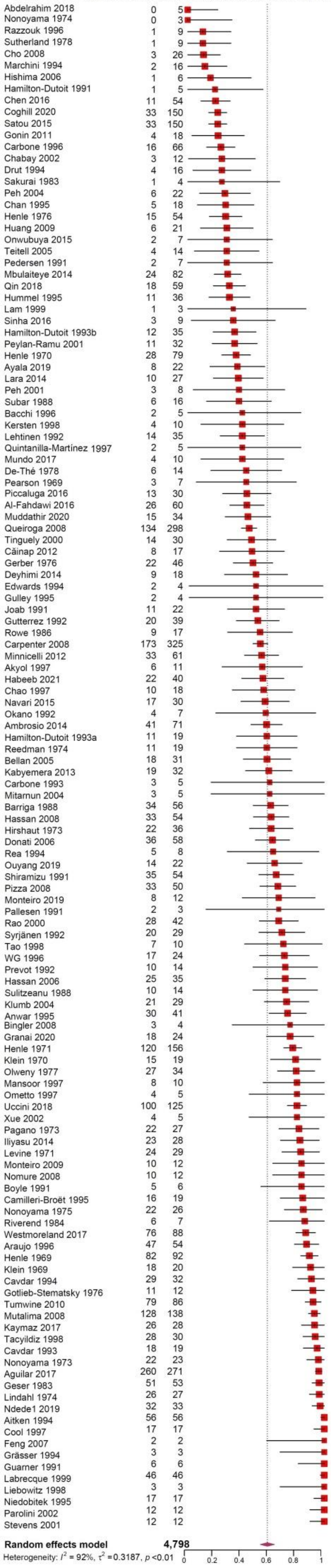

Figure S2. Sensitivity analyses (A) excluding small studies, (B) excluding low- and moderate-quality studies, (C) considering only cross-sectional studies, (D) considering only case-control studies, (E) considering only cohort, (F) considering only studies where the age was less than 18 years old, (G) excluding outlier studies estimating the prevalence of EBV virus in patients with BL.

Table S1. Search strategies

Databases	Search strategies
PubMed	(Burkitt[Title/Abstract] OR Burkitts[Title/Abstract] OR Burkitt's[Title/Abstract] OR "African Lymphoma"[Title/Abstract]) AND (Epstein-Barr[Title] OR EBV[Title] OR "Human Herpesvirus 4"[Title] OR HHV4[Title] OR HHV-4[Title] OR "EB virus"[Title])
Google Scholar	allintitle:(Burkitt OR Burkitts OR Burkitt's OR “African Lymphoma”) (Epstein-Barr OR EBV OR “Human Herpesvirus 4” OR HHV4 OR HHV-4 OR “EB virus”)
Scopus	TITLE(Burkitt OR Burkitts OR Burkitt's OR "African Lymphoma") AND TITLE(Epstein-Barr OR EBV OR "Human Herpesvirus 4" OR HHV4 OR HHV-4 OR "EB virus")
Web of Science	TI=(Burkitt OR Burkitts OR Burkitt's OR “African Lymphoma”) TI=(Epstein-Barr OR EBV OR “Human Herpesvirus 4” OR HHV4 OR HHV-4 OR “EB virus”)

Table S2. Quality assessment of the included cross-sectional studies

No.	Study ID	Questions assessing included cross-sectional studies								Yes (%)
		1	2	3	4	5	6	7	8	
1	Abdelrahim 2018	Y	Y	Y	Y	U	U	Y	Y	75
2	Aitken 1994	Y	Y	Y	Y	U	U	Y	Y	75
3	Akyol 1997	Y	Y	Y	Y	U	U	Y	Y	75
4	Ayala 2019	Y	Y	Y	Y	U	U	Y	Y	75
5	Bacchi 1996b	Y	Y	Y	Y	Y	Y	Y	Y	100
6	Barriga 1988	Y	Y	Y	Y	U	U	Y	Y	75
7	Camilleri-Broët 1995	Y	Y	Y	Y	Y	Y	Y	Y	100
8	Carbone 1993	Y	Y	Y	Y	Y	N	Y	Y	87.5
9	Carbone 1996	Y	U	Y	Y	Y	Y	Y	Y	87.5
10	Chabay 2002	Y	Y	Y	Y	Y	Y	Y	Y	100
11	Chan 1995	Y	Y	Y	Y	U	U	Y	Y	75
12	Chao 1997	Y	Y	Y	Y	U	U	Y	Y	75
13	Chen 2016	Y	Y	Y	Y	U	U	Y	Y	75
14	Cho 2008	Y	Y	Y	Y	U	U	Y	Y	75
15	Cool 1997	U	Y	Y	Y	U	U	Y	Y	62.5
16	Deyhimi 2014	Y	Y	Y	Y	U	U	Y	Y	75
17	Drut 1994	Y	Y	Y	Y	U	U	Y	Y	75
18	Edwards 1994	U	N	Y	Y	U	U	Y	Y	50
19	Feng 2007	Y	Y	Y	Y	U	U	Y	Y	75
20	Geser 1983	Y	Y	Y	Y	Y	Y	Y	Y	100
21	Grässer 1994	U	Y	Y	Y	U	U	Y	Y	62.5
22	Guarner 1991	Y	Y	Y	Y	U	U	Y	Y	75
23	Gulley 1995	Y	Y	Y	Y	Y	Y	Y	Y	100
24	Gutterrez 1992	U	Y	Y	Y	U	U	Y	Y	62.5
25	Hamilton-Dutoit 1991	Y	Y	Y	Y	U	U	Y	Y	75
26	Hamilton-Dutoit 1993a	Y	N	Y	Y	U	U	Y	Y	62.5
27	Hamilton-Dutoit 1993b	Y	N	Y	Y	U	U	Y	Y	62.5
28	Hassan 2008	Y	Y	Y	Y	Y	Y	Y	Y	100
29	Huang 2009	Y	Y	Y	Y	U	U	Y	Y	75
30	Hummel 1995	Y	Y	Y	Y	U	U	Y	Y	75
31	Iliyasu 2014	Y	Y	Y	Y	U	U	Y	Y	75
32	Kaymaz 2017	Y	Y	Y	Y	U	U	Y	Y	75
33	Kersten 1998	Y	Y	Y	Y	U	U	Y	Y	75
34	Kim 2005	Y	Y	Y	Y	U	U	Y	Y	75
35	Labrecque 1999	Y	Y	Y	Y	U	U	Y	Y	75
36	Lam 1999	Y	Y	Y	Y	U	U	Y	Y	75
37	Lara 2014	Y	Y	Y	Y	Y	Y	Y	Y	100
38	Lee 1991	Y	Y	Y	Y	U	U	Y	Y	75
39	Lehtinen 1992	Y	Y	Y	Y	Y	Y	Y	Y	100

1. Were the criteria for inclusion in the sample clearly defined? **2.** Were the study subjects and the setting described in detail? **3.** Was the exposure measured in a valid and reliable way? **4.** Were objective, standard criteria used for measurement of the condition? **5.** Were confounding factors identified? **6.** Were strategies to deal with confounding factors stated? **7.** Were the outcomes measured in a valid and reliable way? **8.** Was appropriate statistical analysis used? Y=Yes; N=No; U=Unclear.

Table S2. Quality assessment of the included cross-sectional studies, continued

No.	Study ID	Questions assessing included cross-sectional studies								Yes (%)
		1	2	3	4	5	6	7	8	
40	Liebowitz 1998	Y	Y	Y	Y	Y	N	Y	N	75
41	Lindahl 1974	Y	Y	Y	Y	U	U	Y	Y	75
42	Mansoor 1997	Y	Y	Y	Y	U	U	Y	Y	75
43	Marchini 1994	Y	Y	Y	Y	U	U	Y	Y	75
44	Mbulaiteye 2014	Y	Y	Y	Y	Y	Y	Y	Y	100
45	Mitarnun 2004	Y	Y	Y	Y	U	U	Y	Y	75
46	Monteiro 2009	Y	Y	Y	Y	U	U	Y	Y	75
47	Monteiro 2019	Y	Y	Y	Y	U	U	Y	Y	75
48	Navari 2015	Y	Y	Y	Y	U	U	Y	Y	75
49	Ndede1 2019	Y	Y	Y	Y	U	U	Y	Y	75
50	Nomure 2008	Y	Y	Y	Y	U	U	Y	Y	75
51	Nonoyama 1973	N	Y	Y	Y	U	U	Y	Y	62.5
52	Nonoyama 1974	N	Y	N	Y	U	U	N	Y	50
53	Nonoyama 1975	Y	Y	Y	Y	Y	Y	Y	Y	100
54	Olweny 1977	Y	Y	Y	Y	U	U	Y	Y	75
55	Ometto 1997	Y	Y	Y	Y	U	U	Y	Y	75
56	Onwubuya 2015	Y	Y	Y	Y	U	U	Y	Y	75
57	Ouyang 2019	Y	Y	Y	Y	U	U	Y	Y	75
58	Pagano 1973	Y	Y	Y	Y	U	U	Y	Y	75
59	Pallesen 1991	Y	Y	Y	Y	Y	N	Y	Y	87.5
60	Peh 2001	Y	Y	Y	Y	U	U	Y	Y	75
61	Peh 2004	Y	Y	Y	Y	U	U	Y	Y	75
62	Piccaluga 2016	Y	Y	Y	Y	Y	Y	Y	Y	100
63	Prevot 1992	Y	Y	Y	Y	U	U	Y	Y	75
64	Queiroga 2008	Y	Y	Y	Y	U	U	Y	Y	75
65	Quintanilla-Mart ńez 1997	Y	Y	Y	Y	U	U	Y	Y	75
66	Rao 2000	Y	Y	Y	Y	U	U	Y	Y	75
67	Razzouk 1996	Y	Y	Y	Y	U	U	Y	Y	75
68	Rea 1994	Y	N	Y	Y	U	U	N	Y	75
69	Reedman 1974	Y	Y	Y	Y	U	U	Y	Y	75
70	Rowe 1986	Y	Y	N	Y	U	U	Y	Y	75
71	Sakurai 1983	Y	Y	Y	Y	U	U	Y	Y	75
72	Shiramizu 1991	Y	Y	Y	Y	Y	Y	Y	Y	100
73	Subar 1988	Y	Y	Y	Y	U	U	Y	Y	75
74	Syrj änen 1992	U	Y	Y	Y	U	U	Y	Y	62.5
75	Tacyildiz 1998	Y	Y	Y	Y	U	U	N	Y	75
76	Tao 1998	Y	Y	Y	N	U	U	Y	Y	62.5
77	Teitell 2005	Y	N	Y	Y	U	U	Y	Y	62.5
78	Tumwine 2010	Y	Y	Y	Y	U	U	Y	Y	75
79	Uccini 2018	Y	Y	Y	Y	U	U	Y	Y	75

1. Were the criteria for inclusion in the sample clearly defined? **2.** Were the study subjects and the setting described in detail? **3.** Was the exposure measured in a valid and reliable way? **4.** Were objective, standard criteria used for measurement of the condition? **5.** Were confounding factors identified? **6.** Were strategies to deal with confounding factors stated? **7.** Were the outcomes measured in a valid and reliable way? **8.** Was appropriate statistical analysis used? Y=Yes; N=No; U=Unclear.

Table S3. Quality assessment of the included case-control studies

No.	Study ID	Questions assessing included case-control studies										Yes (%)
		1	2	3	4	5	6	7	8	9	10	
1	Aguilar 2017	Y	Y	Y	Y	Y	Y	Y	Y	U	Y	90
2	Al-Fahdawi 2016	Y	Y	Y	Y	Y	N	N	Y	U	Y	70
3	Anwar 1995	N	Y	Y	Y	Y	N	N	Y	U	Y	60
4	Araujo 1996	N	Y	Y	Y	Y	U	U	Y	U	Y	60
5	Bellan 2005	N	Y	Y	Y	Y	U	U	Y	U	Y	60
6	Carpenter 2008	Y	Y	Y	Y	Y	U	U	Y	U	Y	70
7	Cavdar 1993	Y	Y	Y	Y	Y	U	U	Y	U	Y	70
8	Cavdar 1994	Y	Y	Y	Y	Y	U	U	Y	U	Y	70
9	Coghill 2020	Y	Y	Y	Y	Y	U	U	Y	U	Y	70
10	De-Thé 1978	Y	Y	Y	Y	Y	U	U	Y	Y	Y	80
11	Gerber 1976	Y	Y	Y	Y	Y	U	U	Y	U	Y	70
12	Gotlieb-Stematsky 1976	Y	Y	Y	Y	Y	Y	Y	Y	Y	Y	100
13	Habeeb 2021	Y	Y	Y	Y	Y	U	U	Y	U	Y	70
14	Hassan 2006	Y	U	Y	Y	Y	U	U	Y	U	Y	60
15	Henle 1969	N	N	Y	Y	Y	U	U	Y	U	Y	50
16	Henle 1970	U	U	Y	Y	Y	U	U	Y	U	N	40
17	Henle 1971	U	N	Y	Y	Y	U	U	Y	U	Y	50
18	Henle 1976	N	N	Y	Y	Y	U	U	Y	U	Y	50
19	Hirshaut 1973	Y	Y	Y	Y	Y	U	U	Y	U	Y	70
20	Joab 1991	U	U	U	Y	Y	U	U	Y	U	Y	40
21	Kabyemera 2013	Y	Y	Y	Y	Y	U	U	Y	U	Y	70
22	Klein 1969	U	N	Y	Y	Y	U	U	Y	U	Y	50
23	Klein 1970	U	N	Y	Y	Y	U	U	Y	U	Y	50
24	Klumb 2004	U	U	Y	Y	Y	U	U	Y	U	Y	50
25	Levine 1971	N	N	Y	Y	Y	U	U	Y	U	Y	50

1. Were the groups comparable other than the presence of disease in cases or the absence of disease in controls? **2.** Were cases and controls matched appropriately? **3.** Were the same criteria used for identification of cases and controls? **4.** Was exposure measured in a standard, valid and reliable way? **5.** Was exposure measured in the same way for cases and controls? **6.** Were confounding factors identified? **7.** Were strategies to deal with confounding factors stated? **8.** Were outcomes assessed in a standard, valid and reliable way for cases and controls? **9.** Was the exposure period of interest long enough to be meaningful? **10**. Was appropriate statistical analysis used? Y=Yes; N=No; U=Unclear.

Table S3. Quality assessment of the included case-control studies, continued

No.	Study ID	Questions assessing included case-control studies										Yes (%)
		**1**	**2**	**3**	**4**	**5**	**6**	**7**	**8**	**9**	**10**	
26	Minnicelli 2012	Y	Y	Y	Y	Y	Y	Y	Y	U	Y	90
27	Muddathir 2020	U	U	Y	Y	Y	U	U	Y	U	Y	50
28	Mutalima 2008	Y	U	Y	Y	Y	Y	Y	Y	U	Y	80
29	Niedobitek 1995	U	N	Y	Y	Y	U	U	Y	U	Y	50
30	Parolini 2002	U	U	Y	Y	Y	U	U	Y	U	Y	50
31	Pearson 1969	U	U	Y	Y	Y	U	U	Y	U	Y	50
32	Riverend 1984	Y	Y	Y	Y	Y	U	U	Y	U	Y	70
33	Satou 2015	Y	U	Y	Y	Y	U	U	Y	U	Y	60
34	Stevens 2001	N	U	Y	Y	Y	U	U	Y	U	Y	50
35	Sulitzeanu 1988	N	U	Y	Y	Y	U	U	Y	U	Y	50
36	Sutherland 1978	N	N	Y	Y	Y	U	U	Y	U	U	40
37	Tinguely 2000	Y	Y	Y	Y	Y	U	U	Y	U	Y	70
38	WG 1996	Y	U	Y	Y	Y	U	U	Y	U	Y	60
39	Xue 2002	Y	U	Y	Y	Y	U	U	Y	U	Y	60

1. Were the groups comparable other than the presence of disease in cases or the absence of disease in controls? **2.** Were cases and controls matched appropriately? **3.** Were the same criteria used for identification of cases and controls? **4.** Was exposure measured in a standard, valid and reliable way? **5.** Was exposure measured in the same way for cases and controls? **6.** Were confounding factors identified? **7.** Were strategies to deal with confounding factors stated? **8.** Were outcomes assessed in a standard, valid and reliable way for cases and controls? **9.** Was the exposure period of interest long enough to be meaningful? **10**. Was appropriate statistical analysis used? Y=Yes; N=No; U=Unclear.

Table S4. Quality assessment of the included cohort studies

No.	Study ID	Questions assessing included cohort studies											Yes (%)
		**1**	**2**	**3**	**4**	**5**	**6**	**7**	**8**	**9**	**10**	**11**	
1	Ambrosio 2014	N	Y	Y	U	U	N	Y	U	N	U	Y	36.36
2	Banatvala 1972	Y	Y	U	U	U	U	Y	U	Y	U	Y	45.45
3	Bingler 2008	Y	Y	Y	U	U	U	Y	U	N	Y	Y	54.54
4	Boyle 1991	Y	Y	Y	U	U	U	Y	Y	Y	U	Y	63.63
5	Căinap 2012	Y	Y	Y	U	U	U	Y	Y	Y	Y	Y	72.72
6	Donati 2006	Y	Y	Y	U	U	U	Y	Y	Y	N	Y	63.63
7	Gonin 2011	U	Y	Y	U	U	U	Y	N	N	Y	Y	45.45
8	Granai 2020	N	N	Y	U	U	U	Y	U	Y	U	Y	36.36
9	Hishima 2006	Y	Y	Y	U	U	U	Y	Y	Y	U	Y	63.63
10	Mundo 2017	Y	Y	Y	U	U	U	Y	Y	Y	N	Y	63.63
11	Okano 1992	Y	Y	Y	U	U	U	Y	Y	Y	U	Y	63.63
12	Pedersen 1991	Y	Y	Y	U	U	U	Y	Y	Y	Y	Y	72.72
13	Peylan-Ramu 2001	Y	Y	Y	U	U	U	Y	Y	N	U	Y	54.54
14	Pizza 2008	Y	Y	Y	U	U	Y	Y	Y	Y	Y	Y	81.81
15	Qin 2018	Y	Y	Y	U	U	Y	Y	U	Y	U	Y	63.63
16	Sinha 2016	Y	Y	Y	U	U	U	Y	Y	Y	N	Y	63.63
17	Westmoreland 2017	Y	Y	Y	Y	Y	Y	Y	Y	Y	Y	Y	100

1. Were the two groups similar and recruited from the same population? **2.** Were the exposures measured similarly to assign people to both exposed and unexposed groups? **3.** Was the exposure measured in a valid and reliable way? **4.** Were confounding factors identified? **5.** Were strategies to deal with confounding factors stated? **6.** Were the groups/participants free of the outcome at the start of the study (or at the moment of exposure)? **7.** Were the outcomes measured in a valid and reliable way? **8.** Was the follow up time reported and sufficient to be long enough for outcomes to occur? **9.** Was follow up complete, and if not, were the reasons to loss to follow up described and explored? **10.** Were strategies to address incomplete follow up utilized? **11.** Was appropriate statistical analysis used? Y=Yes; N=No; U=Unclear; NA: Not applicable.
